# Double-Stapled Peptide Scan Yields Potent Fusion Inhibitors of Respiratory Syncytial Virus

**DOI:** 10.1021/acs.jmedchem.5c02932

**Published:** 2026-05-27

**Authors:** Nadège Pidoux, Logan Roh, Nancy Nicolet, Roger Marti, Adrien Le Rouzic, Clémentine Prompt, Jenna Fix, Stéphane Duquerroy, Félix Rey, Marie-Anne Rameix-Welti, Mathilde Keck, Peggy Barbe, Dominique Garcin, Geneviève Mottet-Osman, Thibaut Larcher, Marie Galloux, Origène Nyanguile

**Affiliations:** † HES-SO Valais-Wallis, Institute of Life Sciences, 111844 HES-SO University of Applied Sciences Western and Arts Switzerland, rue de l’Industrie 19, Sion 1950, Switzerland; ‡ Institute of Chemical Technology, Haute école d’ingénierie et d’architecture Fribourg, HES-SO University of Applied Sciences and Arts Western Switzerland, Fribourg 1700, Switzerland; § 27057 VIM, INRAE, Domaine de Vilvert, Jouy-en-Josas 78350, France; ∥ Université Paris Saclay, Structural Virology Unit, Institut Pasteur, 28 rue du Dr Roux, Paris 75015, France; ⊥ Université Paris-Saclay, Faculté des Sciences, Orsay 91400, France; # Institut Pasteur, Université Paris-Saclay, Université de Versailles St. Quentin, Université Paris Cité, Assistance Publique des Hôpitaux de Paris, Hôpital Ambroise Paré, M3P, UMR 1173 (2I), INSERM, Centre National de Référence Virus des Infections Respiratoires (CNR VIR), Paris 75015, France; ∇ Université Paris-Saclay, CEA, INRAE, Département Médicaments et Technologies pour la Santé (DMTS), SIMoS, Gif-sur-Yvette 91191, France; ○ Department of Microbiology and Molecular Medicine, University of Geneva School of Medicine, CMU, Geneva 1211, Switzerland; ◆ 52880 INRAE, Oniris, UMR 703 Apex, Nantes 44307, France

## Abstract

Respiratory syncytial virus infection (RSV) is a major global health concern, particularly in infants and elderly populations. In this work, we have screened and identified 3 double-stapled peptides derived from a minimal domain of the RSV F heptad repeat, namely **3/4i**, **3/4m**, and **4/4g**, which are potent inhibitors of RSV fusion and remain active against viral escape mutants resistant to small-molecule fusion inhibitors. Our structural activity relationship (SAR) analysis demonstrates that combining a limited set of staples is sufficient to achieve high antiviral potency. X-ray crystallography revealed that the enhanced potency of **3/4i** and **3/4m** primarily arises from strong hydrophobic interactions between the N-terminal staple and the trimeric HR1 coiled coil of RSV F. *In vivo* pharmacokinetic, imaging, and feasibility studies in RSV-infected Balb/c mice further support intranasal administration as a promising route for delivering these stapled peptides to the lung, highlighting their potential as therapeutics against RSV.

## Graphical abstract

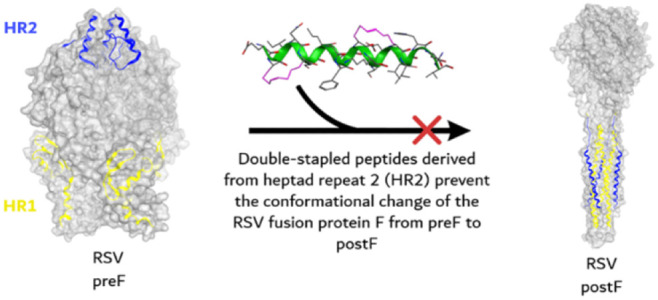

## Introduction

Respiratory Syncytial Virus (RSV) is very contagious and represents the main cause of severe acute respiratory tract illness in young children worldwide, especially for young children with congenital heart disease, chronic lung disease, Down’s syndrome, and neuromuscular diseases. RSV is estimated to cause 33 million cases of lower respiratory infections globally in children under 5 years of age, 3.6 million hospitalizations, and 101 400 RSV attributable deaths, with 97% of these deaths occurring in developing countries.^1,2^ In the United States, the hospitalization rate of children under 5 years of age is of 3 out 1000, resulting in an annual estimated medical cost of 2 billion.^3–5^ RSV-associated lower respiratory infection is also an important disease burden in adults over the age of 65. It is estimated that 1.5 million episodes of acute respiratory infection occur yearly in industrialized countries (no data available globally), resulting in 336 000 hospitalizations and 14 000 in hospital deaths worldwide.^6^

RSV F is a type I fusion glycoprotein located on the surface of the virion that mediates RSV entry into epithelial cells of the upper and lower respiratory tract. Similarly to all type I fusion proteins, RSV F is activated upon cell entry into a metastable prefusion form named “preF”, which contains a hydrophobic fusion peptide, two heptad repeats (HR1 and HR2), and a viral transmembrane domain. Following the insertion of the fusion peptide into the target cell membrane, preF undergoes a dramatic conformational rearrangement to yield a postfusion six-helix bundle hairpin structure named “postF”, whereby 3 HR2 sequences bind in an antiparallel manner to a trimeric HR1 coiled coil.^7,8^ This hairpin structure brings the viral and cell membranes into close apposition, thereby facilitating membrane fusion and subsequent viral entry. The discovery of the means to stabilize preF^9,10^ has been a major breakthrough in the field because neutralizing antibodies produced using stabilized preF are significantly more potent than the former standard of care, palivizumab.^11^ These findings have led to the successful commercialization of 3 novel treatments: 1) Nirsevimab, a monoclonal neutralizing antibody recommended for infants younger than 8 months;^12^ 2) Abrysvo, a subunit vaccine for immunization of pregnant women at 32 through 36 weeks gestational and of individuals 60 years of age and older; and 3) Arexvy, another subunit vaccine recently approved for the prevention of lower respiratory tract infection in adults over 60 years of age.^13^ Despite these remarkable achievements, these products are for prophylactic use only, and there is currently no therapy available to treat infected patients. The most advanced clinical candidate for therapeutic intervention is Ziresovir (AK0529),^14,15^ a small-molecule inhibitor targeting RSV F. However, Ziresovir binds to a 3-fold symmetry cavity in trimeric preF, consistent with the binding mode of other small-molecule fusion inhibitors (JNJ-2408068, JNJ-49153390, BMS-433771, TMC-353121, BTA-9881, JNJ-53718678).^16,17^ As a result, treatment with Ziresovir may result in the emergence of the same escape mutants than other fusion inhibitors as it has already been reported previously in clinical trials.^18,19^

Previously, we reported antiviral peptides^20^ inhibiting RSV fusion, which should be active against small-molecule escape mutants and limit the emergence of new escape mutations. These peptides are derived from the HR2 domain of RSV F and function as dominant negative inhibitors by binding to the transiently exposed HR1 coiled coil in the prehairpin fusion intermediate. Through performing a stapled peptide walk across the HR2 sequence, we identified a minimal domain named peptide **4**, which provided the basis for the discovery of short double-stapled peptide inhibitors of RSV fusion.^20^ Peptide **4** is located at the C terminus (aa 497 to 516) of the HR2 α-helix (aa 485 to 515).^8^ The best peptides, **4ca** and **4bb**, were reported to display EC_50_ inhibitory values of 0.59 and 0.74 μM in HEp-2 cells, respectively, and decreased RSV infection in BALB/c mice. We showed that it was mandatory to incorporate two staples in peptide **4** to be able to inhibit viral fusion.

In this work, we wish to significantly increase the potency of these fusion-stapled peptide inhibitors and perform preclinical investigations with the aim of identifying a suitable candidate for more advanced *in vivo* studies.

## Results

### Double-Stapled Peptide Scan across a HR2 RSV F Minimal Subdomain

We performed an extended double staple scan across peptide **4**.^20^ Four amino acids of peptide **4** were substituted with cross-linking unnatural residues with the aim to yield a peptide containing two staples cross-linking one α-helical turn each (Scheme 1A). To avoid that the non-natural amino acids affect the contact interface with the target protein (gray surface representation in Scheme 1A), we substituted only the amino acids that are not involved in hydrophobic contact with trimeric HR-1 (color coded black in Scheme 1D). Since one turn of an α-helix comprises, on average, 3.6 residues, the unnatural cross-linking amino acids can be incorporated at i, i + 4, or i,i + 3 positions (from N- to C-terminus, respectively). The length of the alkenyl side chain and the configuration of the Cα chain were previously optimized for optimal cross-linking to yield two α-methyl-α-alkenylglycine bisubstituted cross-linking amino acids, i.e., S-pentenyl-alanine (S5) and R-pentenyl-alanine (R5) (Scheme 1B). S5 must be positioned at i + 3 or i + 4, while R5 or S5 is positioned at i, respectively.^21,22^ The N-protected fluorenyl-amino acid analogues are commercially available and can be readily incorporated into the polypeptide backbone. Peptides were synthesized by standard Fmoc/tBu solid phase peptide synthesis, and stapling was performed by Grubbs’ ruthenium-mediated olefin metathesis directly on solid support, followed by TFA-mediated cleavage of all protecting groups and peptide resin release. We screened 31 peptides containing an i,i + 4 or i,i + 3 staple at the N-terminus and an i,i + 4 staple at the C-terminus; these double-stapled peptides were named peptides 4/4 and 3/4, respectively (Scheme 1C). As expected, stapling conferred a significant enhancement of α-helical content to all doubled-stapled peptides tested, with α-helical content ranging from 27.9% to 99.1% (Table S1). To assess the antiviral potency of the double-stapled peptides, HEp-2 cells were infected with recombinant RSV mCherry, and the inhibitory activity of the stapled peptides was quantified by fluorescence, as previously described.^23^ Despite the fact that all staples were incorporated at positions that presumably do not interfere with the binding of peptide **4** to trimeric HR-1, we found that our panel of double-stapled peptides displayed a broad range of inhibitory activity, from potent nanomolar compounds to almost inactive molecules (Scheme 1C).

**Scheme 1:**
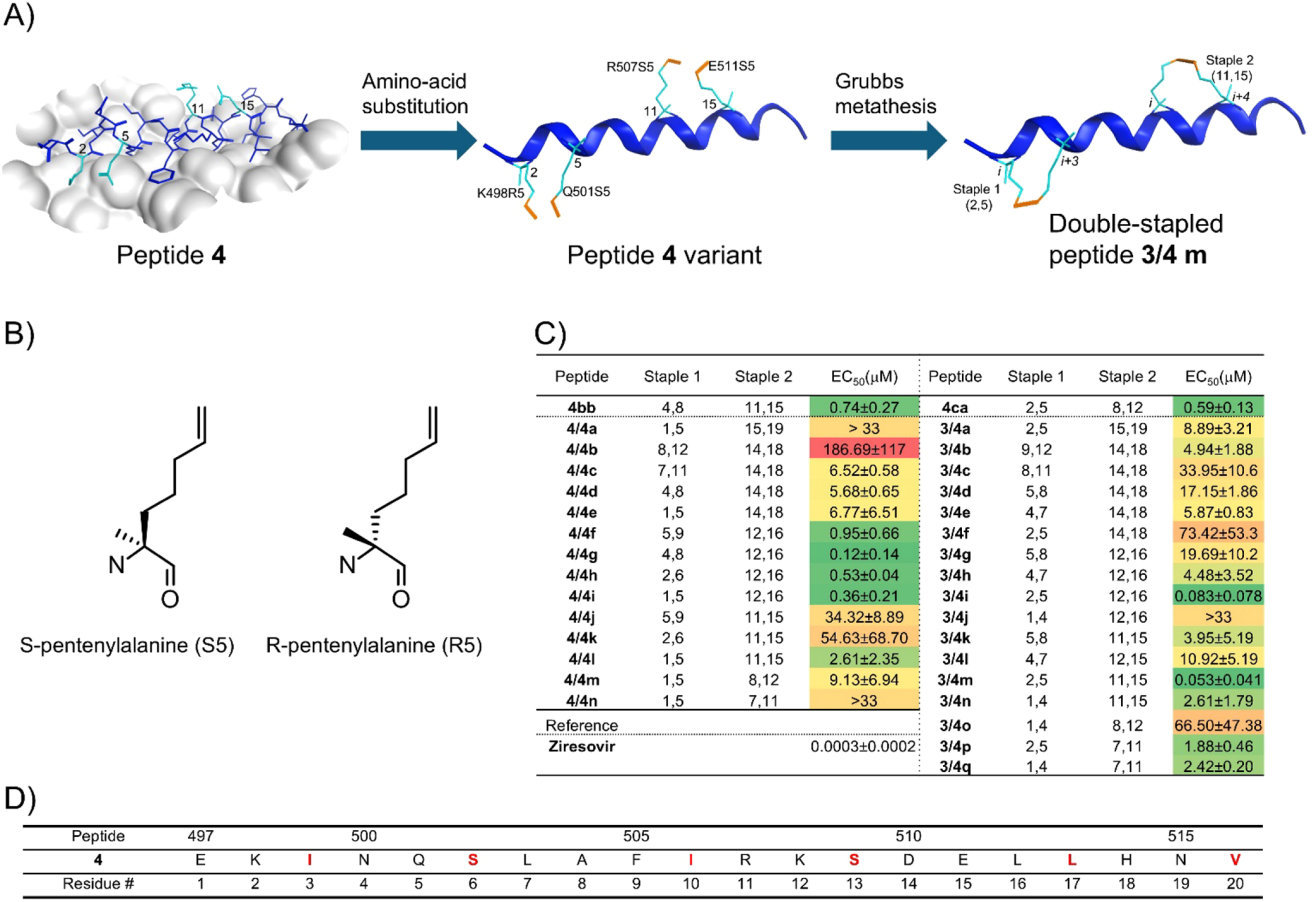
Lead Peptide Inhibitors Identified in this Study[Fn sch1-fn1]

i A) Schematic representation of peptide structure. Peptide **4** is shown in dark blue bound to trimeric HR1 (highlighted in gray surface). In this example, the amino acid residues at positions 2 (K498) and 5 (Q501) (color-coded in cyan) are substituted with R5 and S5 to yield staple 1 (2,5) following Grubbs metathesis, while the positions 11 (R507) and 15 (E511) are both substituted with S5 to yield staple 2 (11,15). The resulting peptide 4 variant was named peptide **3/4m** (the olefinic double bond is color coded in gold). B) Chemical structure of the non-natural amino acid residues that are incorporated into the peptide chain following solid-phase peptide synthesis. C) Diagram showing the position of the staples and the EC50 inhibitory potency of the resulting peptides. D) The sequence of peptide **4** is shown as a one letter code, and each residue is numbered above the sequence according to full-length RSV F (497–516) and below according to their positions in peptide **4** (1–20). Amino acid sequences with residues directly involved in binding to HR1 are color coded in red, and nonessential residues are in black.

Of all peptides tested, only peptides **4/4g**, **3/4i**, and **3/4m** were significantly more potent than peptides **4bb** or **4ca**, displaying EC50 values of 120, 83, and 53 nM, respectively (Scheme 1C and Figure 1A) (no toxicity was observed; data not shown).

**Figure 1:**
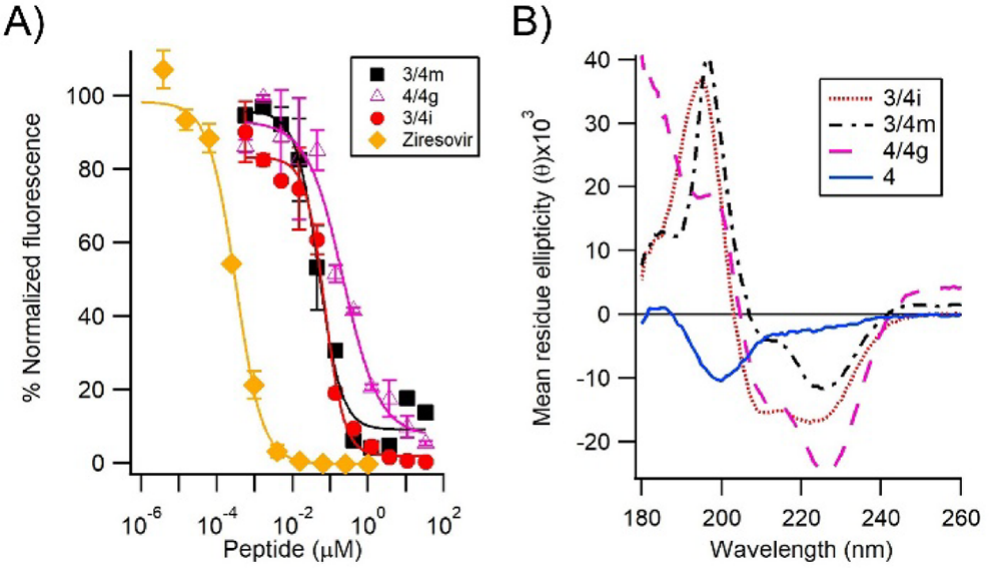
Lead peptide inhibitors identified in this study. A) Antiviral activity of lead double-stapled peptides tested in HEp-2 cells infected with recombinant RSV-mCherry, 48 h postinfection (hpi) (MOI = 0.2). Error bars are standard deviations from duplicates. Each experiment was repeated at least 6 times, and the averaged EC_50_ values are shown. B) CD spectra of lead peptides in comparison with WT parental peptide **4**.

In comparison, the clinical candidate Ziresovir was 100–200-fold more potent (EC50 = 0.3 nM)^14^. The far-UV CD spectrum of peptides **4/4g**, **3/4i,** and **3/4m** (Figure 1B) exhibited the standard characteristics of α-helical secondary structure. In contrast, unstapled peptide **4** appeared to be largely unfolded, with a characteristic minimum at 200 nm.

Interestingly, these 3 lead peptides appear to use only two staple combinations, that is, staple 1 (2,5) or (4,8) combined with staple 2 (11,15) or (12,16) (Scheme 1C and Figure S1). As a matter of fact, our original hit peptide **4bb** carries staple 1 (4,8) and staple 2 (11,15), and **4ca** carries staple 1 (2,5), suggesting that a combination of these specific staples is required to yield potent inhibitors.

On the basis of these observations, we investigated whether the inhibitory activity of peptides carrying staple 1 (2,5) could be further improved by replacing the i,i + 4 of staple 2 with an i,i + 3 staple. A staple 2 peptide scan was performed by inserting i,i + 3 staples at positions that are not involved in deep hydrophobic interactions with trimeric HR1, as discussed above (Table S2).

When the resulting i/i + 3, i/i + 3 double-stapled peptides were tested in RSV-infected HEp-2 cells, we found that most peptides were inactive, except for peptides **3/3c** and **3/3d**. However, they were significantly less potent than the parental lead peptides **3/4i** and **3/4m**, displaying EC_50_ values of 0.86 and 1.71 μM, respectively. Interestingly, staple 2 was inserted at similar positions in **3/3c** and **3/3d** than **3/4i** and **3/4m** [(12,15) and (11,14) compared to (12,16) and (11,15), respectively].

### X-ray Structure of Peptide **3/4m** and **4/4g** in Complex with Trimeric HR1

To better understand the structure–activity relationships (SARs) of this double-stapled peptide series, we decided to elucidate the X-ray structure of peptides **3/4m, 4/4g, and 3/4i** in complex with HR1 (RSV F (159–209), but crystallization trials were unsuccessful for the latter. HR1 was synthesized by SPPS using pseudoproline analogs as described previously^24^ and mixed with an equimolar amount of peptide **3/4m or 4/4g**. The far UV circular dichroism spectra of HR1 at 50 μM in PBS displayed a strong minimum at 209 and 222 nm, indicative of an α-helical structure, supporting the formation of a trimeric coiled coil structure, as is expected for HR1 (Figure S2).^25^ The θ222/θ208 mean residue ellipticity (MRE) ratio was 1.1, consistent with the formation of a trimeric coiled coil structure (usually equal to 0.8–0.9 for a monomeric α-helix). When an equimolar amount of peptide **3/4m** or **4/4g** was added to HR1, the intensity of the minimum at 222 nm further increased significantly, suggesting the formation of a six-helix bundle postfused state.^8^ The θ222/θ208 MRE ratios were 1.1 and 1.4 for the **3/4** and **4/4** complexes, respectively, once again suggesting well-stabilized coiled coil structures. The HR1-**3/4m** or HR1-**4/4g** complex (containing 10% DMSO) was purified to homogeneity by size exclusion chromatography using a Superdex 75 column (GE Healthcare) in TRIS pH 7.4/NaCl 50 mM, and crystals diffracting to 1.6 Å and 1.3 Å were obtained using the vapor diffusion method for HR1–**3/4m** and HR1–**4/4g** complexes, respectively, as described in Materials and Methods (Table S3).

As expected, the peptides pack with trimeric HR1 into a six-helix bundle (Figure 2 left), as seen in the postfusion state of RSV F2 (PDB: 3RRR)^7^ and with a similar construct using the WT sequence (PDB: 1G2C).^8^ A central trimeric core is formed by parallel HR1 helices, and the helical peptide fits in the trimeric HR1 groove in an antiparallel orientation. The peptides are totally helical although the 3 last residues 514-HNV-516 adopt a 3_10_ helix structure in **3/4m**, as it is commonly observed at N- or C-terminal extensions of α-helices,^26^ allowing Val516 to deeply insert into HR1. This C-term region is not resolved in **4/4g**, which ends at H514. The two peptides make a very tight interaction with the two helices making the groove of the HR1 trimeric coiled coil with nearly 950 Å^2^ (530 and 418) and 800 Å^2^ (442 and 375) from a total of 2198 Å² and 2065 Å^2^ of surface area buried in the contact with **3/4m** and **4/4g**, respectively. This large contact is mainly a hydrophobic interaction involving notably residues of the “a” layer on one side of the groove (HR1 helix 2) and those of the “d” layer on the other side (HR1 helix 1, Table S4, Supp. Figure S5). Additional interactions are generated through residues in the neighboring “g” positions and to a lesser extent the “e” positions of the peptides (Table S4, Figure 2 right and Supp. Figure S5). In staple 1 of **3/4m**, the K498R5 methyl group makes a hydrophobic contact with Gly184 of HR1, locking the helical conformation of HR1 around this highly flexible residue (green dotted line in Figure 2, top right). The highly lipophilic all-hydrocarbon staple also contributes to hydrophobic contact with the exposed residues of trimeric HR1 (Figure 2 top right panel, Figure S5 and Table S4). As a matter of fact, the variant K498R5 of staple 1 extends the lipophilic “g” layer of Phe505 and Leu512, contributing to a tight hydrophobic interaction with HR1 (Figure 2B). Overall, the interaction of staple 1 with trimeric HR1 contributes significantly to the enhancement of inhibitory activity of peptide **3/4m** as it has already been reported previously with stapled peptides inhibiting the Mdm2/p53 interaction.^27^

**Figure 2:**
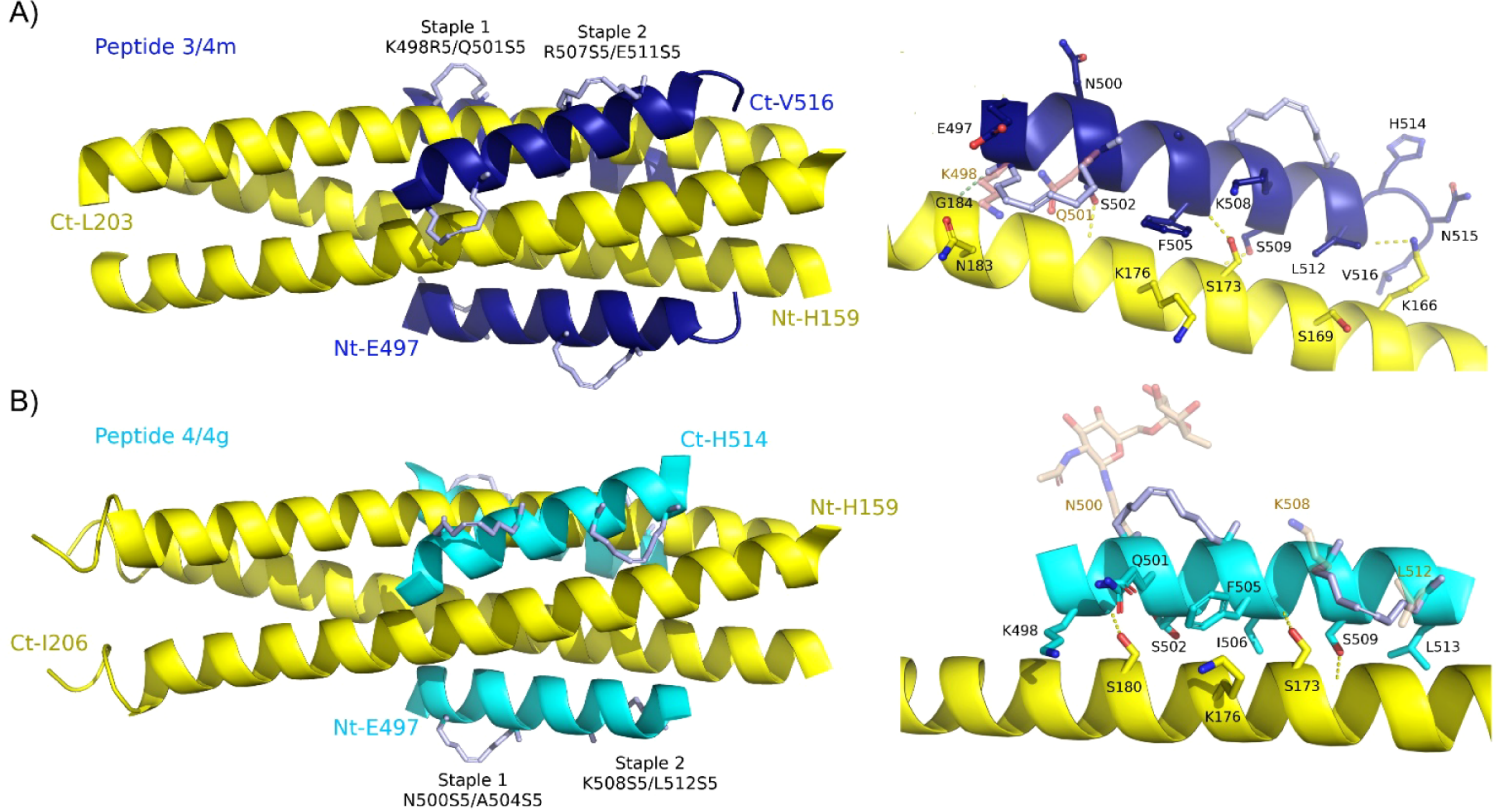
Crystal structure of HR1–**3/4m** and HR1–**4/4g** complexes. Left panel, ribbon diagram of the HR1–**3/4m** complex, top, and of the HR1–**4/4g** complex, bottom. The HR1 helices are shown in yellow, and the **3/4m** and **4/4g** peptides are depicted in dark blue and cyan, respectively. Staples 1 and 2 are shown in tubular structures in light blue. Top right panel, view of the **3/4m** staple 1 binding site. The parental unsubstituted nonstapled residues K498 and Q501 are shadowed in pink. The aliphatic staple K498R5/Q501S5 forms extended hydrophobic contacts with Gly184, Asn183, Leu181, and Ser180. The peptide **3/4m** “g” and “e” layer residues K498R5, Ser502, Phe505, Ser509, and Leu512 are shown, and one HR1 trimeric helix is shown in yellow. Bottom right, view of the **4/4g** staple 2 binding site. The parental unsubstituted nonstapled residues Lys508 and Gln501 are shadowed in pink as well as the ASN500 glycosylation substituted in staple 2. The peptide **4/4g** “g” and “e” layer residues K498, Ser502, Phe505, Ser509, and L512S5 are shown. H-bonds are displayed as yellow dotted lines and labeled. A green dotted line shows the lipophilic contact between K498R5 and Gly184-HR1.

In staple 2, the putative salt bridge occurring between Arg507 and Glu511 of native peptide **4** is replaced by a covalent bond resulting from the metathesis reaction between the amino acid olefinic side chains of R507S5 and E511S5, thereby further enhancing the α-helical secondary structure of peptide **3/4m**. The second staple remains exposed and does not add new interactions with HR1.

In contrast to peptide **3/4m**, staple 1 in **4/4g** remains exposed and does not contribute to interactions with HR1, whereas staple 2 engages in hydrophobic contacts with residues of trimeric HR1. In staple 1, the N500S5 substitution replaces a native glycosylation site present in WT HR2 (Figure 2, bottom right). In staple 2, the covalent linkage formed via metathesis between the olefinic side chains of K508S5 and L512S5 substitutes for the lipophilic moieties of WT residues. For both staples (1 and 2) and in both peptides, the absolute stereochemistry of the double bonds is Z. Finally, although crystallization trials with peptide **3/4i** were unsuccessful, this peptide incorporates staple 1 from **3/4m** and staple 2 from **4/4g**, enabling the construction of a structural model for this third potent peptide (Figure S5). In this model, both staples participate in hydrophobic interactions with residues of the trimeric HR1, consistent with observations from the X-ray structures of the **3/4m** and **4/4g** complexes.

On the basis of a distribution plot of potency versus staple positions (Figure S4), the following SAR conclusions can be drawn: **1)** The insertion of staples at positions 18 and 19 is highly detrimental to potency. This is consistent with the finding that the 3 last residues 514-HNV-516 of peptide **3/4m** adopt a 3_10_ helix structure. **2)** Staples at positions 11 and 12 are detrimental except when they are combined with positions 15–16. These data are consistent with our previous Ala scan mutagenesis experiments, showing that the R507A and K508A variants yield a 175X and 11X EC_50_ fold change.^20^ Presumably, these residues are involved in ionic contacts and must be replaced with another interaction if they are substituted. As discussed above, the R507/E511 putative salt bridge is replaced by staple (11,15). Given the proximity of Leu512 to the HR1 binding groove, it is possible that staple (12,16) makes a new hydrophobic interaction as observed for staple (2,5). Consistent with this, N500S5/A504S5 makes a hydrophobic contact with HR1 in the X-ray structure of the 4/4g complex. **3)** Overall, staple insertion at positions 1 (Glu497) and 7 (Leu504) does not appear to be well tolerated, but the mechanism for this loss of potency is not clear. **4)** In staple 1, the (2,5) and (4,8) positions provide the highest ratio of potent peptides. This is consistent with the hydrophobic contact of K498R5/Q501S5 observed with HR1 in the X-ray structure of the 3/4m complex. For staple (4,8), the mechanism remains to be elucidated.

### In Vitro and Ex Vivo Characterization of Peptide **3/4m**

To assess if peptide **3/4m** is capable of inhibiting escape mutants resulting from small-molecule fusion inhibitors, we assessed its capacity of inhibiting the S398L/K394R double mutant. The S398L escape mutant was observed in a phase 2a human challenge study against presatovir, a fusion inhibitor discovered by Gilead (also named GS-5806).^18^ The K394R mutation was identified in an *in vitro* cellular resistance study against BMS-433771, a potent inhibitor developed by Bristol-Myers.^28^ The S398L/K394R mutant was incorporated into the rRSV-mCherry reporter gene by reverse genetics.^23^ As expected, both RSV-mCherry WT and S398L/K394R variants were inhibited by peptide **3/4m** with similar potency, in contrast to GS-5806 and Ziresovir, whose inhibition was completely abrogated against the double resistant mutant (>38 000-fold change relative to wild-type rRSV-mCherry, Figure 3A). These data suggest that the inhibition of HR2-derived peptides should not be affected by escape mutants emerging from small-molecule fusion inhibitors, given that most small molecules appear to bind to the same preF binding pocket (Figure 3B).^16,17^

**Figure 3:**
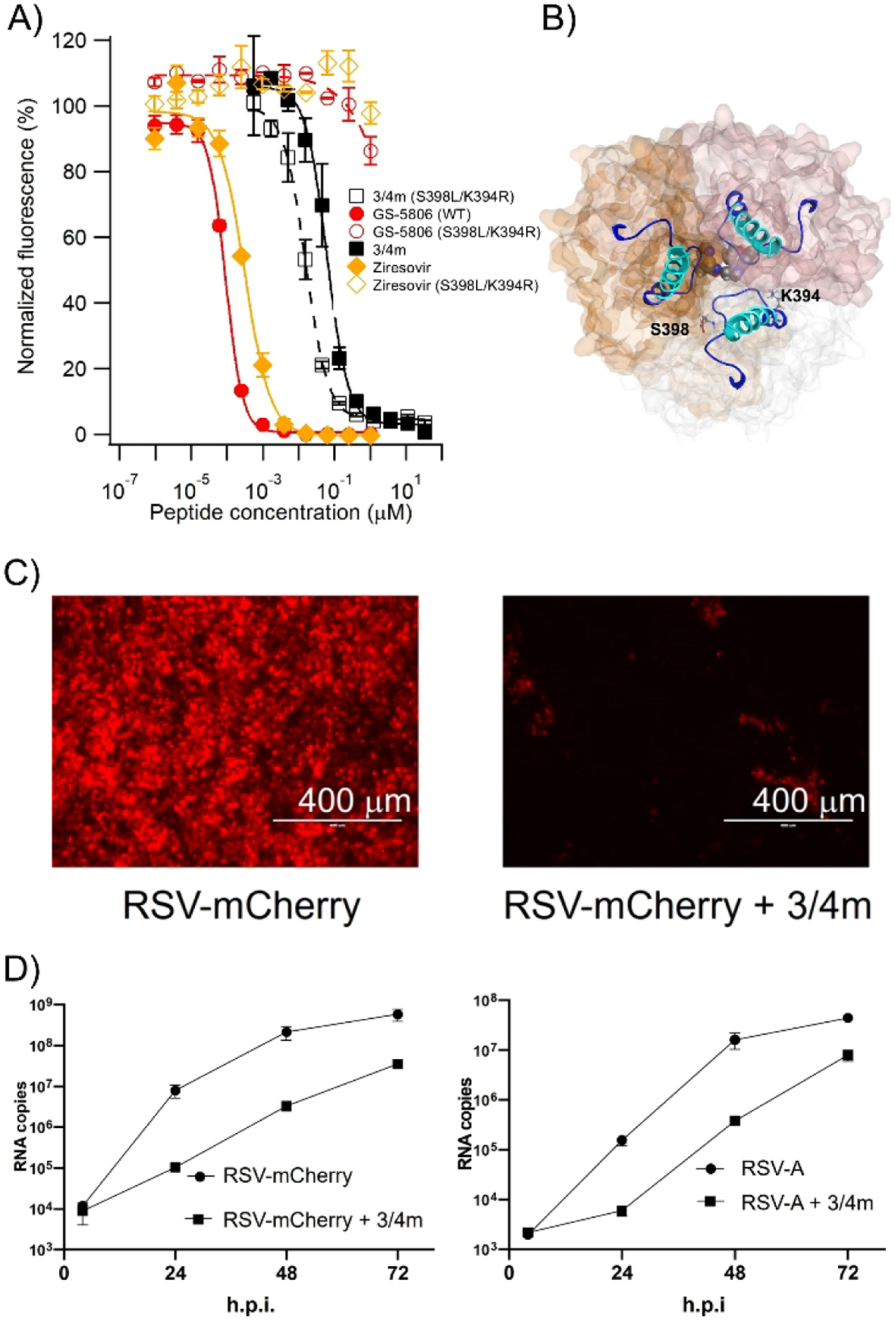
Inhibition of RSV F small-molecule escape mutant and SmallAir^TM^ RSV inhibition assay. A) Inhibition of rRSV-mCherry WT and S398L/K394R variant infection by lead double-stapled peptide **3/4m,** GS-5806, and Ziresovir in HEp-2 cells. Error bars are standard deviations from duplicates. B) Top view of the X-ray structure of BMS-433771 bound to preF (PDB code 5EA7). The 3 RSV F protomers are shown as surface representations in gray, gold, and pink. The 3 HR2 domains are shown as ribbon representations, with peptide 4 color coded in cyan. BMS-433771 is shown in a sphere model, while Lys394 and Ser398 are shown in a ball and stick. C) SmallAir tissues were infected with 1 × 10^4^ pfu of rRSV-mCherry and treated with vehicle or 10 μM of peptide 3/4m. Top panel, mCherry visualization 72 h post-infection. Scale bar, 400 μm. D) SmallAir tissues were infected with 1 × 10^4^ pfu of an RSV-A clinical isolate with or without 10 μM of peptide **3/4m**. Quantification of RSV replication by RT-qPCR performed on nucleic acids extracted from daily collected apical washes of infected tissues incubated in the presence and absence of the antiviral peptide. The gene encoding for RVS N was amplified.^36^ *h.p.i.: hours post-infection.*

Next, peptide **3/4m** was tested in human airway epithelium (HAE) primary cells.^29–35^ HAE presents a structure identical to the human airway pseudostratified layer, including basal cells, goblet cells, and ciliated cells. In addition, the tissue closely mimics the morphology and function of the human respiratory mucosa, such as mucus secretion, ion transport, metabolic activity, and mucociliary clearance, which allows to test antivirals in conditions very close to the human respiratory environment. Human reconstituted airway epithelium originating from the lower (SmallAir) respiratory tract was infected with rRSV-mCherry and concomitantly treated with peptide **3/4m** or vehicle, and the production of mCherry was monitored over time by quantifying the level of mCherry fluorescence or by RT-qPCR of the RNA sequence encoding the nucleoprotein N.^36^. As shown in Figure 3C, peptide **3/4m** efficiently blocked viral infection with a percentage of inhibition higher than 90% even 3 days post-infection, indicating that almost no virus was able to infect the SmallAir tissues. Peptide **3/4m** was also very potent against a clinical isolate derived from strain A, although to a slightly lower extent (Figure 3D, >80% inhibition 3 days post-infection). Lastly, we found that peptide **3/4m** could inhibit a clinical isolate derived from strain B in A549 cells (EC_50_ = 0.63 μM, data not shown).

### In Vitro Stability of Peptide Leads **3/4i**, **3/4m**, and **4/4g**

Peptides are usually susceptible to degradation by proteases *in vivo*, such as luminally secreted enzymes (pepsins, elastase, and others) and brush border membrane-bound enzymes (aminopeptidases, carboxypeptidases, and endopeptidases).^37^ To compare the proteolytic stability of our double-stapled peptide leads, **3/4i**, **3/4m**, and **4/4g,** to peptide **4bb** and **4** (the original stapled peptide hit from our former study and the corresponding wild-type sequence), they were incubated with trypsin, chymotrypsin, and mouse serum, and the amount of remaining peptide was quantified at different time points by LC/MS–MS. As expected, peptide **4** was rapidly degraded by trypsin and chymotrypsin (Figure 4A-B) with a half-life of 4.91 and 4.25 min, respectively (Table S5). In contrast, the stapled peptides were much less susceptible to trypsin degradation. These results were not surprising because the putative trypsin cleavage sites in peptide **4** are all located at the hydrophilic, noninteracting side of peptide **4** (C-terminal side of Lys or Arg,^37^ i.e., K498, R507, and K508) and were replaced by the staples in our lead peptides. More specifically, we found that the resistance to degradation against trypsin decreased in the order of **4/4g**, **3/4i**, and **3/4m** (*t*_1/2_ of 91.98, 40.91, and 18.15 min, respectively, Table S3). The susceptibility to trypsin degradation of **4bb** (23.32 min) was higher than that of **3/4i**.

**Figure 4:**
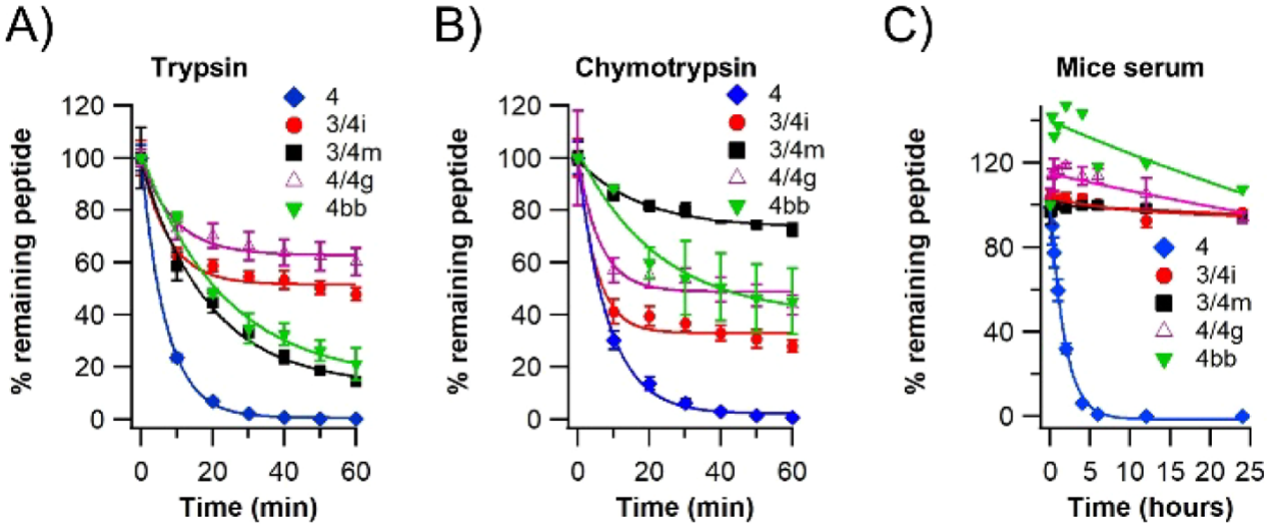
Proteolytic stability assay. Susceptibility of double-stapled peptides **3/4i**, **3/4m,** and **4/4g** in comparison to peptides **4bb** and **4** toward degradation by A) trypsin, B) chymotrypsin, and C) mouse serum, in comparison to peptide **4**, i.e., RSV F (497–516).

These data are unexpected, given that **4/4g** contains two trypsin cleavage sites (Arg507 and Lys498), while **3/4i** and **3/4m** display only one cleavage site (Arg507 and Lys508, respectively). The dramatic increase of stability of **4/4g** may be due to its high helical content (99%, Table 1) since the catalytic site of trypsin must bind to the substrate in an extended conformation of the polypeptide. Additionally, proteases bind to the main chain of the substrate, and the Cα methyl groups of the non-natural amino acid used for the staples may hinder binding.

The resistance to degradation against chymotrypsin was weaker than against trypsin for peptides **4/4g** and **3/4i** (*t*_1/2_ of 36.36 and 20.13 min, respectively), which is expected since both stapled peptides contain more chymotrypsin (C-terminal side of Tyr, Phe, Trp, Leu, i.e., Leu503, Phe505, and Leu513) than trypsin cleavage sites.^37^ The susceptibility to chymotrypsin degradation of **4bb** (43.61 min) was lower than **3/4i**. Unexpectedly, peptide **3/4m** was significantly less susceptible to degradation by chymotrypsin (*t*_1/2_ of 108.3 min) despite the presence of a higher number of potential chymotrypsin cleavage sites (Leu503, Phe505, Leu512, Leu513). Since peptides **3/4i** and **3/4m** carry the same substitutions at staple 1 and peptides **3/4i** and **4/4g** carry the same substitutions at staple 2 (Scheme 1C), we hypothesize that the increased resistance of peptide **3/4m** may be due to the R507S5/E511S5 staple 2, which is unique to peptide **3/4m** and may somehow prevent enzyme accessibility to the C-terminal amide bond of Leu512 and/or Leu513. Lastly, when our double-stapled peptides were incubated in the presence of mouse plasma at 37 °C, they did not show any degradation over the entire time course (>24 h; Figure 4C). These data suggest that the incorporation of staples endows the peptide with resistance to proteolytic cleavage *in vivo*. Consistent with this, unstapled peptide **4** was rapidly degraded in mouse plasma (1.25 h).

### In Vivo Studies of Peptides **3/4i**, **3/4m**, and **4/4g**

To investigate the pharmacokinetic properties of our leads, 200 μL of each peptide at 98 μM in 5% DMSO/PBS pH 7.4 was administered intravenously (IV) to Balb/c mice, and the peptide content in blood was quantified by LC–MS/MS at different time points after administration. Peptide **3/4i** had the best stability and clearance profile after IV administration (Figure 5), displaying a *t*_1/2_ half-life of ∼3.7 h and a plasma clearance of ∼24 h (Table S6). As the site of RSV infection is in the upper and lower respiratory tract, mice lungs were removed 1 h after peptide IV administration, and we attempted to extract the peptide for quantification by LC–MS/MS. The concentration of peptides **3/4i**, **3/4m,** and **4/4g** were estimated to be 1074.6 ± 357.4, 2752.7 ± 2472.5, and 1039.8 ± 449.5 ng/g of lungs, respectively (data not shown).

**Figure 5:**
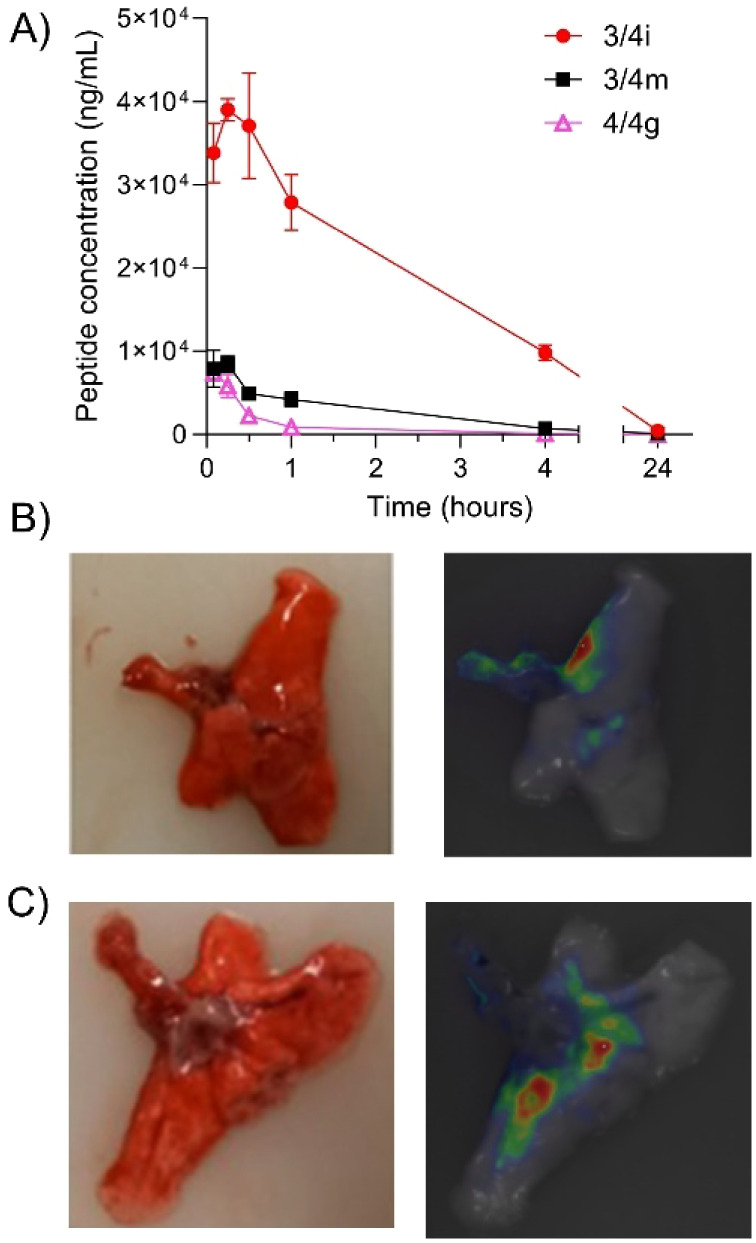
*In vivo* pharmacokinetic studies of lead peptides and pulmonary distribution of peptide **3/4i**. A) Plasma concentrations of lead peptides after intravenous injection at 2.5 mg per kilogram of body weight in female Balb/cJRj mice (*n* = 3 per group). Peptide concentrations from the plasma samples were analyzed by LC–MS/MS, and individual plasma concentration–time profiles were subjected to PKSolver analysis.^38^ B, C) Pulmonary distribution after intranasal administration of sulfo-cyanine-labeled peptide **3/4i** at 2.5 mg per kg of body weight in female Balb/cJRj mice (*n* = 2). Fluorescence molecular tomography 3D scans of the lung outside B) and inside C).

However, given the high variability of our data, probably due to the complexity of extracting peptides from the lung matrix, these results may not be reliable. Next, we attempted to study the pharmacokinetics of peptide **3/4i** following intranasal administration (IN) as this lead showed better exposure under IV administration. 25 μL of peptide at 293 μM in 5% DMSO/PBS pH 7.4 was administered to Balb/c mice, and *in vivo* pharmacokinetics profiling showed that the exposure of peptide **3/4i** in blood was very low, with a maximum amount of approximately 30 ng/mL at 0.5 h (Figure S6). These data suggest that this peptide does not cross the air–blood barrier. However, RSV infection is not systemic but very localized to the lungs and nasal cavity since the virus buds on the apical surface of epithelial cells. Therefore, the intranasal route seems to be the most relevant way of administration since the activity of peptide **3/4i** must act in the lung lumen.

Second, we administered peptide **3/4i** either by intravenous or intranasal route, and we measured by LC–MS/MS the concentration of the peptide in the lungs 1 h after administration (Figure S7). Before the lungs were collected, the mice were perfused in order to wash the tissue samples of the circulating blood. This washing step ensures that only the peptide that entered into the epithelial cells of the lungs is detected. We observed a very low exposure of peptide **3/4i** in the lungs after both IV and IN administration, with a mean amount of approximately 1075–1630 ng/g of lungs, representing only 1–2% of the administered dose. These data suggest that peptide 3/4i does not cross the lung epithelial barrier. Therefore, it can be concluded that peptide 3/4i remains in the mucus at the viral budding site.

To address this issue, we performed noninvasive *in vivo* imaging using the PerkinElmer fluorescence molecular tomography (FMT) technology. Sulfo-cyanine 7 was attached to the N-terminus of peptide **3/4i**, and the peptide was administered intranasally to Balb/c mice. In this experiment, the trachea and lungs were collected without any perfusion in order to observe the peptide directly in the lung lumen via FMT. These studies revealed that peptide **3/4i** is well-exposed in the lung lumen (Figure 5B-C), suggesting that intranasal administration may provide sufficient exposure in the lungs to inhibit RSV infection in Balb/c mice.

### In Vivo Antiviral Activity of Peptide **3/4i** in an RSV Mouse Model

Next, we assessed the antiviral activity of our lead stapled peptide **3/4i** in an RSV mouse infection model.^23^ Balb/c mice were treated by intranasal (IN) administration of peptide **3/4i** or vehicle, followed by IN inoculation of a recombinant human RSV encoding the gene for firefly luciferase, rHRSV-Luc.^23^ The mice were treated once daily and were anesthetized at 2- and 4-day postinfection (dpi) to quantify viral replication using an *in vivo* imaging system (IVIS). As shown in Figure 6, a significant reduction of the bioluminescence signal was detected at both 2 and 4 dpi in mice treated with peptide **3/4i** compared to that detected in the untreated control group. More specifically, at 2 dpi, this reduction was mainly visible in the nose of mice, which is the main replication site at this time point, but the reduction of RSV replication was observed in both the nose and lungs at 4 dpi (Figures 6A and S3). It is noteworthy that daily monitoring of mouse body weights did not show any significant weight loss in animals (data not shown). This result is not surprising since mice infected by RSV under experimental conditions similar to those used in the present study did not show clinical symptoms, but this also suggests the absence of major toxicity upon treatment with peptide **3/4i**. At the end of the experiment, mice lungs were collected to perform histological analysis (Figure 6B). In untreated infected mice, RSV infection led to multifocal extensive marked interstitial pneumonia, characterized by a diffuse thickening of the alveolar walls with mixed inflammatory cells. Mild perivascular cuffing sometimes associated with focal hemorrhages and mild bronchitis with intraluminal necrotic debris were also observed in 6 and 5 out of 6 animals tested, respectively (Figure 6B). In contrast, the intensity of interstitial pneumonia decreased upon the inoculation of peptide **3/4i**. Consistent with this observation, no treated animal displayed vascular lesions, and 5 out of 6 displayed only minimal bronchial lesions. Individual observations of each RSV-infected mouse are listed in Table S7. These observations confirm the antiviral effect of peptide **3/4i** observed in the cellular antiviral assays. Altogether, these data show that stapled peptide **3/4i** is capable of inhibiting RSV infection *in vivo*, without major signs of *in vivo* toxicity under our conditions.

**Figure 6:**
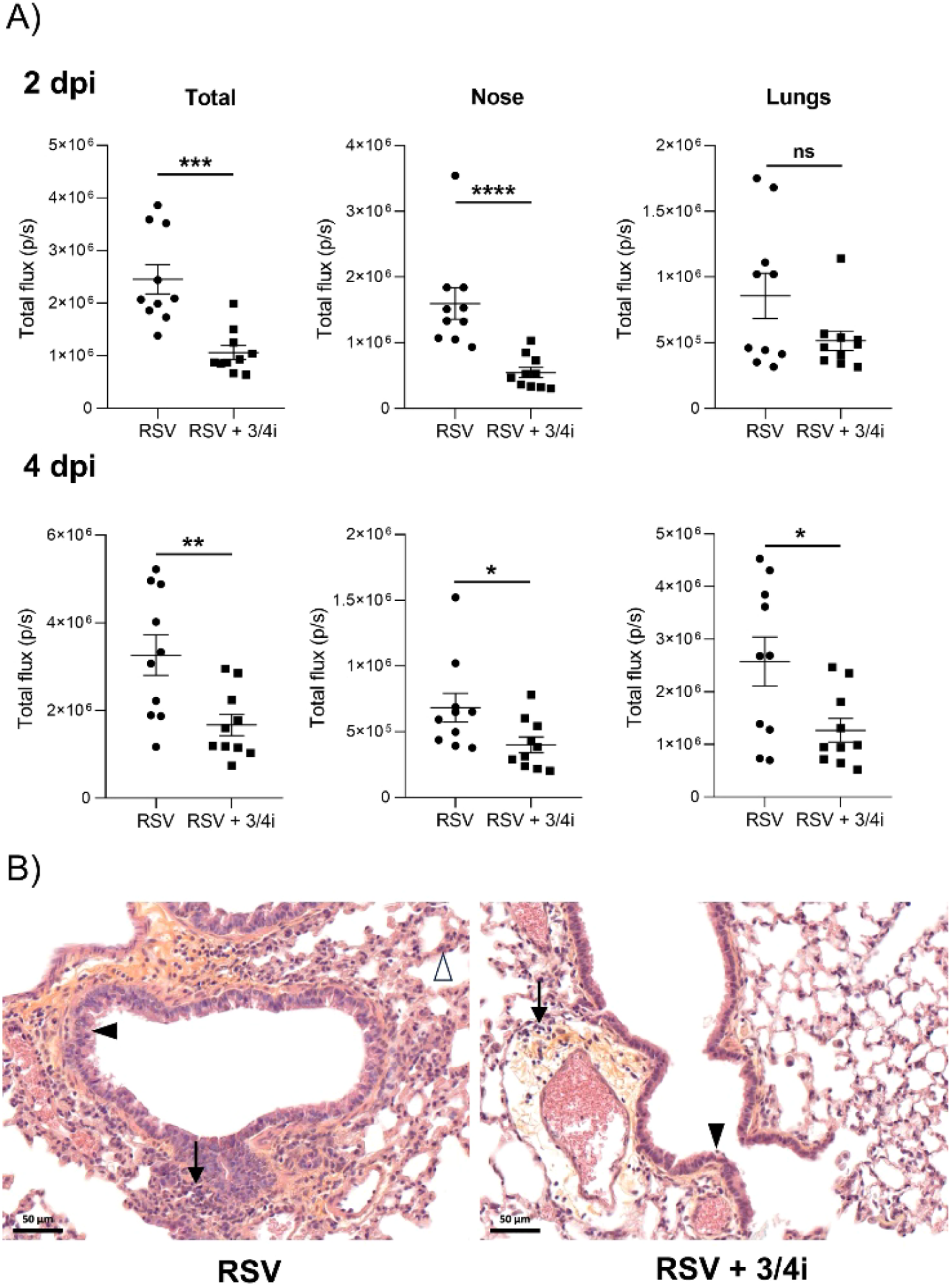
Protective effect of peptide **3/4i** on RSV infection in mice. 8 week-old female Balb/c mice were treated intranasally with 1.17 mg/kg peptide **3/4i** or vehicle. Groups of 10 mice were treated on day 0 individually with either 50 μL PBS or 50 μL peptide **3/4i** (200 μM) in PBS containing 0.8% DMSO and infected 10 min later by 60 μL rHRSV-Luc (8 × 10^4^ PFU per mouse). The administration of peptide **3/4i** was repeated daily until day 3. A) Bioluminescence was measured at 2 and 4 days post-inhalation (dpi) by intranasal administration of 50 μL of D-luciferin. Total bioluminescence, or bioluminescence in the nose and lungs, was quantified for each animal at 2 and 4 dpi. Each dot represents one animal. Data represent mean ± SEM of a single experiment. Two-tailed Mann–Whitney tests were performed to compare untreated and each condition. * *P* < 0.01, ** *P* < 0.002, *** *P* < 0.0002. B) HES staining of mice lungs treated with either vehicle (PBS-0.8% DMSO) or peptide **3/4i**. Scale bar: 50 μm. Left panel (RSV), bronchial epithelium was pluristratified (◀) and enriched in mucus cells (metaplasia). The bronchus and the vessels were surrounded by some clusters of inflammatory cells (↓). Note the diffuse mild thickening of alveolar walls (Δ) compared to the normal ones in treated animals. Right panel (RSV + **3/4i**): epithelium was monostratified with very few isolated desquamated cells in the bronchial lumen (▼). A few inflammatory cells were present around blood vessels (↓).

## Discussion and Conclusions

Here, we have screened double-stapled peptides derived from a minimal HR2 domain of RSV F (497–516), namely peptide **4**. Of the 37 peptides that were tested, we identified 3 lead peptides, **3/4i**, **3/4m,** and **4/4g**, that are capable of inhibiting RSV fusion in cellular antiviral assays with nanomolar potency. We have shown that the inhibitory activity of these lead peptides is not affected by small-molecule fusion escape mutants. Using X-ray crystallography, we showed that the increase of potency of these peptides is mainly due to the introduction of a staple located at the N-terminus of the peptide, which makes a tight hydrophobic interaction with trimeric HR1. On the other hand, we found that the C-terminal part of the peptide folds into a 3d_10_ helix, explaining why the incorporation of staples at the C-terminus of peptide **4** had a negative impact on inhibition. The results of our SAR studies show that the best staple combination to gain high potency is staple 1 (2,5) or (4,8) combined with staple 2 (11,15) or (12,16). This work illustrates that despite having a receptor/ligand X-ray structure available, rational staple design is not sufficient to identify the most potent dominant negative inhibitor and requires a semicombinatorial approach. In fact, if one excludes the six residues critical for binding to HR1 (Table S1) to design the stapled peptides, 103 double-stapled peptides are possible with the 14 remaining residues in peptide 4 (equal to 9*n* ^2^ – 177*n* + 920)/2; *n* being the number of amino acids in the sequence) if only S5 is used for stapling. This highlights the hardwork that may be required for lead optimization, not taking into account the substitution by non-natural amino acids and/or further truncation of the peptide.

In a preliminary *in vivo* assessment study of our lead peptides, **3/4i** was found to have the best pharmacokinetic profile when administered intravenously to Balb/c mice. The concentration of **3/4i** was approximately 3 μM in blood at 8 h post-administration, a concentration 30 000-fold higher than what is required for achieving 50% inhibition in cellular antiviral assays (EC_50_ = 0.083 μM), thereby suggesting sufficient exposure for efficacy by the intravenous route. However, despite this good exposure, no significant inhibition of RSV infection could be observed when **3/4i** was administered through this route (data not shown), probably due to the difficulty of the peptide in crossing the blood–air barrier. For the treatment of respiratory diseases such as RSV, transmucosal delivery (buccal, nasal, or pulmonary) offers several advantages over systemic delivery.^39^ Our fluorescence molecular tomography imaging studies suggest that **3/4i** is well exposed in the lungs when administered intranasally. Consistent with this observation, intranasal administration of **3/4i** to Balb/c RSV-infected mice resulted in approximately one log decrease of viral load. However, as we were not able to reliably quantify **3/4i** in the lungs of infected mice, it is unclear if the exposure of **3/4i** is sufficient to reach optimal efficacy. As a matter of fact, transporting peptides through the mucus barriers can be hindered because of the intrinsic properties of peptides that make it difficult to cross the gel mucus layer, in particular because of the high lipophilicity of the hydrocarbon staples.^37^ Mucus is a complex mixture of lipids, organic components, inorganic ions, enzymes, bactericidal proteins, and mucin glycoproteins that form a protective layer at the surface of the respiratory mucosa. As the mucin protein backbone and sugars can create numerous binding sites, they can strongly interact with peptides depending on their charge arrangements.^40^ Degradation of peptides can also occur in the respiratory tract as proteases, metalloproteinases, neutrophil serine proteases, and other enzymes can influence the effect of inhaled peptides.^41^ To improve targeted peptide delivery to lungs, nanotechnology-based drug delivery systems have recently gained a great interest,^42^ achieving some success against viral infections in preclinical models.^43^ Such technologies may help to further develop the double-stapled peptides described in this study.

## Experimental Section

### Materials and Methods

#### Reagents and Solvents

Fmoc-protected amino acids, *N*,*N*-diisopropylcarbodiimide (DIC), OxymaPure, piperazine, and Fmoc-Rink Amide AM resin were purchased from Merck KGaA (Darmstadt, Germany). Solvents for synthesis, deprotection reagents, and cleavage reagents used were of synthesis grade and purchased from Merck or Fisher Scientific AG (Reinach, Suisse). Amino acids were purchased from Bachem AG (Bubendorf, Switzerland), Merck KGaA, and Aapptec (Louisville, U.S.A.). Solvents and other chemicals used for high-performance liquid chromatography (HPLC) and ultraperformance liquid chromatography–mass spectrometry (UPLC-MS) were of HPLC reagent grade and purchased from Macherey-Nagel (Düren, Switzerland). (*S*)- and (*R*)-*N*-Fmoc-2-(4’-pentenyl)­alanine (S5 and R5, respectively) were purchased from Okeanos (China).

#### Cells and Viruses

HEp-2 cells (ATCC number CCL-23) were maintained in Eagle’s minimum essential medium (EMEM) supplemented with 10% fetal calf serum (FCS), 2 mM l-glutamine, and penicillin-streptomycin solution. Recombinant human RSV strains corresponding to the RSV Long strain expressing either the mCherry or the luciferase protein (rHRSV-mCherry and rHRSV-Luc, respectively) were amplified and titrated as previously described.^20^ All experiments with RSV were carried out in biosafety level 2 facilities. The K394R and S398L mutations were introduced into the reverse genetic vector used for rHRSV-mCherry recovery^20^ using classical mutagenesis methods. The sequences were verified by sequencing. The mutant virus was rescued as previously described^20^ and further amplified on HEp-2 cells at 37 °C.

#### Peptide Synthesis

##### A) Synthesis

Peptides were synthesized by Fmoc/tBu solid-phase peptide chemistry on a Rink Amide AM resin (loading 0.57 mmol/g) using a Liberty Blue automated microwave peptide synthesizer (CEM Corp., Kamp–Lintfort, Germany), following a standard protocol. Each coupling was performed for 4 min at room temperature (RT), using 0.2 M Fmoc amino acid (5 equiv) preactivated with 0.5 M DIC (5 equiv) and 1 M OxymaPure (5 equiv) in DMF. A double coupling was performed for the Fmoc amino acid that follows the S5 or R5 residues required for stapling. A double coupling was also used for His residues, with a lower heating temperature (50 °C) and a longer coupling time (10 min). Fmoc removal was performed with 10% v/v piperazine in DMF, and N-terminal acetylation was achieved with a mixture of 10% pyridine/10% acetic anhydride/40% NMP/40% dichloromethane. The metathesis was then performed under constant nitrogen degassing in a 2 mL solution containing 10 mM first-generation Grubbs’ catalyst in dichloroethane (DCE). The metathesis was performed once or twice for 2 h at RT.

##### B) Cleavage

After the completion of the synthesis, peptides were deprotected and cleaved from the resin manually with TFA under gentle agitation over a period of 1.5 h at RT in the presence of scavengers (standard cleavage solution: TFA/TIS/water 90:5:5). After filtration and evaporation of most of the cleavage solution under a stream of nitrogen, the crude peptides were precipitated by the addition of cold diethyl ether, centrifuged, and washed with cold diethyl ether. The resulting amorphous powder was dried, dissolved in ultrapure water/ACN, frozen, and lyophilized.

##### C) UPLC/MS Analysis

Reactions were monitored by liquid chromatography–mass spectrometry (LC–MS). LC–MS was carried out by Reversed Phase-UPLC MS (Agilent InfinityLab LC/MSD XT) on a Kinetex 1.7 μm XB-C18 100 Å, LC column, 50 × 2.1 mm (Phenomenex Helvetia), using a mixture of solvent B (acetonitrile with 0.1% TFA) and solvent A (water with 0.1% TFA), employing a 2%–98% gradient at a flow rate of 0.617 mL/min for 4 min. The peptide signal was monitored at 214 nm.

##### D) Purification

Peptide purifications were performed by Preparative Reversed Phase-HPLC (Agilent 1260 Infinity II Preparative) on a Kinetex 5 mm XB-C18 100 Å, 100 × 21.2 mm column (Phenomenex Helvetia, Basel, Switzerland) by applying a preselected gradient of solvent B (described in the UPLC-MS section above) at a flow rate of 35 mL/min for 25 min. The peptide absorbance was monitored at 214 nm. Fractions were analyzed by UPLC-MS prior to the lyophilization step. All compounds are >95% pure by UPLC.

### CD Spectroscopy

The circular dichroism spectra were acquired on a Chirascan spectropolarimeter. The samples were prepared in 10 mM phosphate buffer, pH 7.5, at a peptide concentration of 50 μM. Data were recorded at 25 °C by stepscan from 180 to 260 nm in a 0.5 mm path length quartz cell using 1 nm wavelength increments and a response time of 1 s. Each spectrum was an average of three measurements and was subtracted from the buffer baseline. The data were converted to per-residue molar ellipticity units [θ] (deg·cm^2^·dmol^–1^·residue^–1^) and smoothed using the Igor software. The percentage of helicity was calculated using the CDNN (version 1) deconvolution analysis software tool available on the Chirascan spectropolarimeter.

### Crystallization and Structural Determination

The synthesis and purification of HR1 were performed as previously reported.^24^ The amino acid sequence of HR1, i.e., RSV F(159–209), was Ac-HLEGEVNKIKSALLSTNKAVVSLSNGVSVLTSKVLDLKNYIDKQLLPIVNK-NH_2_. The HR1-**3/4m** and HR1-**4/4g** complexes (containing 10% DMSO) were purified to homogeneity by size exclusion chromatography using a Superdex 75 column (GE Healthcare), and the complex was concentrated to 6 mg/mL in 20 mM TRIS, pH 8, and 50 mM NaCl. Initial screening of crystallization conditions was carried out by the vapor diffusion method using a Mosquito nanoliter-dispensing system (TTP Labtech, Melbourn, United Kingdom) following established protocols.^44–47^ After optimization, the best diffracting crystals grew in 15% PEG 6K with 50 mM KCl and 10 mM MgCl_2_ and in 20% PEG 3350 with 200 mM NH4I with the HR1–**3/4m** and HR1–**4/4g** complexes, respectively. Crystals were flash-frozen by immersion into a cryo-protectant containing the crystallization solution supplemented with 5% (v/v) glycerol, 5% (v/v) MPD, and 5% (v/v) PEG400, or 15% ethylene glycol for the HR1-**3/4m** and HR1-**4/4g** complexes, respectively, followed by rapid transfer into liquid nitrogen.

X-ray data collection was carried out at SOLEIL on Proxima-2 and Proxima-1 beamlines. The crystals belong to the cubic space group *I*2_1_3 and diffracted to 1.6 Å resolution or to rhombohedral H32 and diffracted to 1.3 Å for the HR1–**3/4m** and HR1–**4/4g** complexes, respectively (Table S3). Data were processed, scaled, and reduced with autoPROC and Staraniso, using XDS^46,48^ and AIMLESS from the CCP4 suite.^49^ The structure was determined by molecular replacement using Phaser^50,51^ and PDB entry 1G2C^8^ as the search model. The crystal asymmetric units include a single protomer of the HR1–**3/4m** or HR1–**4/4g** heterodimer that crystallized along a crystallographic 3-fold axis, generating the HR1–**3/4m** six-helix bundle (6HB). Staple 1 and staple 2 were built using nonstandard amino acids (Fmoc)-S-2-(4-pentenyl)­alanine­(S5) and (Fmoc)-R-2-(4-pentenyl)­alanine (R5), corresponding to the L and R isomers of the resulting 2-methyl-norleucine (CCP4 nonstandard amino acids 2JN and MK8, respectively) after the Grubbs metathesis reaction, and introducing a planar double bond between the final carbon atoms of their side chain. The models which included residues 159–203 of the HR1 helix and the full **3/4m** double-stapled peptide or residues 159–206 of the HR1 helix and 497–514 of the **4/4g** double-stapled peptide were built by combining real space model building in Coot^52^ with reciprocal space refinement with BUSTER/TNT^53^ and validated with MolProbity.^54^

### rHRSV-mCherry Inhibition Assay

HEp-2 cells were seeded at 5 × 10^4^ cells per well in a 96-well plate the day before the infection. Peptides were 2-fold serially diluted in DMSO (11 dilutions), then further diluted in EMEM medium without phenol red, and preincubated for 15–20 min with rHRSV-mCherry at a final dilution of 0.2 MOI.^23^ The cells were incubated with virus/peptide mixtures for a period of 2 h. The medium was then replaced by EMEM media without phenol red containing 2% fetal bovine serum and the same concentration of peptide that was used during the infection step. Plates were incubated for 48 h at 37 °C, and the mCherry fluorescence was measured using a spectrofluorometer (Tecan Infinite M200PRO) with excitation and emission wavelengths of 580 and 620 nm, respectively. Relative fluorescence was normalized by the fluorescence of untreated infected cells. Noninfected HEp-2 cells were used as standards for fluorescence background levels. Each experiment was performed in duplicate and repeated at least twice.

### SmallAir Infection Assays

The SmallAir tissue culture model (24 well plate format) was developed by Epithelix (Geneva, Switzerland) from distal lung epithelial cells, as previously described.^55^ This model was cultured at the air–liquid interface (https://www.epithelix.com/products/) at 37 °C according to manufacturer’s instruction in the provided culture medium. Tissues were washed with 300 μL of PBS 45 min prior to infection. PBS was removed, and the virus (1 × 10^4^ pfu) was added to each well with or without 10 μM of peptide **3/4m** in 100 μL of SmallAir culture medium. Following 4 h of incubation at 37 °C, each tissue was washed 3 times with 200 μL of culture medium. The third wash was incubated with tissues for 20 min at 37 °C and then frozen at −80 °C. This aliquot was then used to quantify viral RNA by RT-PCR.^36^ The procedure was repeated every 24 h, followed by incubation of the tissues with 500 μL of culture medium.

### LC–MS/MS Analytic Methods

Triple quadrupole (QQQ) LC–MS/MS experiments were performed on an Agilent Technologies 6460 Triple Quad LC/MS, model G6460C, using an Acquity UPLC column BEH C18, 130 Å, 1.7 μM, 2.1 mm × 50 mm. These methods were used for the enzymatic, mouse plasma, and pharmacokinetic assays. The QQQ method that was developed involved Q1) an electrospray ionization step, Q2) a collision-induced dissociation step,^56^ Q3) a fragment selection step, and finally, multiple reaction monitoring following the QQQ steps.^57^ The software used to treat the data was Mass Hunter Workstation. The general chromatographic conditions were an injection volume of 5 μL, a column temperature of 35 °C, and a post-time acquisition of 2.00 min, but the LC gradient conditions were optimized for each peptide. The QQQ method was developed for each peptide as follows: peptides were injected at a concentration of approximately 1 μM. In Q1, the *MS2 Scan* mode was used to identify intact masses in a window range of 500 to 2000 Da. M + 2H^+^/2 and M + 3H^+^/3 species were observed for each peptide. In Q2, the *Product Ion* mode was selected, and the fragmenter’s fragment voltage and collision energy were varied to fragment the parent species. The parameters of fragmentation and the source were optimized automatically using the applications “Optimizer” and “Source Optimizer”, respectively. This process was repeated iteratively until the optimum conditions were reached. Only the most intense, reproducible fragments corresponding to the predicted theoretical fragments were selected for the Q3 fragment selection step.

### Enzymatic Stability Assay

5 μL of a 1.95 mM DMSO peptide stock solution was diluted 20-fold in a mixture of water/acetonitrile (5:3) to a final concentration of 97.5 μM. Trypsin (20 μg) was reconstituted by dissolving it in 40 μL of 50 mM acetic acid and 10 μL aliquots were stored at −80 °C (stable for two months). Chymotrypsin (25 μg) was reconstituted by dissolving it in 50 μL of 1 mM HCl, and 10 μL aliquots were stored at −80 °C (stable for two months). 10 μL of peptide at 97.5 μM was mixed with one aliquot of reconstituted trypsin and 980 μL of 100 mM ammonium bicarbonate, pH 8.0. 10 μL of peptide at 97.5 μM was mixed with one aliquot of reconstituted chymotrypsin and 980 μL of Tris-HCl, pH 8.0 , with 10 mM CaCl_2_. Following gentle mixing for 10 s, the reaction mixtures were immediately transferred into an HPLC vial. Aliquots were taken every 10 min for 1 h for further analysis. Peptides were extracted as follows: 50 μL of the plasma was mixed with 100 μL of acetonitrile (J.T. Baker, Cat. 9821) and vortexed for 40 s, and the mixture was allowed to settle for 20 min at RT. Following centrifugation of the suspension at 5000 rpm for 10 min, 50 μL of the supernatant containing the intact remaining peptide was quantified using the LC–MS analytical methods developed for each peptide.

### *In Vitro* Stability Assays in Mouse Plasma

Stock solutions of peptides **4bb**, **3/4m**, **3/4i**, and **4/4g** in DMSO (1.95 mM) were diluted in water/acetonitrile (5:3) to a final concentration of 250 μM, or 125 μM for **4/4g**. 2 μL of each peptide was then incubated with 48 μL of CD-1 mouse plasma (Innovative Research, Cat. IGMSCD1PLAK2E, Lot. 35365), and the mixtures were incubated at 37 °C at 200 rpm. Samples were taken at different time points (0, 0.25, 0.5, 1, 2 , 4, 6, 12, and 24 h) for further analysis, and the intact remaining peptide was quantified using the extraction and LC/MS–MS analytic method developed for each peptide as described above.

### Pharmacokinetic Studies

The TFA salts of peptides were exchanged to acetate salts using an acetate Dowex column as previously described.^58^ Peptides were dissolved in DMSO (1.95 mM) and freshly diluted with PBS, pH 7.4, to a final concentration of 98 or 293 μM for intravenous or intranasal administration, respectively. 200 μL (or 25 μL) of the resulting peptide solution was injected intravenously (or intranasally) to 8 week-old female Balb/cJRj mice (Janvier Laboratories, Le Genest-Saint-Isle, France). Following anesthesia of the mice with 1–2% isoflurane, approximately 100 μL of blood samples were retro-orbitally taken at 5 min, 15 min, 30 min, 60 min, 4 h, and 24 h time points after peptide injection. Three mice per peptide and per time point were administered. Plasma samples were then prepared with 250 UI/mL sodium heparin and subjected to a 15-minute centrifugation step at 1 500 g at 20 °C (no brake was used). Plasmas were stored at −80 °C. Perfused lungs were removed 1 h after peptide administration and frozen in hexane chilled with acetone/dry ice for storage. Lungs were frozen in liquid nitrogen, weighed, and crushed with a mortar. Lung samples were then resuspended in 50 μL PBS pH 7.4 and 150 μL acetonitrile. The resulting mixture was briefly vortexed and sonicated for 10 min, vortexed again for 30 min, and centrifuged for 10 min at 10 000 rpm at RT. Supernatants were filtered through a 0.22 μm filter into HPLC vials. The intact remaining peptide extracted from plasma and lungs was quantified by the LC/MS–MS analytic method developed for each peptide as described above.

### Biodistribution Studies

17 mg of peptide **3/4i** containing a beta-alanine residue at the N-terminus was dissolved in 900 μL of DMF and mixed with 7 μL of *N*,*N*-diisopropylethylamine (DIEA) 1 M in DMF (1 equiv) and 100 μL of the sulfo-cyanine 7 NHS ester 59.2 mM in DMSO (Lumiprobe, Cat. 25320). The reaction mixture was mixed at RT for 3 h and monitored by UHPLC-MS, and 3.5 μL of DIEA 1 M was added at 3 and 21 h. The reaction was stopped at 22 h, and the product was purified by HPLC semipreparative. 50 μL of peptide dissolved in DMSO/PBS pH 7.4 (5:95) at a concentration of 98 μM was administered intranasally to 8-week-old female Balb/c mice, and nonperfused lungs were removed 5 min after peptide administration. *In vivo* imaging was performed by fluorescence molecular tomography using FMT 1500 (PerkinElmer, France).

### RSV Infection of Mice and Intranasal Treatment

Female Balb/c mice were purchased from Janvier Laboratories (Le Genest-Saint-Isle, France). All mice were group-housed 5 per polypropylene cage in a standard temperature- and humidity-controlled biosafety laboratory 2 animal facility with a 12 h light–dark rhythm, unlimited access to food and water and enrichments (nests). Cages, food, enrichment, and water were sterilized before use. Mice at 8 weeks of age (*n* = 10 per group) were anesthetized with a mixture of ketamine and xylazine (1 and 0.2 mg per mouse, respectively) and treated intranasally (IN) with 50 μL of peptide 3/4i at 200 μM. Of note, peptide **3/4i** in DMSO was first diluted to 2 mM in 20 mM carbonate buffer and further diluted to 200 μM in PBS (0.8% DMSO), or the corresponding buffer for the control group. Ten minutes later, mice were infected intranasally with 60 μL of rHRSV-Luc (8 × 10^4^ p.f.u.). Mice were treated at 1-, 2-, and 3-day postinfection (dpi), and luminescence measurements were performed at 2 and 4 dpi. Mice were observed daily and weighed to assess the potential toxicity of the treatment.

### *In Vivo* Luminescence Measurements

Mice were anesthetized at 2 and 4 dpi, and bioluminescence was measured 5 min following IN instillation of 50 μL D-luciferin (30 mg/mL, PerkinElmer ). Living Image software (version 4.0, Caliper Life Sciences) was used to measure luciferase activity. Bioluminescence signals were acquired with an exposure time of 1 min. Digital false color photon emission images of mice were generated and show the average radiance (p/s/cm²/sr). Photons were counted within three different regions of interest corresponding to the nose, the lungs, and the whole airway area. Signals are expressed as total normalized flux (p/s). All data were analyzed using GraphPad Prism software version 6.07. The statistical significance for all *in vivo* bioluminescence experiments was measured using the Mann and Whitney (Houston) test. All data are presented as the mean ± standard error of the mean (SEM) and the *p* values.

### Histological Analysis

Mice were sacrificed at 4 dpi; the chest cavity was opened, and the lungs were perfused intratracheally with 4% PFA in PBS. The lungs were then removed and immersed in 4% PFA for 12 h before being transferred to 70% ethanol. The lungs were embedded in paraffin; 5 μm sections were cut and stained with Hematoxylin-Eosin-Saffron (HES) and evaluated microscopically by a trained veterinary pathologist. Qualitative histological changes were described and when applicable were scored semiquantitatively using a three scale from 0 to 2 (0: none, 1: mild, 2: marked) to document histological lesions (interstitial pneumonia, bronchi and vascular changes, and inflammation).

## Supplementary Material





## Notes

Material and Methods; surface interaction of peptide 4 in complex with trimeric RSV HR1 (Figure S1); preparation of HR1–**3/4m** and HR1–**4/4g** complexes for X-ray crystallography studies (Figure S2); ventral views of RSV luciferase expression in mice (Figure S3); distribution plot of potency versus staple positions (Figure S4); ribbon diagram of the HR1–**3/4m**, HR1–**4/4g**, and HR1–**3/4i** complexes (Figure S5); pharmacokinetics of peptide **3/4i** after IN or IV injection (Figure S6); quantification of peptide 3/4i in mice lungs 1 h post-IN or IV administration (Figure S7); double-stapled peptide scan (Table S1); staple 2 scan of peptides containing K508R5/Q501S5 staple 1c (Table S2); X-ray data collection and refinement statistics (Table S3); peptide interactions (Table S4); half-life of peptides measured in a proteolytic assay (Table S5); pharmacokinetic profile of lead peptides in mice (Table S6); individual observations of RSV-infected mice lung samples (Table S7); UPLC-MS traces of peptides studied in vivo; UPLC-MS traces of peptides studied in vitro; the file type extensions are jpeg (PDF)
The peptide sequences listed in Tables S1 and S2 (Molecular Formula Strings) (CSV)

## ¶

Nadège Pidoux: SICPA SA, Av. de Florissant 41, 1008 Prilly, Switzerland. Clémentine Prompt: Bond Life Sciences Center, Room #447, 1201 Rollins St-Columbia, MO 65211, USA

## Abbreviations used:

α: alpha
Å: angstrom
CAN: acetonitrile
BOC: *tert*-butoxycarbonyl
EC_50_: half-maximal inhibitory concentration
CD: circular dichroism
dpi: days post-infection
DIC: *N,N’*-diisopropylcarbodiimide
DIEA: *N*,*N*-diisopropylethylamine
DMSO: dimethyl sulfoxide
EMEM: Eagle’s minimum essential media
F: fusion
F: fusion
Fmoc: 9-fluorenylmethoxycarbonyl
FMT: fluorescence molecular tomography
HAE: human airway epithelium
HES: hematoxylin-eosin-Saffran
h.p.i: hours post-infection
HPLC: high-performance liquid chromatography
HR: heptad repeat
HRMS: high-resolution mass spectrometry
IN: intranasal
IV: intravenous
IVIS: in vivo imaging system
JNJ: Johnson & Johnson
nM: nanomolar
LC: liquid chromatography
MOI: multiplicity of infection
MS: mass spectroscopy
NHS: *N*-hydroxy succinimide
NMP: *N*-methylpyrrolidone
pfu: plaque-forming units
PBS: phosphate-buffered saline
PDB: protein data bank
PEG: polyethylene glycol
PFA: paraformaldehyde
PK: pharmacokinetics
postF: postfusion
preF: prefusion
R5: *R*-pentenylalanine
sHRSV: recombinant human RSV
RNA: ribonucleic acid
RSV: respiratory syncytial virus
RT-PCR: reverse transcriptase polymerase chain reaction
SAR: structure–activity relationship
S5: *S*-pentenylalanine
μM: micromolar
TMC: Tibotec medicinal chemistry
TFA: trifluoroacetic acid
TIS: triisopropylsilane, Tris, Tris­(hydroxymethyl)­aminomethane
UHPLC: ultrahigh pressure liquid chromatography
UV: ultraviolet
WT: wild-type
