# Supplementary material for: Double-Stapled Peptide Scan Yields Potent Fusion Inhibitors of Respiratory Syncytial Virus
Source: J Med Chem. 2026 May 27;69(11):12910–24. doi: 10.1021/acs.jmedchem.5c02932 (PMC13266986; doi:10.1021/acs.jmedchem.5c02932)
Supplement: Supplementary file 1 [file jm5c02932_si_001.pdf]

Supporting Information

Double-stapled peptide scan yields potent fusion inhibitors of Respiratory Syncytial Virus

Nadège Gsponer[a]†, Logan Roh[a], Nancy Nicolet[a], Roger Marti[b], Adrien Le Rouzic[c], Clémentine Prompt[c]†, Jenna Fix[c], Stéphane Duquerroy[d], Félix Rey[d,e], Marie-Anne Rameix-Welti[f], Mathilde Keck[g], Peggy Barbe[g], Dominique Garcin[h], Geneviève Mottet-Osman[h], Thibaut Larcher[i], Marie Galloux[c*], Origène Nyanguile[a*]

*Origène Nyanguile : origene.nyanguile@hevs.ch

*Marie Galloux : marie.galloux@inrae.fr

[a]HES-SO Valais-Wallis, Institute of Life Sciences, rue de l'Industrie 19, 1950 Sion, Switzerland ; [b]Institute of Chemical Technology, Haute école d'ingénierie et d'architecture Fribourg, HES-SO University of Applied Sciences and Arts Western Switzerland, 1700 Fribourg, Switzerland ; [c]VIM, INRAE, Domaine de Vilvert, 78350 Jouy-en-Josas, France ; [d]Université Paris Saclay, Structural Virology Unit, Institut Pasteur, 28 rue du Dr Roux, 75015 Paris – France ; [e]Université Paris-Saclay, Faculté des Sciences, 91400 Orsay, France ; [f]M3P, Institut Pasteur, Université Paris-Saclay, Université de Versailles St. Quentin, Université Paris Cité, UMR 1173 (2I), INSERM, Centre National de Référence Virus des Infections Respiratoire (CNR VIR), Assistance Publique des Hôpitaux de Paris, Hôpital Ambroise Paré, 75015 Paris, France, [g]Université Paris-Saclay, CEA, INRAE, Département Médicaments et Technologies pour la Santé (DMTS), SIMoS, 91191 Gif-sur-Yvette, France ; [h]Department of Microbiology and Molecular Medicine, University of Geneva School of Medicine, CMU, 1211 Geneva, Switzerland; [i]INRAE, UMR 703 APEX, Oniris, 44307 Nantes, France.

Contents

Contents S2

Figures S3

Figure S1. Surface interaction of peptide 4 in complex with trimeric RSV HR1........ S3

Figure S2. Preparation of HR1-3/4m, -4/4g complexes for X-ray crystallography studies. S3

Figure S3. Ventral views of luciferase expression in mice infected with rHRSV Luc........ S4

Figure S4. Distribution plot of potency versus staple positions. S4

Figure S5. .... S5

Figure S6. Pharmacokinetics of peptide 3/4i after IN or IV injection. S6

Figure S7. Quantification of peptide 3/4i in mice lungs 1 hour post IN or IV administration........ S6

Tables........ S7

Table S2. Staple 2 scan of peptides containing K508R5/Q501S5 staple 1. .... S8

Table S3. X-ray data collection and refinement statistics........ S9

Table S4. Peptide interactions........ S10

Table S5. Half-life of peptides measured in proteolytic assay........ S11

Table S6. Pharmacokinetic Profile of lead peptides in mice........ S12

Table S7. Individual histological observations of RSV-infected mice lung samples........ S12

UPLC-MS traces of peptides studied *in vivo* S13

UPLC-MS spectra of peptides studied *in vitro* S17

REFERENCES S51

Figures

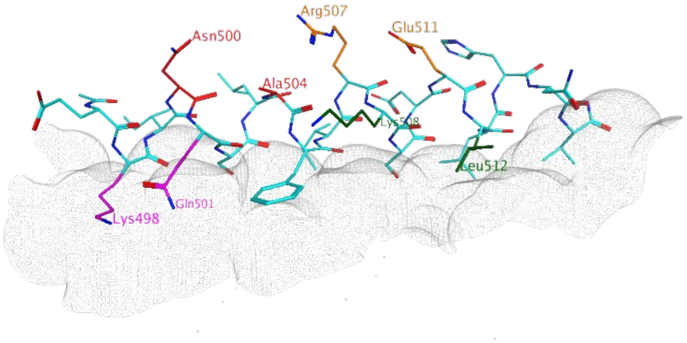

Figure S1. Surface interaction of peptide 4 in complex with trimeric RSV HR1. Peptide 4 is color coded in cyan and the HR1 interaction surface in a light gray grid. The amino acids that were replaced by the olefinic non-natural amino acids required for stapling in peptides 4/4g, 3/4i and 3/4m are color coded by pairs in magenta, red, gold and green. This figure was generated with molecular operating environment (MOE) developed by Chemical Computing ULC.

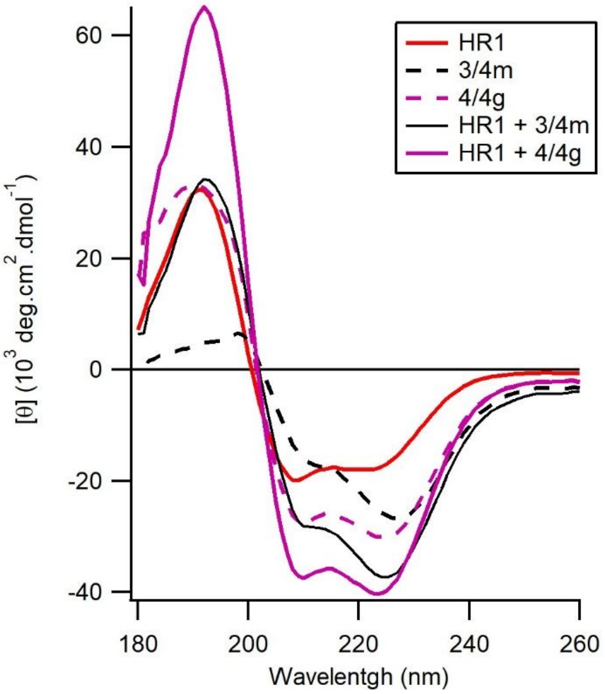

Figure S2. Preparation of HR1-3/4m, -4/4g complexes for X-ray crystallography studies. Far UV CD spectra of peptide **3/4m**, **4/4g**, HR1, HR1-**3/4m** and HR1-**4/4g** complexes. The samples were prepared at a concentration of 50 µM in 10 mM phosphate buffer pH 7. Data are reported as mean residue ellipticity θ.

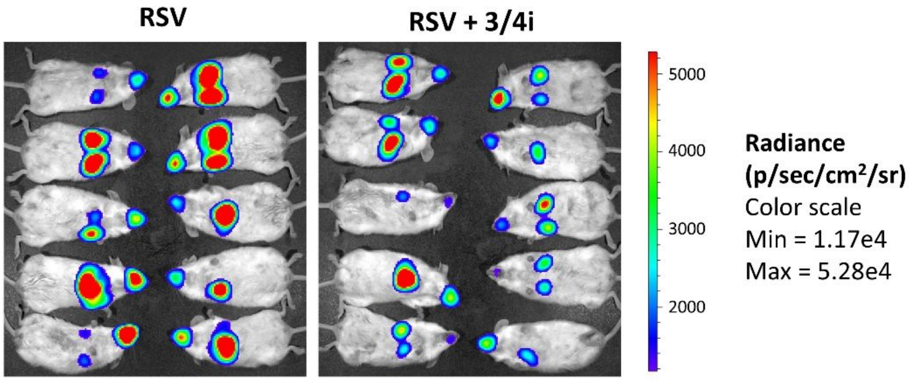

Figure S3. Ventral views of luciferase expression in mice infected with rHRSV Luc. Luciferase activities were quantified for each mouse using Living Image software. The scale on the right indicates the average radiance. Luciferase activity is expressed as photons per second (p s-1).

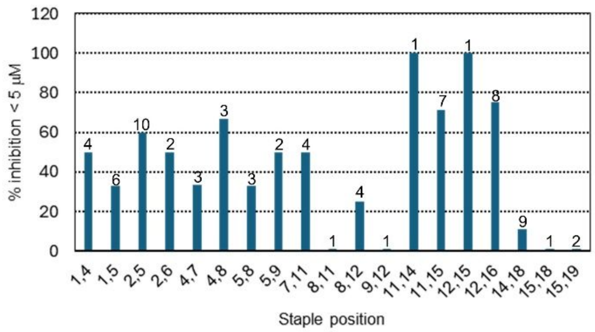

Figure S4. Distribution plot of potency versus staple positions. The x-axis shows the staple positions that were used in this study. The number of peptides containing these staples is shown above each corresponding bar. The y-axis represents the percentage of each staple yielding peptides with EC_{50} values lower than 5 μM.

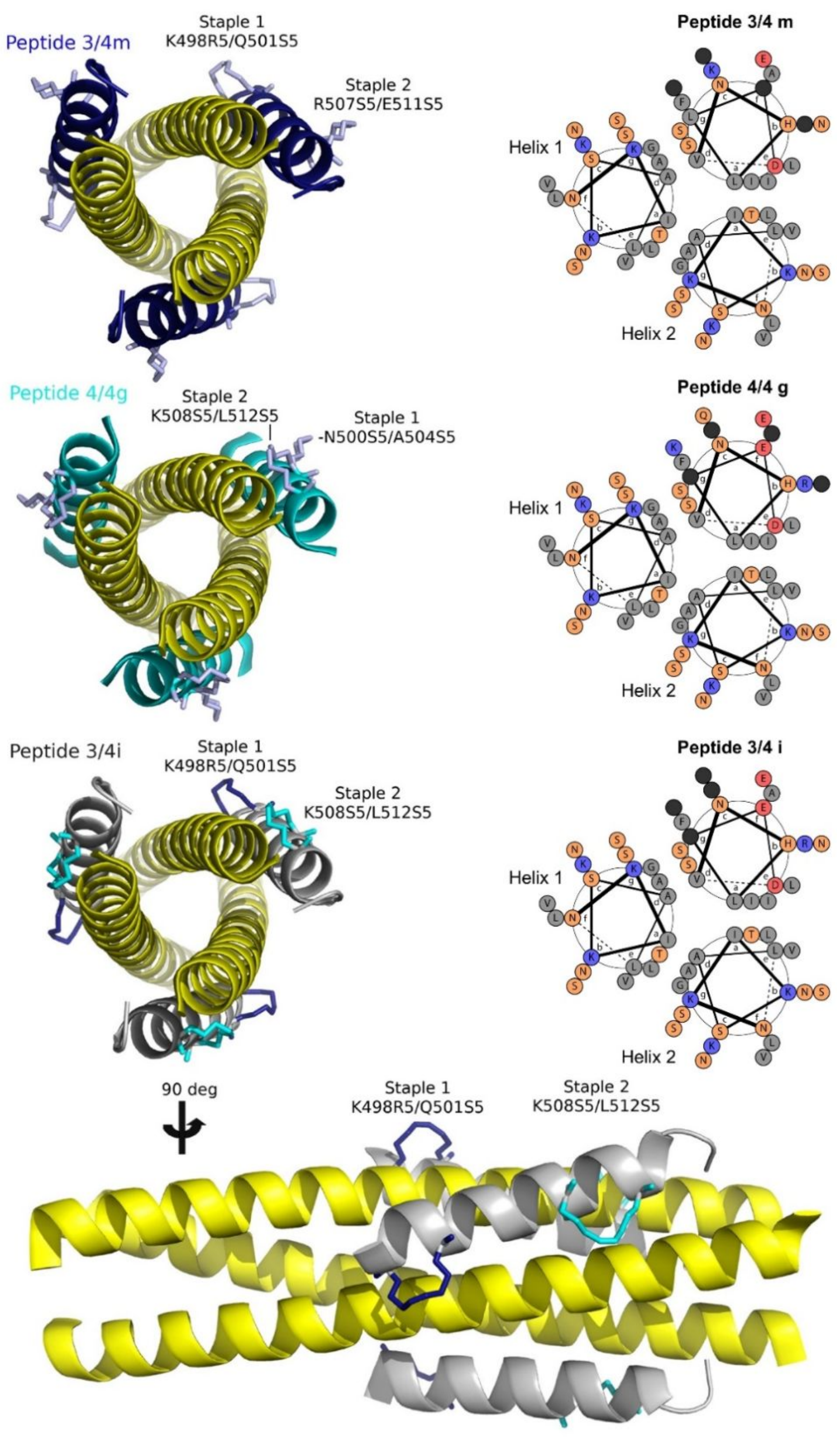

Figure S5. Ribbon diagram of the HR1-3/4m, HR1-4/4g and HR1-3/4i complexes. Left panel, rotated 90deg. relative to the view shown in Figure 2. The HR1 helices are shown in yellow, and the **3/4m, 4/4g and 3/4i** peptides in dark blue, cyan and gray, respectively. Staples 1 and 2 are shown in a tubular structure in light blue for peptides 3/4m and 4/4g or in dark blue and cyan for 3/4i peptide according to the structure used to build this model. Right panel, α-helical wheel representations depicting the interactions of the corresponding peptide with helix 1 and helix 2 of trimeric HR1. The (a) and (d) positions of peptide 3/4m, 4/4g and 3/4i interact with the helix 2 and helix 1 of HR1 trimer, respectively. The crosslinking unnatural amino acids are color coded in black. The alpha helical wheel diagrams were generated with DrawCoil 1.0 (https://grigoryanlab.org/drawcoil/). Bottom panel, model of the HR1-3/4i complex as drawn in figure 2 left. The HR1 helices are shown in yellow, and the **3/4i** peptide in gray. Staples 1 and 2 of **3/4i**, which were modeled from **3/4m** and **4/4g**, respectively, are shown in a tubular structure and color coded in dark blue and cyan, consistent with the color used for peptide **3/4m** and **4/4g** above.

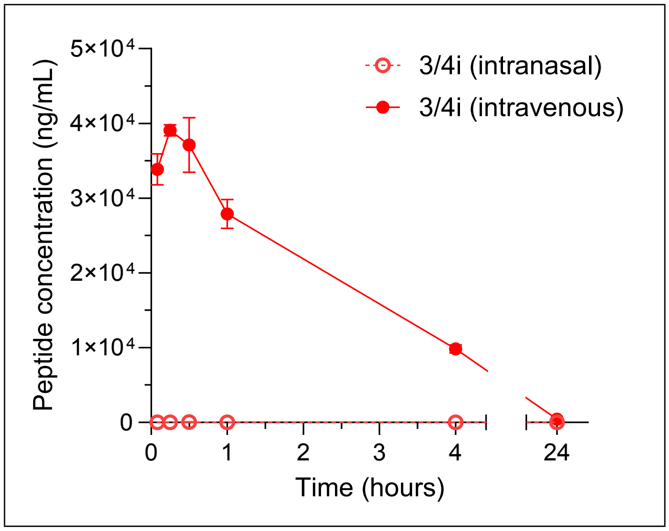

Figure S6. Pharmacokinetics of peptide 3/4i after IN or IV injection. Plasma concentration of peptide **3/4i** at different time points.

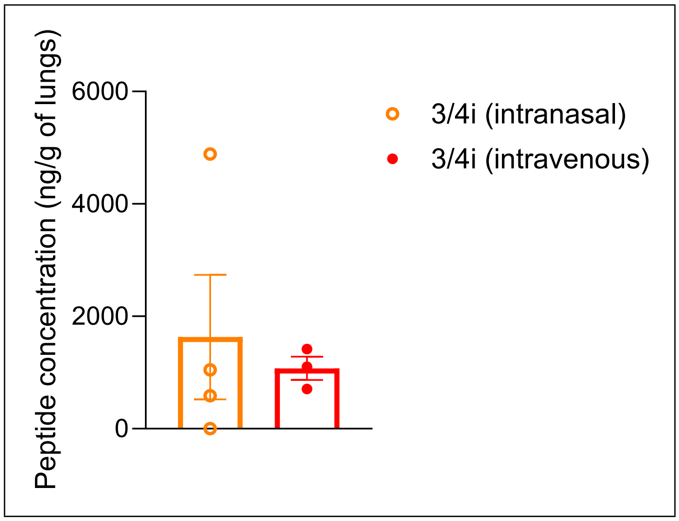

Figure S7. Quantification of peptide 3/4i in mice lungs 1 hour post IN or IV administration

Tables

TABLE S1. Double stapled peptide scan. Amino-acid sequences with residues critical for the binding to HR1 color coded in red and non-essential residues in black; α-helical content, and EC_{50} values of double stapled peptides derived from peptide **4** (F (497-516))

Peptide	497			500					505					510					515		% Helicity	EC_{50} (μM)
4	E	K	**I**	N	Q	**S**	L	A	F	**I**	R	K	**S**	D	E	L	**L**	H	N	**V**	6.6	> 50
										i; i+4/ i; i+4												
	**1**	**2**	3	4	**5**	6	7	**8**	9	10	**11**	**12**	13	14	**15**	**16**	17	18	19	20		
4bb	-	-	-	X	-	-	-	X	-	-	X	-	-	-	X	-	-	-	-	-		0.74±0.27
4/4a	X	-	-	-	X	-	-	-	-	-	-	-	-	-	X	-	-	-	X	-	72.2	> 33.00
4/4b	-	-	-	-	-	-	-	X	-	-	-	X	-	X	-	-	-	X	-	-	32.3	186.69±117
4/4c	-	-	-	-	-	-	X	-	-	-	X	-	-	X	-	-	-	X	-	-	29.6	6.52±0.58
4/4d	-	-	-	X	-	-	-	X	-	-	-	-	-	X	-	-	-	X	-	-	27.9	5.68±0.65
4/4e	X	-	-	-	X	-	-	-	-	-	-	-	-	X	-	-	-	X	-	-	40.8	6.77±6.51
4/4f	-	-	-	-	X	-	-	-	X	-	-	X	-	-	-	X	-	-	-	-	57.1	0.95±0.66
**4/4g**	**-**	**-**	**-**	**X**	**-**	**-**	**-**	**X**	**-**	**-**	**-**	**X**	**-**	**-**	**-**	**X**	**-**	**-**	**-**	**-**	99.1	**0.12±0.14**
4/4h	-	X	-	-	-	X	-	-	-	-	-	X	-	-	-	X	-	-	-	-	ND	0.53±0.04
4/4i	X	-	-	-	X	-	-	-	-	-	-	X	-	-	-	X	-	-	-	-	58.6	0.36±0.21
4/4j	-	-	-	-	X	-	-	-	X	-	X	-	-	-	X	-	-	-	-	-	58.7	34.32±8.89
4/4k	-	X	-	-	-	X	-	-	-	-	X	-	-	-	X	-	-	-	-	-	92.7	54.63±68.70
4/4l	X	-	-	-	X	-	-	-	-	-	X	-	-	-	X	-	-	-	-	-	61.9	2.61±2.35
4/4m	X	-	-	-	X	-	-	X	-	-	-	X	-	-	-	-	-	-	-	-	51.9	9.13±6.94
4/4n	X	-	-	-	X	-	X	-	-	-	X	-	-	-	-	-	-	-	-	-	27.0	>33.00
										i; i+3/ i; i+4												
	1	2	3	4	5	6	7	8	9	10	11	12	13	14	15	16	17	18	19	20		
4ca	-	+	-	-	X	-	-	X	-	-	-	X	-	-	-	-	-	-	-	-		0.59±0.13
3/4a	-	+	-	-	X	-	-	-	-	-	-	-	-	-	X	-	-	-	X	-	52.0	8.89±3.21
3/4b	-	-	-	-	-	-	-	-	+	-	-	X	-	X	-	-	-	X	-	-	49.4	4.94±1.88
3/4c	-	-	-	-	-	-	-	+	-	-	X	-	-	X	-	-	-	X	-	-	36.9	33.95±10.6
3/4d	-	-	-	-	+	-	-	X	-	-	-	-	-	X	-	-	-	X	-	-	33.9	17.15±1.86
3/4e	-	-	-	+	-	-	X	-	-	-	-	-	-	X	-	-	-	X	-	-	43.2	5.87±0.83
3/4f	-	+	-	-	X	-	-	-	-	-	-	-	-	X	-	-	-	X	-	-	95.5	73.42±53.3
3/4g	-	-	-	-	+	-	-	X	-	-	-	X	-	-	-	X	-	-	-	-	38.8	19.69±10.2
3/4h	-	-	-	+	-	-	X	-	-	-	-	X	-	-	-	X	-	-	-	-	98.2	4.48±3.52
**3/4i**	**-**	**+**	**-**	**-**	**X**	**-**	**-**	**-**	**-**	**-**	**-**	**X**	**-**	**-**	**-**	**X**	**-**	**-**	**-**	**-**	65.1	**0.083±0.078**
3/4j	+	-	-	X	-	-	-	-	-	-	-	X	-	-	-	X	-	-	-	-	42.1	>33.00
3/4k	-	-	-	-	+	-	-	X	-	-	X	-	-	-	X	-	-	-	-	-	31.0	3.95±5.19
3/4l	-	-	-	+	-	-	X	-	-	-	X	-	-	-	X	-	-	-	-	-	61.6	10.92±5.19
**3/4m**	**-**	**+**	**-**	**-**	**X**	**-**	**-**	**-**	**-**	**-**	**X**	**-**	**-**	**-**	**X**	**-**	**-**	**-**	**-**	**-**	56.7	**0.053±0.041**
3/4n	+	-	-	X	-	-	-	-	-	-	X	-	-	-	X	-	-	-	-	-	33.9	2.61±1.79
3/4o	+	-	-	X	-	-	-	X	-	-	-	X	-	-	-	-	-	-	-	-	57.1	66.50±47.38
3/4p	-	+	-	-	X	-	X	-	-	-	X	-	-	-	-	-	-	-	-	-	28.0	1.88±0.46
3/4q	+	-	-	X	-	-	X	-	-	-	X	-	-	-	-	-	-	-	-	-	35.2	2.42±0.20

X, S-pentenylalanine; +, R-pentenylalanine; EC_{50}, half maximal inhibitory concentration

%Helicity = 100 x $[\theta]_{222}/[\theta]^{max}_{222}$, where $[\theta]^{max}_{222}$ = - 40'000 x (1- 2.5/number of amino acid residues)

Table S2. Staple 2 scan of peptides containing K508R5/Q501S5 staple 1.

Peptide	497			500				500					510					515			EC_{50} (μM)
4	E	K	I	N	Q	S	L	A	F	I	R	K	S	D	E	L	L	H	N	V	> 50
	1	2	3	4	5	6	7	8	9	10	11	12	13	14	15	16	17	18	19	20	
3/3a	-	+	-	-	X	-	-	-	-	-	-	-	-	-	-	+	-	-	X	-	15.0
3/3b	-	+	-	-	X	-	-	-	-	-	-	-	-	-	+	-	-	X	-	-	>33.0
3/3c	-	+	-	-	X	-	-	-	-	-	-	+	-	-	X	-	-	-	-	-	0.86±0.74
3/3d	-	+	-	-	X	-	-	-	-	-	+	-	-	X	-	-	-	-	-	-	1.71±2.65
3/3e	-	+	-	-	X	-	-	-	+	-	-	X	-	-	-	-	-	-	-	-	>33.0
3/3f	-	+	-	-	X	-	-	+	-	-	X	-	-	-	-	-	-	-	-	-	>33.0

X, S-pentenylalanine; +, R-pentenylalanine; EC_{50}, half maximal inhibitory concentration

Table S3. X-ray data collection and refinement statistics

Data collection and refinement statistics

PDB accession code	9RA5	29QJ
**Data collection**	PROXIMA-2A (2022-07-10)	PROXIMA-1 (2026-02-13)
Space group	I 21 3	H 3 2
Unit cell parameters		
a / b / c (Å)	78.32 / 78.32 / 78.32	41.08 / 41.08 / 200.84
α / β / γ (deg)	90 / 90 / 90	90 / 90 / 120
Resolution (Å)	20-1.625 (1.65-1.625)[a]	60-1.325 (1.432-1.325)
Total observations	410341 (31936)	240786 (11903)
Unique reflections	10226 (533)	12173 (609)
Completeness spherical (%)	100.0 (100.0)	77.3 (19.3)
Completeness ellipsoidal (%)	100.0 (100.0)	92.1 (65.4)
Redundancy	40.1 (41.2)	19.8 (19.5)
<I/σ>[b]	27.0 (0.8)	18.0 (1.2)
R_{merge} (%)	0.068 (6.572)	0.073 (2.258)
R_{meas} (%)	0.069 (6.653)	0.075 (2.319)
R_{pim}(%)	0.011 (1.035)	0.017 (0.522)
CC(1/2)	1.000 (0.298)	0.997 (0.660)
**Refinement**		
Resolution (Å)	15-1.62 (1.65-1.62)	15-1.325 (1.41-1.32)
Number of reflections	10211 (496)	12152 (406)
Number of Rfree reflections	496 (14)	612 (27)
Rfactor (%)	19.34 (31.43)	20.49 (27.47)
Rfree (%)	20.75 (32.58)	23.21 (32.67)
Number of protein atoms	468	481
Number of heterogen atoms (waters)	50 (36)	42 (47)
Root mean square deviations		
Bond lengths (Å)	0.010	0.012
Bond angles (º)	0.86	1.20
Ramachandran fav/out (%)	100/0.0	100/0
Rotamers outliers (%)	0.0	0.0

[a]Values in parentheses correspond to highest resolution shell.

Table S4. Peptide interactions

Heptad[a]	Residues	ASA[b]	Helix 1 BSA[b]	Helix 1 HB [d]	Helix 2 BSA[b]	Helix 2 HB [d]	Interaction strength
f	Glu497	161.77	0.00		0.00		
**g[c]**	**K498R5**	**116.06**	**76.64**		**0.00**		++++++++
**a**	**Ile 499**	**136.06**	**25.85**		**88.14**		+++++++++++
b	Asn500	107.06	0.00		0.00		
c	Q501S5	77.65	10.36		0.00		+
**d**	**Ser502**	**61.76**	**43.35**	+	**18.41**		++++++
e	Leu503	110.04	0.00		40.67		++++
f	Ala504	43.01	0.00		0.00		
**g**	**Phe505**	**125.50**	**79.70**	+	**0.00**		++++++++
**a**	**Ile506**	**102.17**	**30.07**		**66.12**		++++++++++
b	R507S5	88.58	0.00		0.00		
c	Lys508	96.99	5.50		0.00		+
**d**	**Ser509**	**52.13**	**32.83**	++	**19.31**	+	+++++
e	Asp510	83.83	0.00		34.57	+	+++
f	E511S5	104.37	0.25		0.00		
**g**	**Leu512**	**145.05**	**81.95**		**0.00**		++++++++
**a**	**Leu513**	**108.25**	**31.80**		**76.45**	+	+++++++++++
b	His514	148.85	0.00	+	6.39		+
c	Asn515	144.51	34.15		0.00		+++
**d**	**Val516**	**116.23**	**64.01**		**52.22**		++++++++++++
f	Glu497	127.64	0.00		0.00		
**g[c]**	**Lys498**	**154.23**	**81.75**	+	**0.00**		++++++++
**a**	**Ile499**	**146.76**	**29.57**		**81.59**		+++++++++++
b	N500S5	80.00	0.00		0.00		
c	Gln501	63.24	8.02		0.00		+
**d**	**Ser502**	**57.35**	**38.74**		**18.61**		++++++
e	Leu503	119.06	0.00		61.18		++++++
f	A504S5	92.68	0.00		0.00		
**g**	**Phe505**	**133.12**	**76.35**	+	**0.00**		++++++++
**a**	**Ile506**	**107.04**	**35.60**		**71.44**		+++++++++++
b	Arg507	167.38	0.00		0.00		
c	K508S5	93.56	15.24		0.00		++
**d**	**Ser509**	**42.74**	**29.22**	+	**13.52**	+	++++
e	Asp510	63.44	0.00		26.00	+	+++
f	Glu511	84.37	0.00		0.00		
**g**	**L512S5**	**142.74**	**66.12**		**0.00**		+++++++
**a**	**Leu513**	**138.51**	**45.54**		**92.97**		++++++++++++++
b	His514	186.01	7.26		19.45		+++
c	Asn515						
**d**	**Val516**						

[a]α-helical wheel heptad position of HR1 helix 1 or HR1 helix 2

[b]ASA, accessible surface area, and BSA were determined using PDBePISA (protein interaction surface analysis European bioinformatic Institute, EBI CH). The ASA and BSA values represent the interaction between residues of peptide 3/4m (top) and 4/4g (bottom) with HR1 helix 1 and HR1 helix 2

[c]The characters in bold are the residues of peptide at heptad position a, d and g, which make main hydrophobic contacts with trimeric HR1

[d] H-bonds between peptide and HR1 helices as determined by ChimeraX[1-2]

Table S5. Half-life of peptides measured in proteolytic assay

Peptide	Trypsin (min)	Chymotrypsine (min)	Mice serum (hrs)
4	4.91	4.36	1.25
3/4i	40.91	20.13	367.80
3/4m	18.15	108.30	302.76
4/4g	91.98	36.36	102.77
4bb	23.32	43.61	59.17

Table S6. Pharmacokinetic Profile of lead peptides in mice

	Peptide 3/4i	Peptide 3/4m	Peptide 4/4g
Elimination rate (1/h)	0.18	0.15	0.16
$T_{1/2}$ (h)	3.7	4.5	4.2
T_{max} (min)	15	15	5
C_{max} (nM)	16283	3597	3018
Area Under the Curve (AUC_{0-t}) (nM*h)	61032	8049	2411
Mean Residence Time (MRT_{0-t}) (h)	4.8	5.0	3.0
Volume of distribution	8.6	75.8	242.4
Clearance/F (h)	1.6	11.6	39.7

Table S7. Individual histological observations of RSV-infected mice lung samples.

Lesions were scored semi-quantitatively using a three level-scale from 0 to 2 (0: none, 1: mild, 2: marked).

Treatment	Mice #	Bronchial epithelium hyperplasia	Bronchial content	BALT	Exocytosis	Vasc./bronchial cuffing	Interest. Pneumonia
3/4i	1	0	1	0	0	0	0
3/4i	2	0	1	0	0	0	0
3/4i	3	0	2	0	0	0	0
3/4i	4	0	1	0	0	0	0
3/4i	5	0	1	0	0	0	0
3/4i	6	0	0	0	0	0	0
Vehicle	1	1	2	0	1	1	1
Vehicle	2	2	1	1	1	2	2
Vehicle	3	0	0	0	0	1	0
Vehicle	4	0	2	0	0	2	0
Vehicle	5	0	2	0	0	1	0
Vehicle	6	1	1	0	0	1	0

UPLC-MS traces of peptides studied *in vivo*

Peptide purity was determined by integration of the area under the curves of each peak observed in the chromatogram. The conditions used for UPLC were: Flow rate: 0.620 mL/min; Eluent A: H_2O + 0.03% TFA; Eluent B: ACN + 0.03% TFA; Gradient: 5% to 95% Eluant B. The conditions used for MS were: Masses: 300 to 1600 m/z; Ion mode: ESI; Fragmentor: 200 V; Polarity: positive.

Peptide 3/4i (96.92% purity)

A)

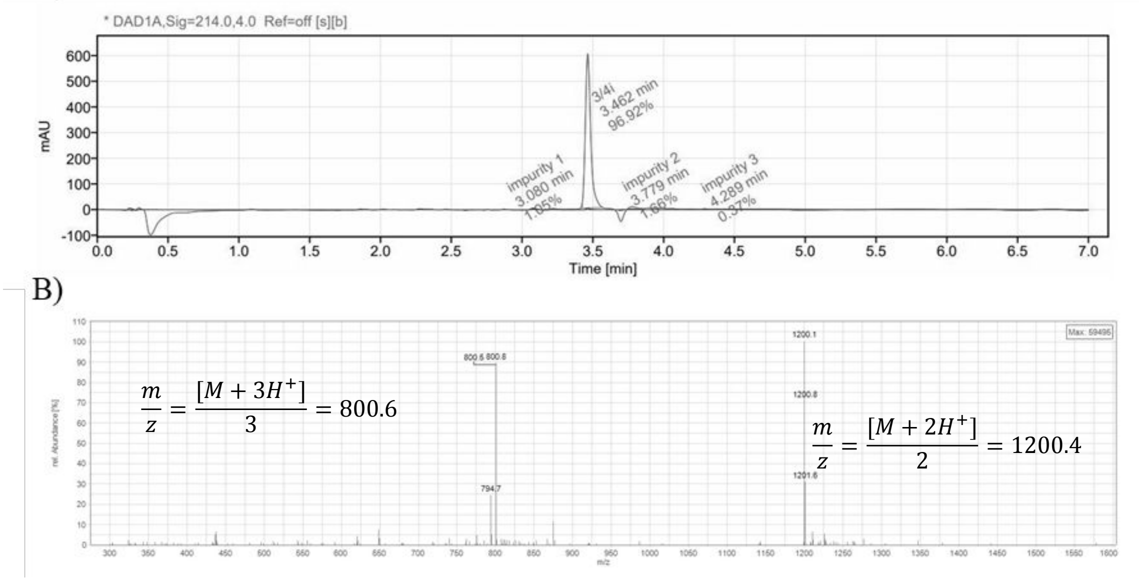

A) UPLC profile at 214 nm; B) ES-MS spectrum at 3.462 min. Expected MW = 2398.74 g/mol.

Peptide 3/4i-Scy7 (sulfo-cyanine 7, 96.17% purity)

A)

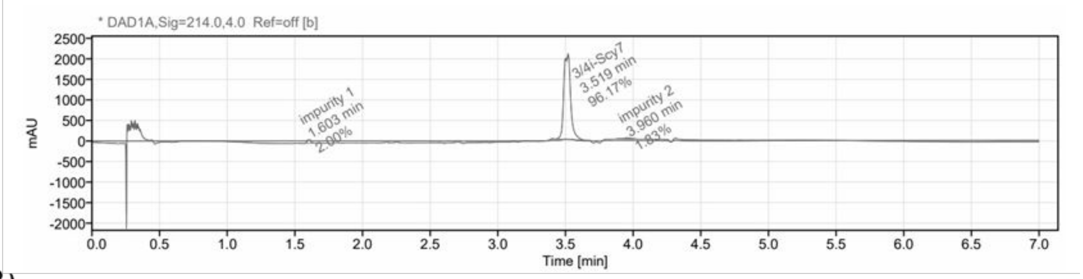

B)

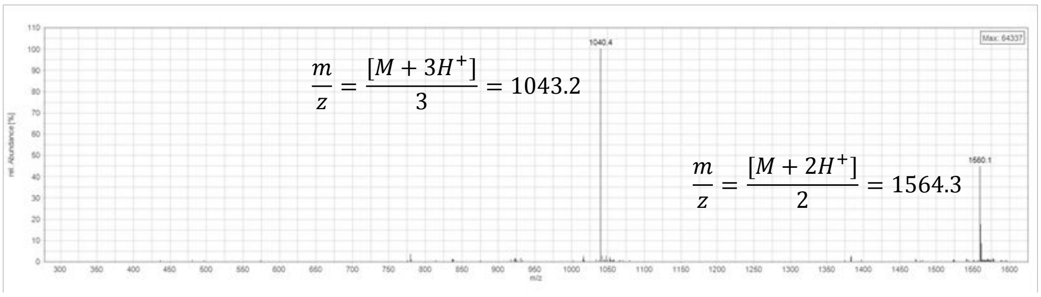

A) UV spectrum at 214 nm; B) ES-MS spectrum at 3.519 min. Expected MW = 3126.69 g/mol.

Peptide 3/4m (96.05% purity)

A)

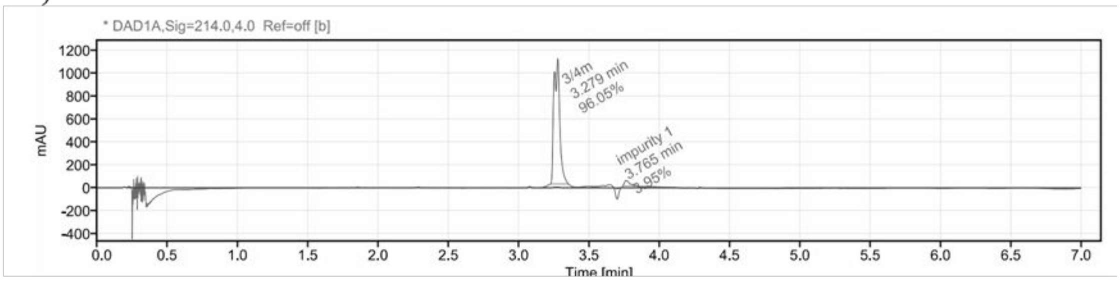

B)

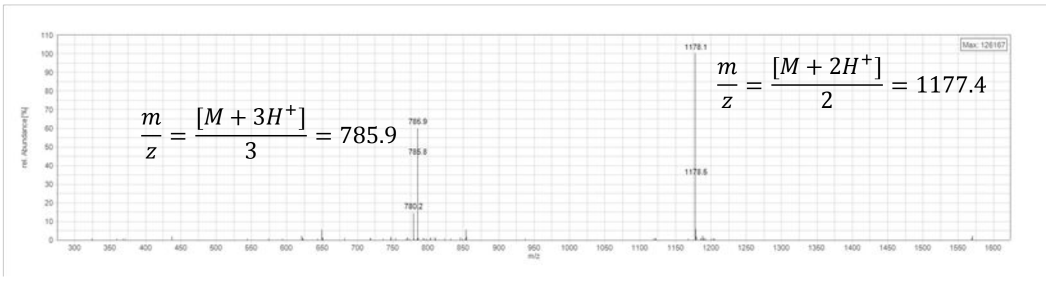

A) UV spectrum at 214 nm; B) ES-MS spectrum at 3.279 min. Expected MW = 2354.77 g/mol.

Peptide 4/4g (98.56% purity)

A)

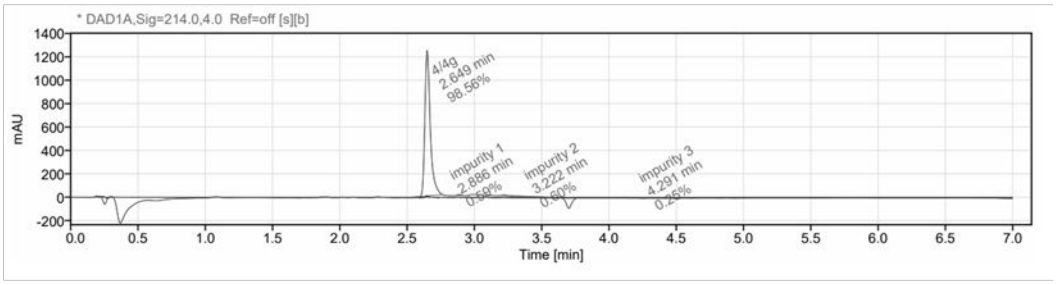

B)

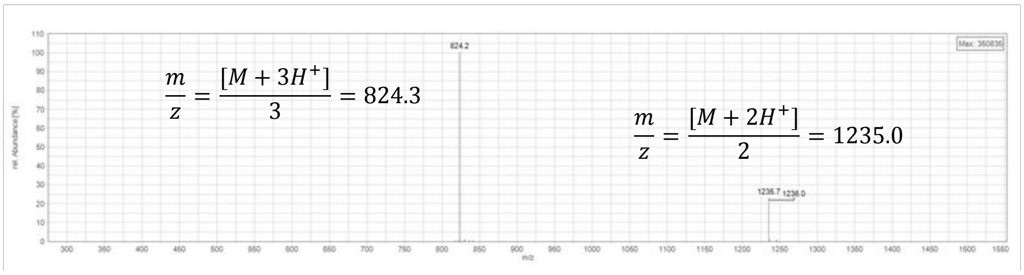

A) UV spectrum at 214 nm; B) ES-MS spectrum at 2.749 min. Expected MW = 2469.86 g/mol.

UPLC-MS spectra of peptides studied *in vitro*

Peptide purity was determined by integration of the area under the curves of each peak observed in the chromatogram. 1 µL of a 15mM stock solution in DMSO was diluted to 10 ml with ACN/H_2O 1:1 prior to injection. In some instances, two peaks with the same molecular weight were observed, corresponding to the syn- and anti-configuration of the staple alkene bond.

Peptide 3/3a (> 99% purity)

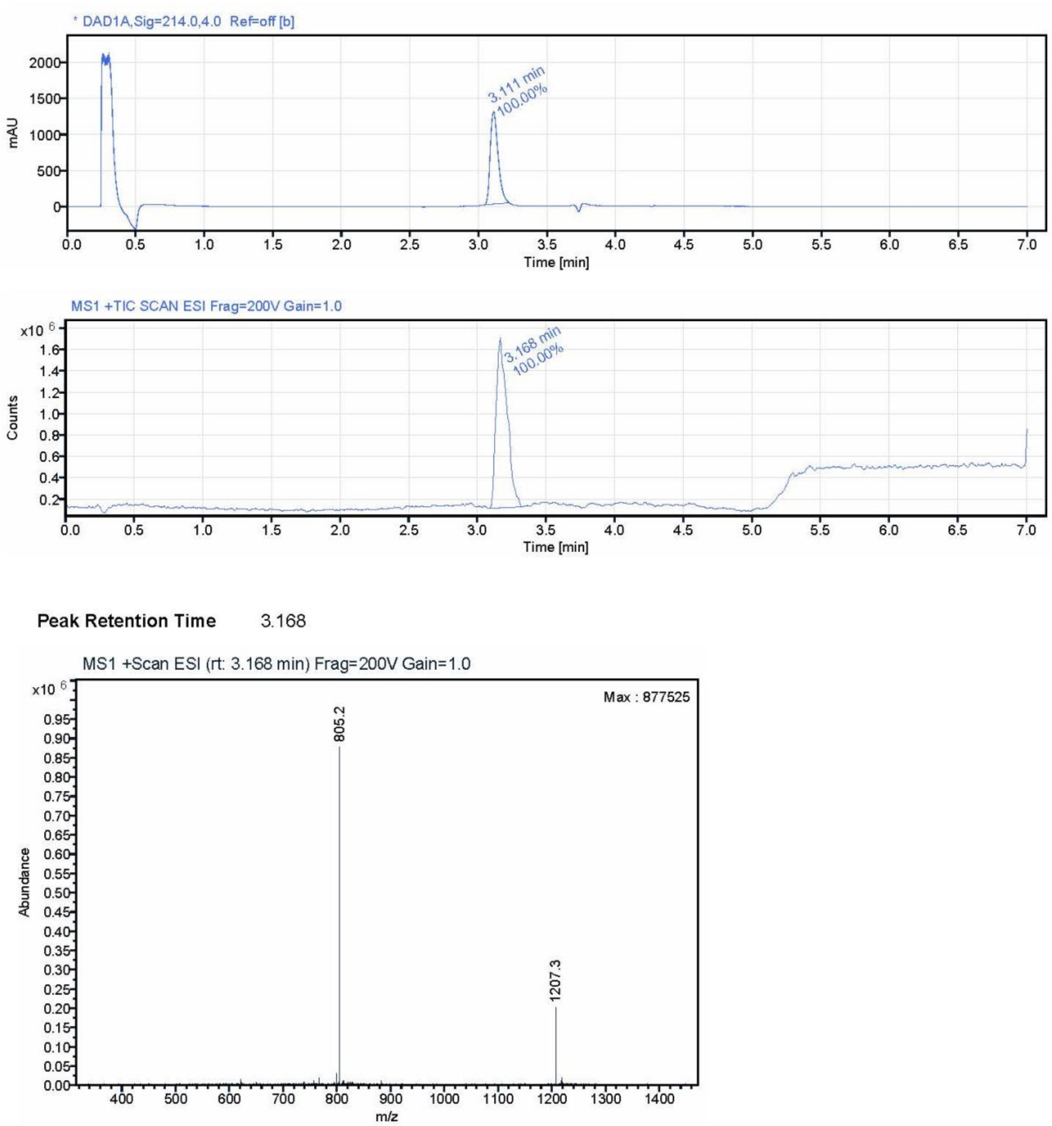

Expected MW = 2412.81 g/mol

Peptide 3/3b (> 99% purity)

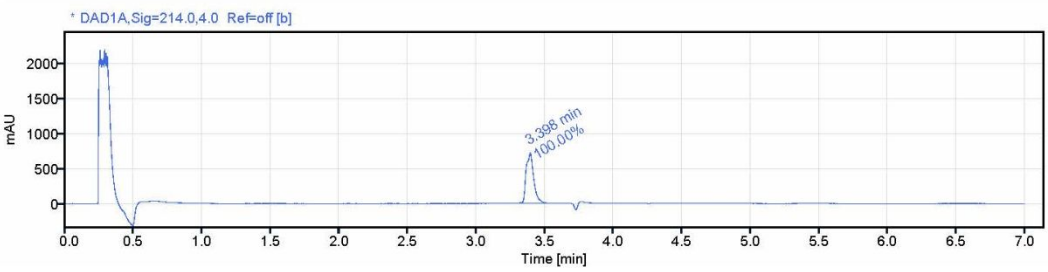

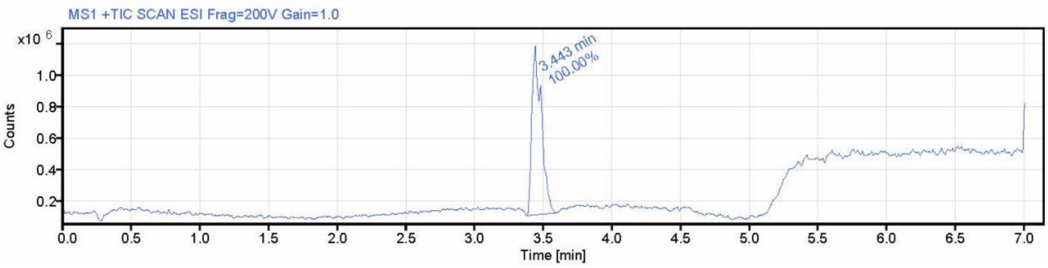

Peak Retention Time 3.443

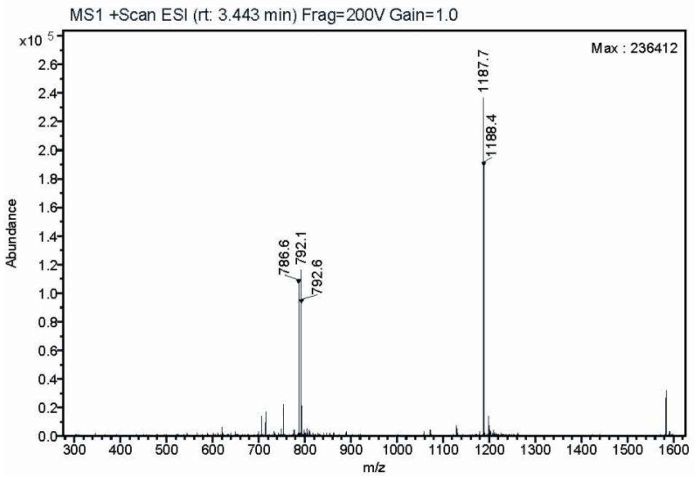

Expected MW = 2373.82 g/mol

Peptide 3/3c (> 99% purity)

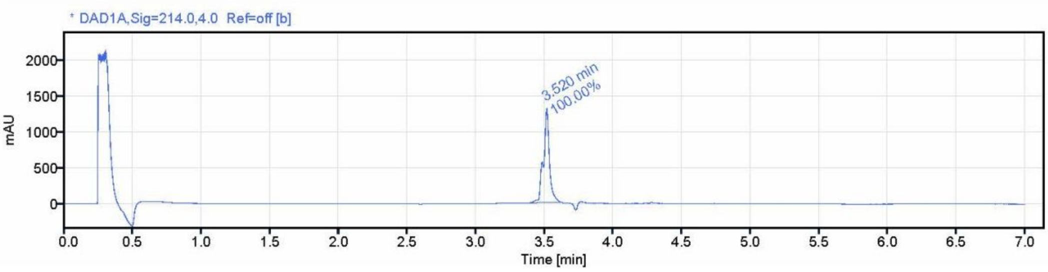

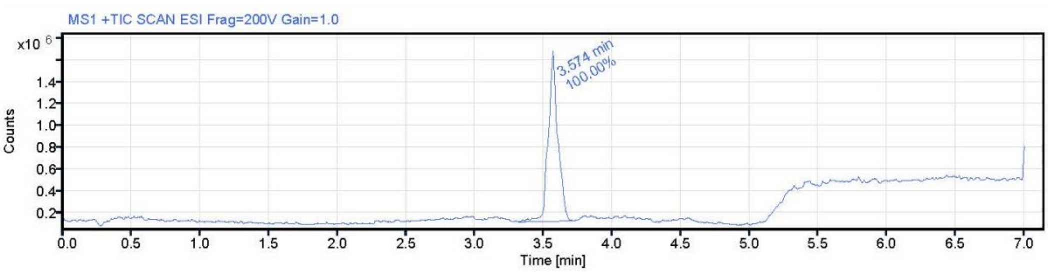

Peak Retention Time 3.574

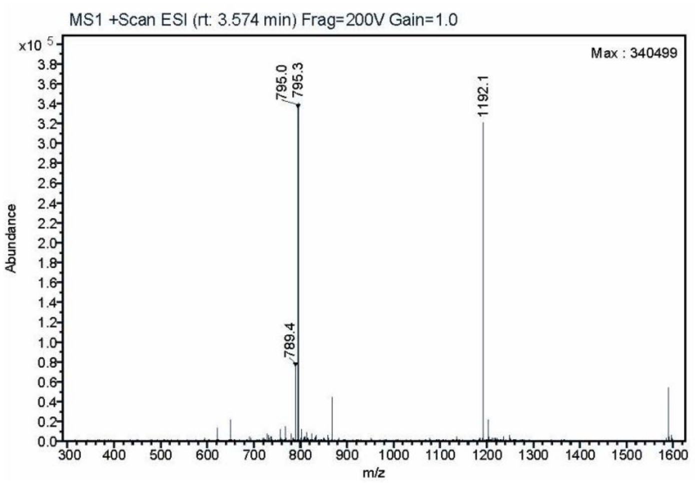

Expected MW = 2382.79 g/mol

Peptide 3/3d (> 99 % purity)

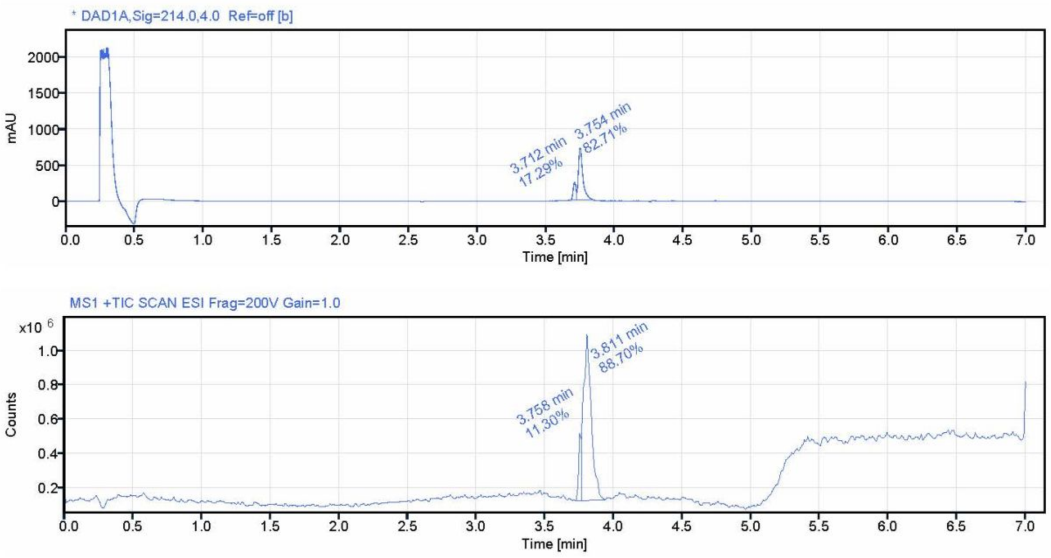

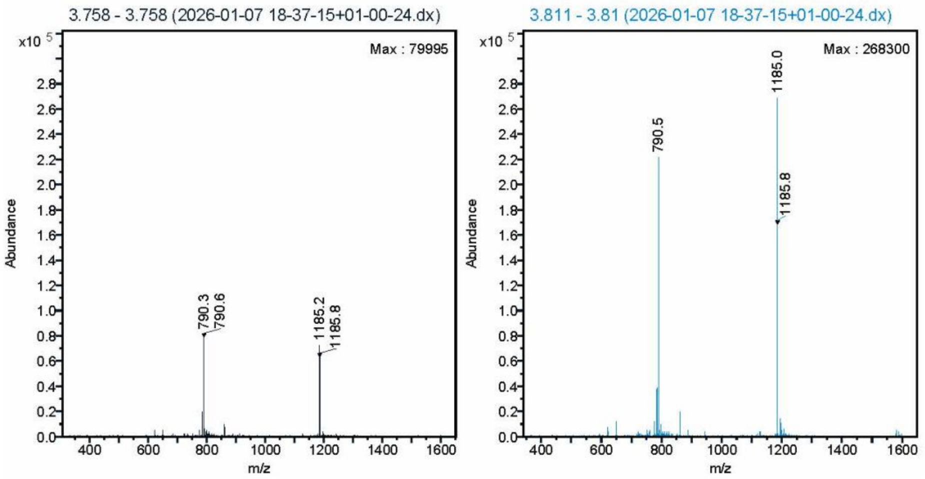

Expected MW = 2368.79 g/mol

Peptide 3/3e (> 99% purity)

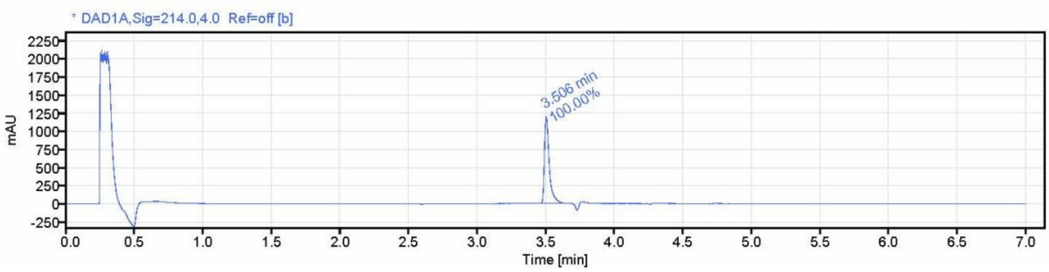

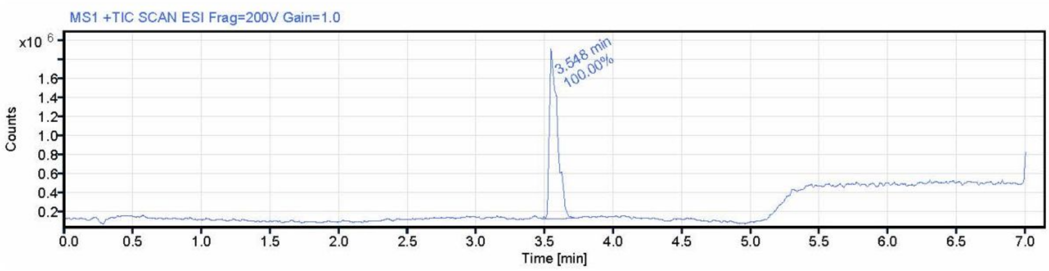

Peak Retention Time 3.548

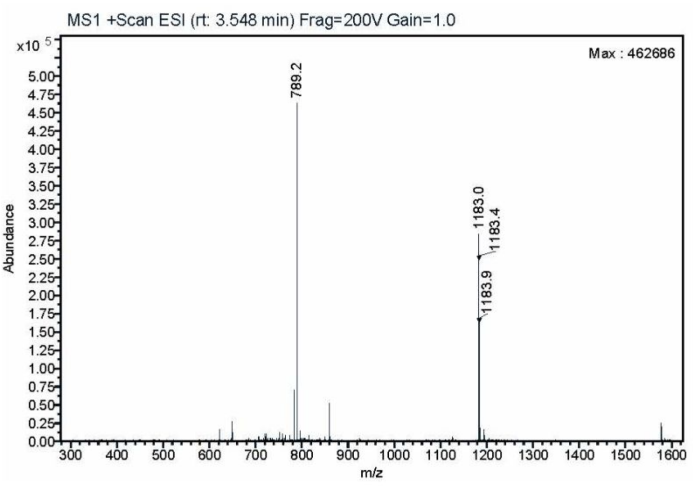

Expected MW = 2364.72 g/mol

Peptide 3/3f (> 99% purity)

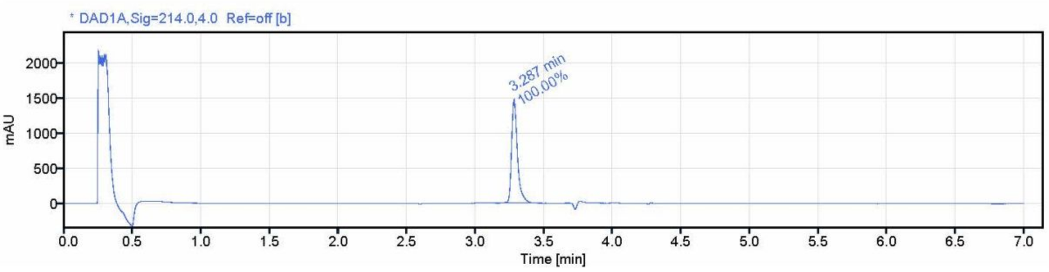

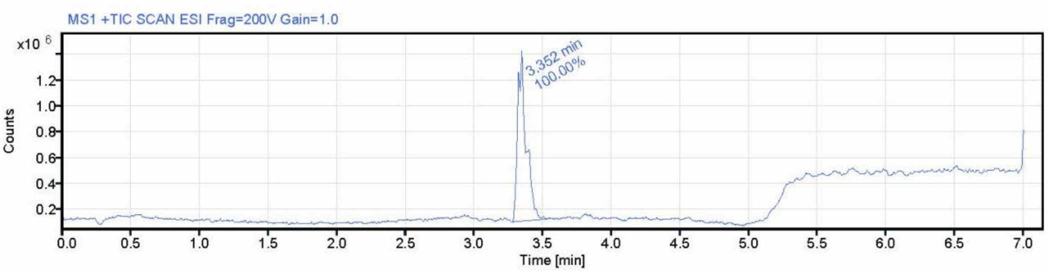

Peak Retention Time 3.352

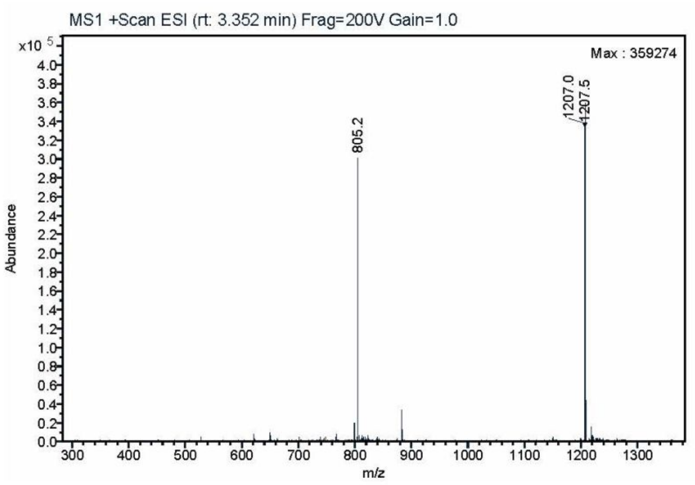

Expected MW = 2412.8 g/mol

Peptide 3/4a (> 99% purity)

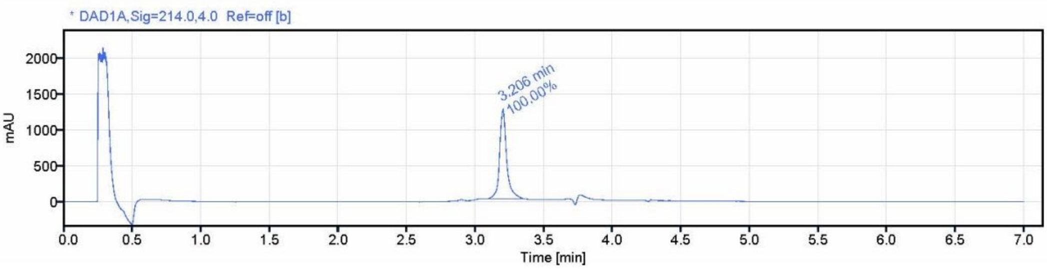

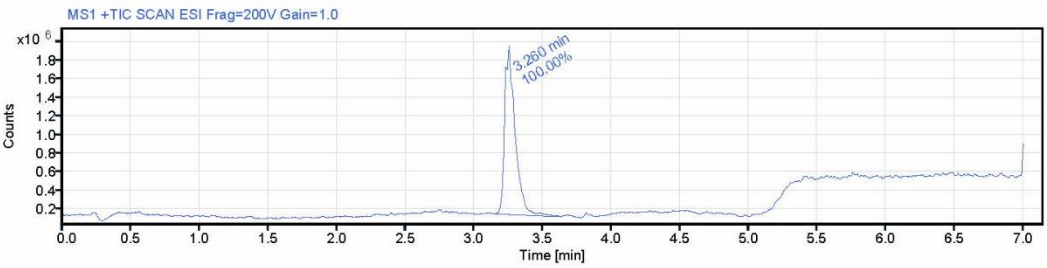

Peak Retention Time 3.260

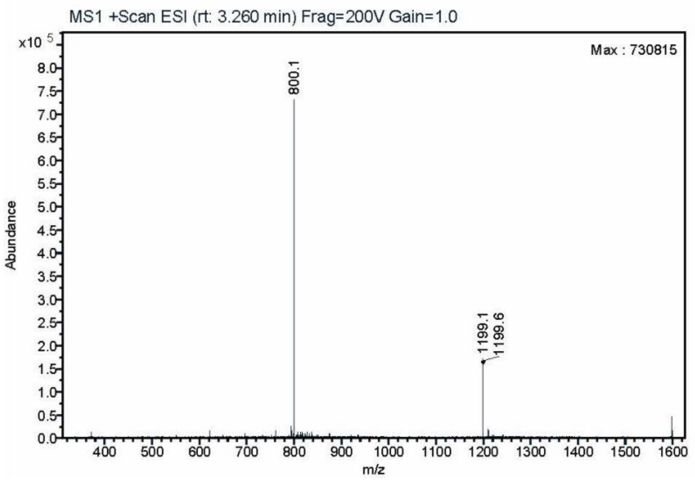

Expected MW = 2396.86 g/mol

Peptide 3/4b (> 99% purity)

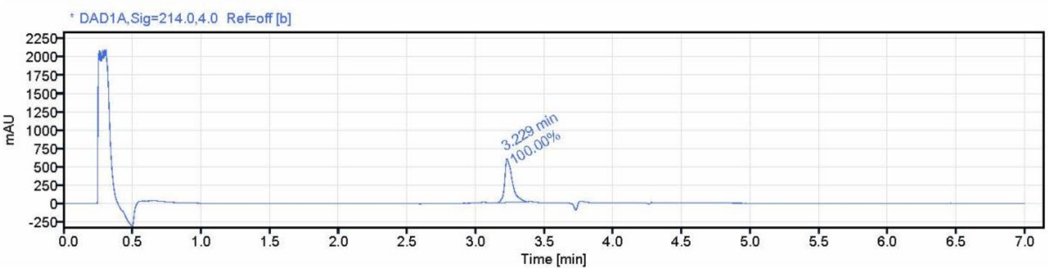

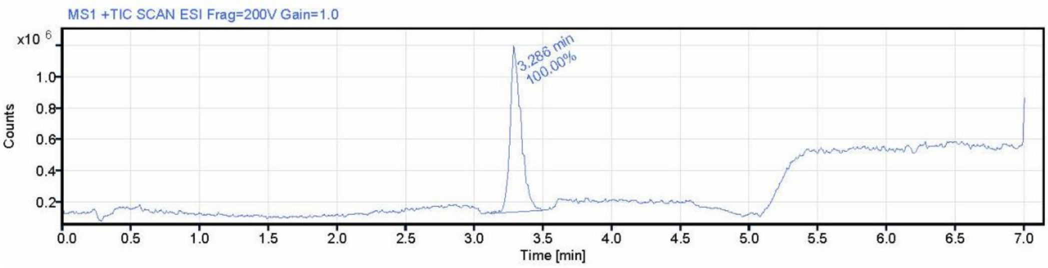

Peak Retention Time 3.286

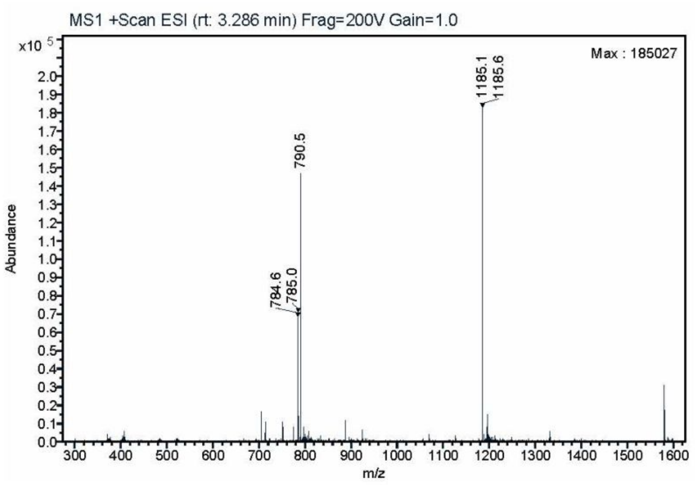

Expected MW = 2368.79 g/mol

Peptide 3/4c (> 99% purity)

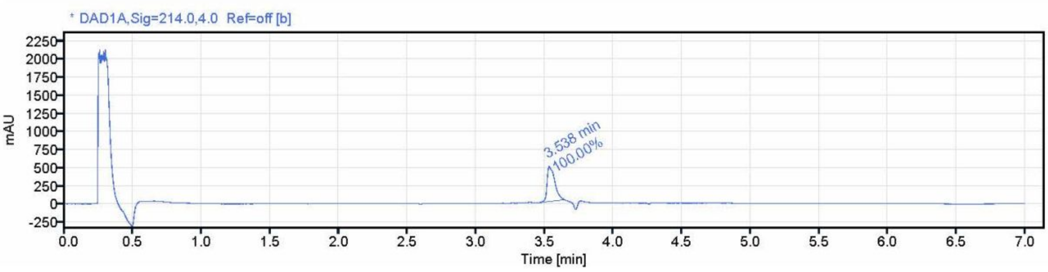

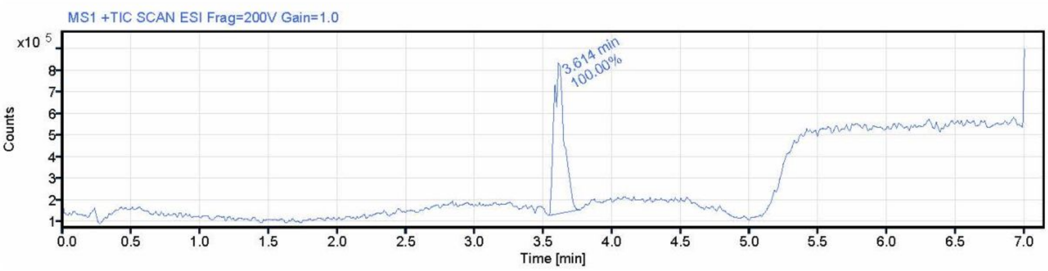

Peak Retention Time 3.614

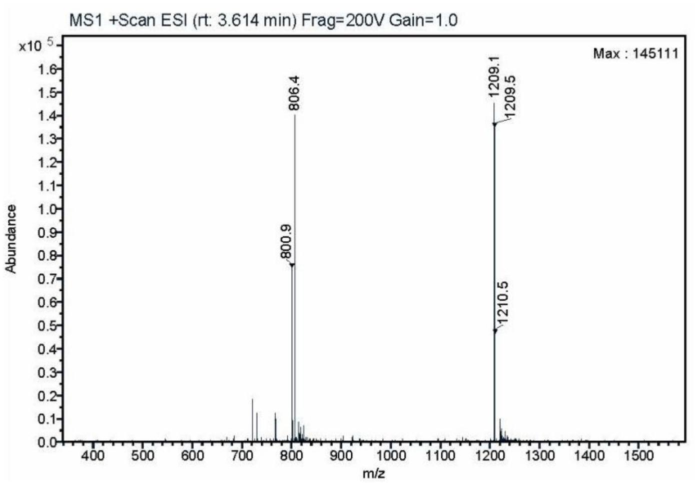

Expected MW = 2416.87 g/mol

Peptide 3/4d (> 99% purity)

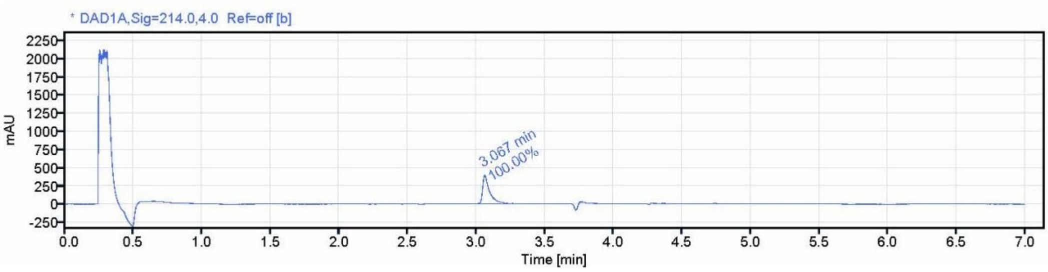

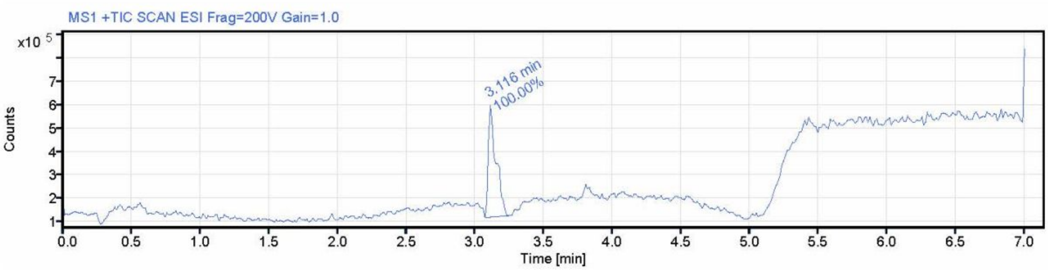

Peak Retention Time 3.116

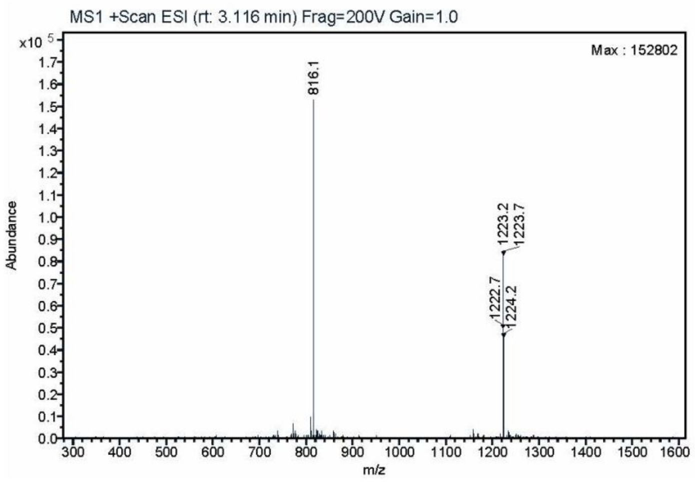

Expected MW = 2444.93 g/mol

Peptide 3/4e (> 99% purity)

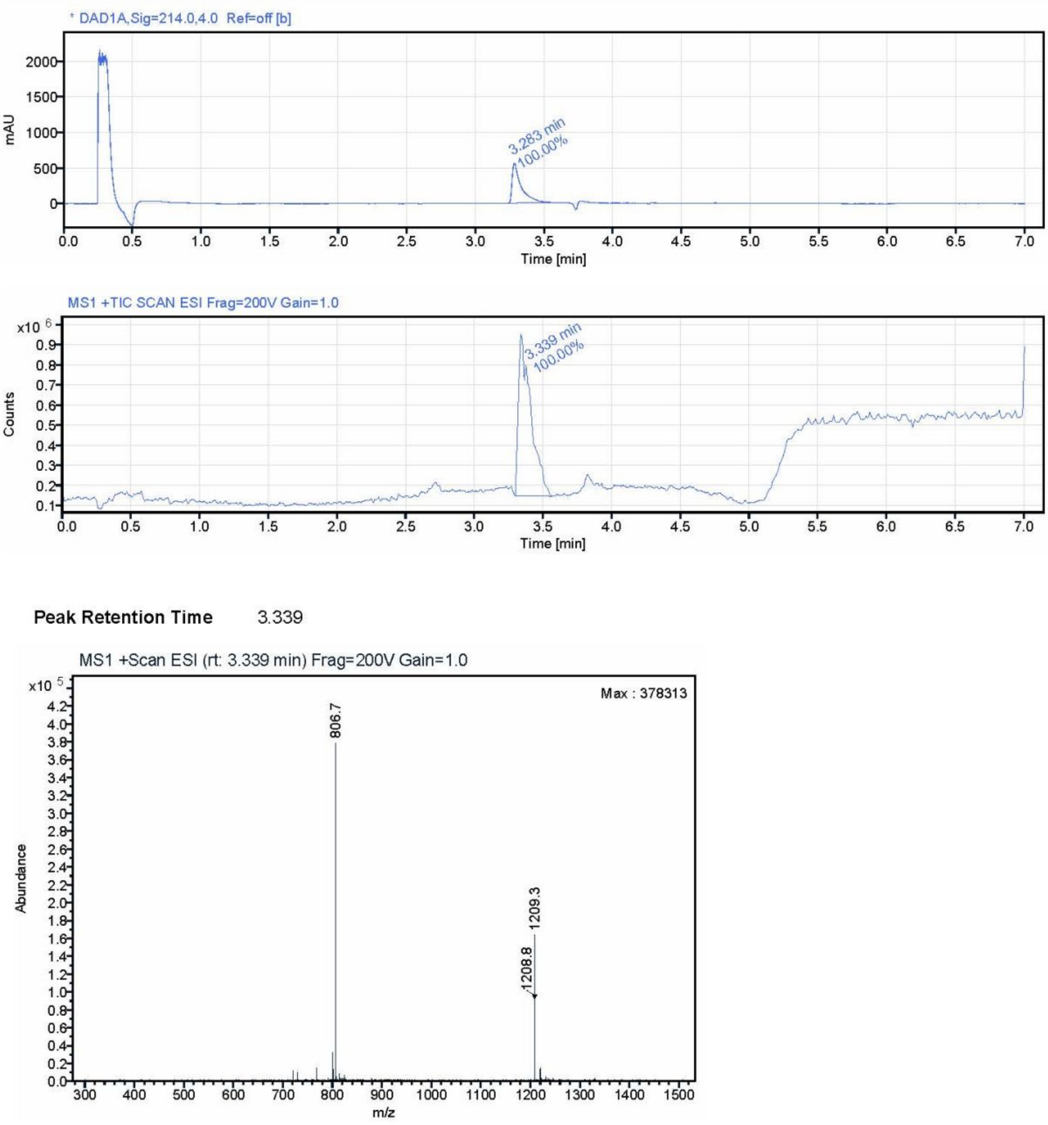

Expected MW = 2416.88 g/mol

Peptide 3/4f (> 99% purity)

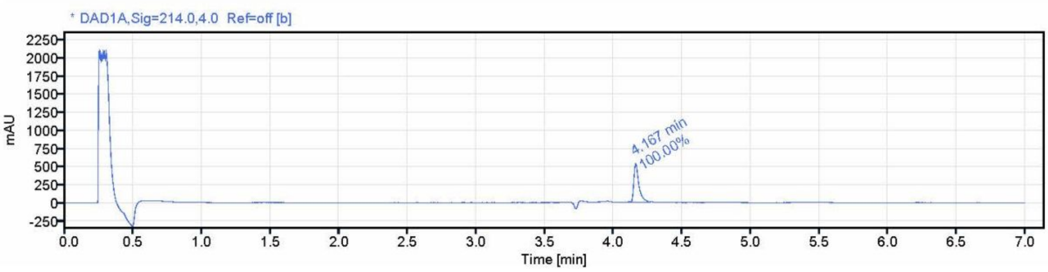

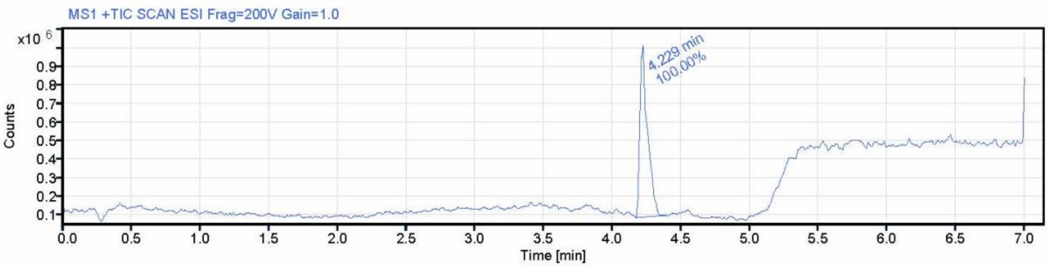

Peak Retention Time 4.229

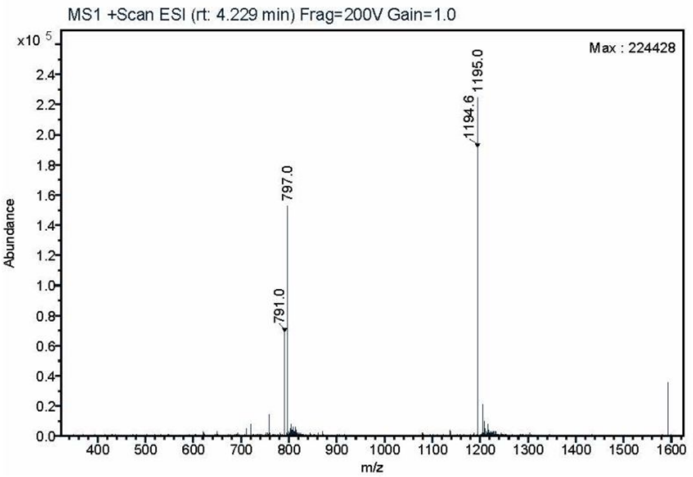

Expected MW = 2387.84 g/mol

Peptide 3/4g (> 99% purity)

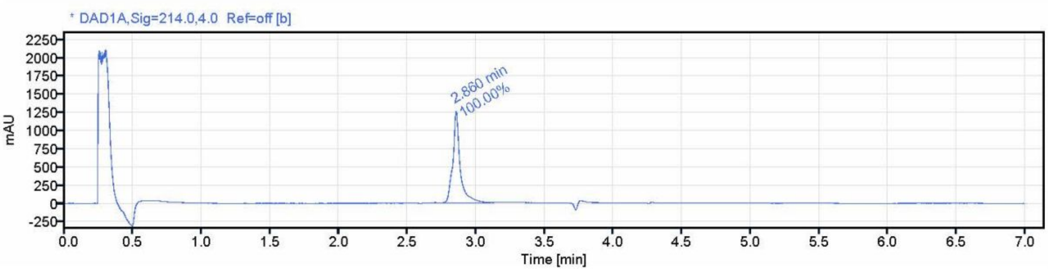

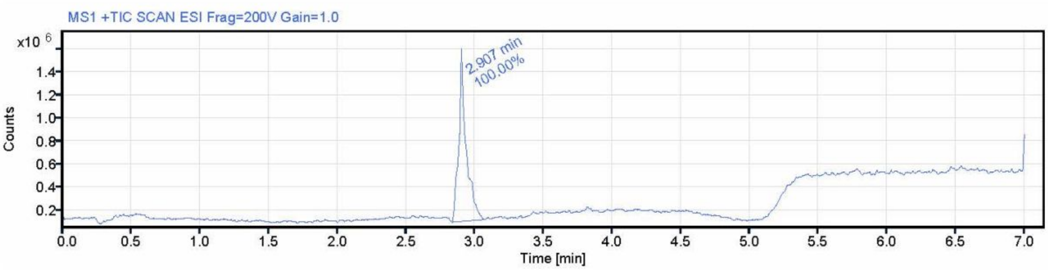

Peak Retention Time 2.907

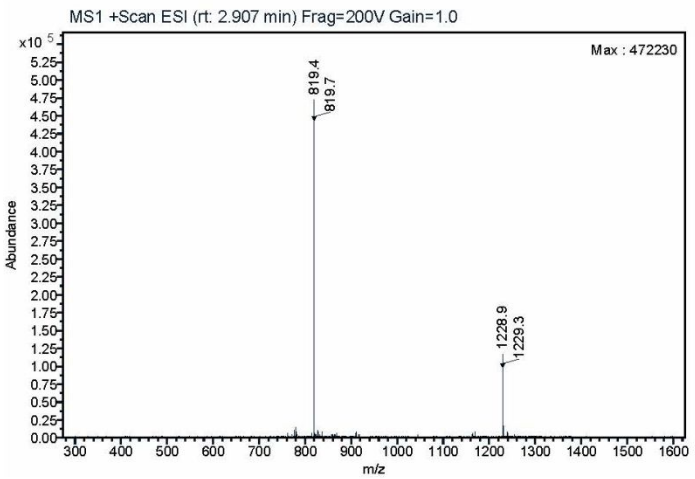

Expected MW = 2458.8 g/mol

Peptide 3/4h (97.43% purity)

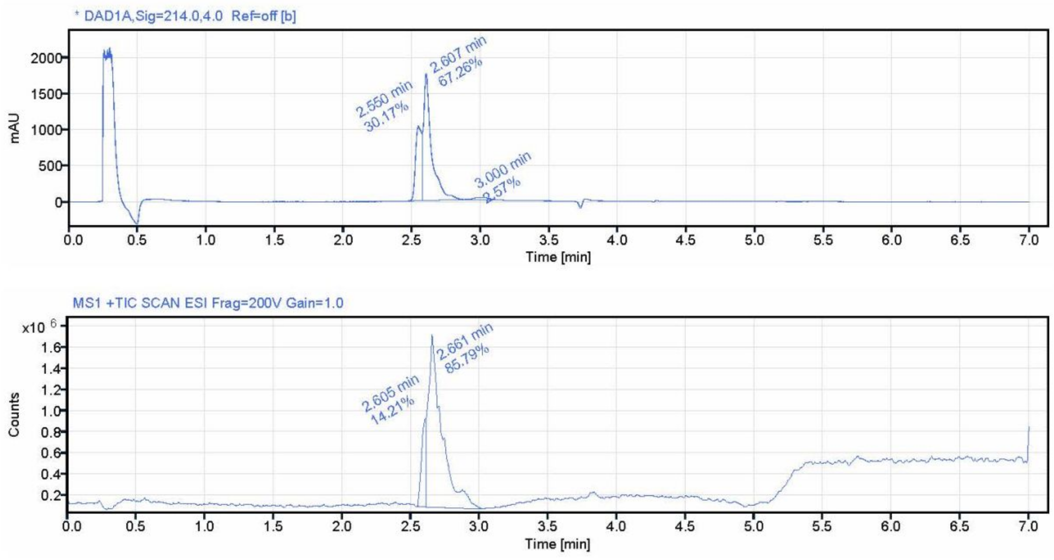

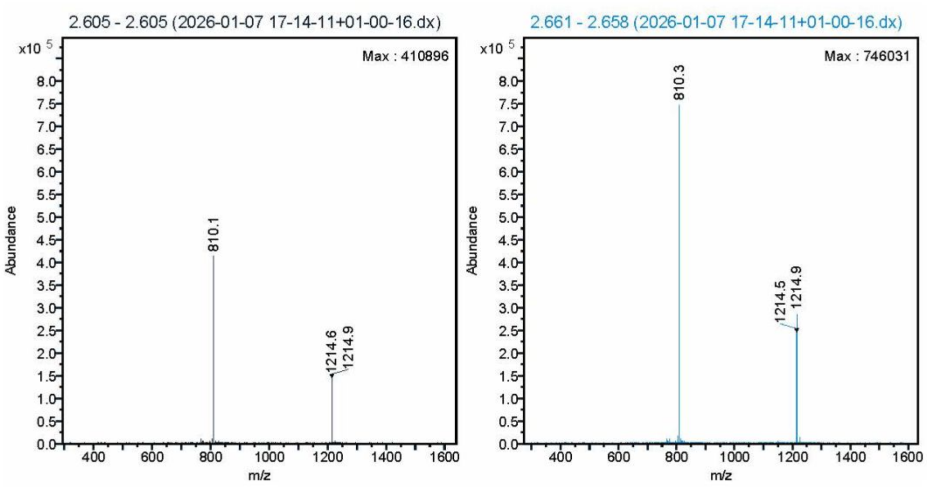

Expected MW = 2430.9 g/mol

Peptide 3/4j (> 99% purity)

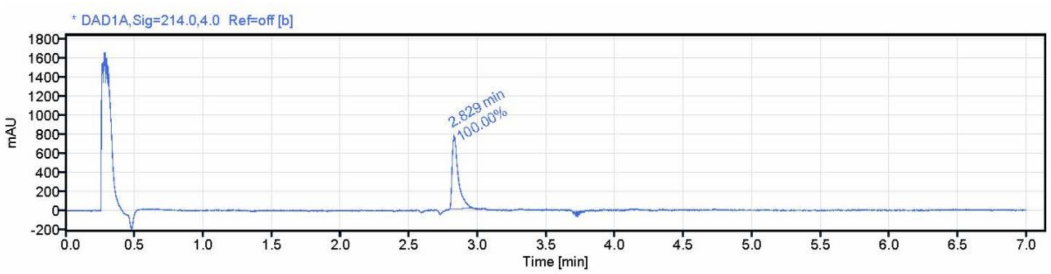

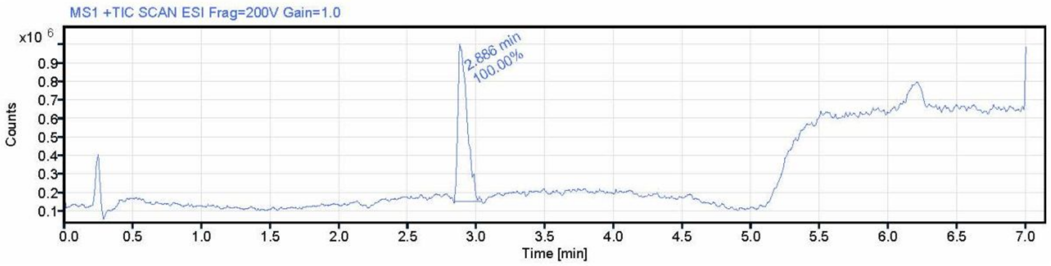

Peak Retention Time 2.886

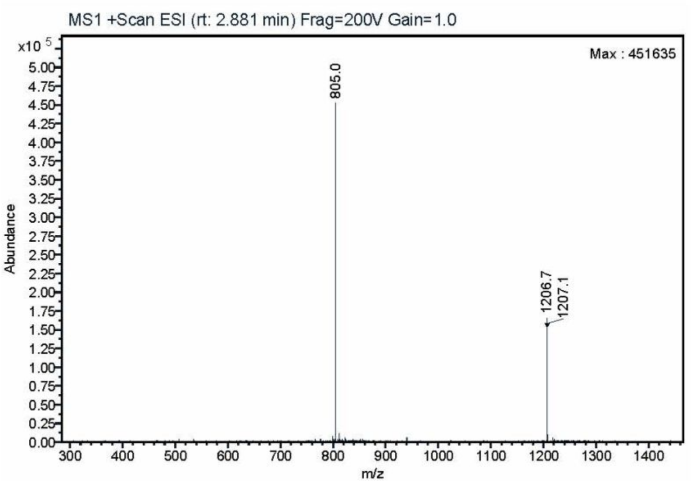

Expected MW = 2411.83 g/mol

Peptide 3/4k (> 99% purity)

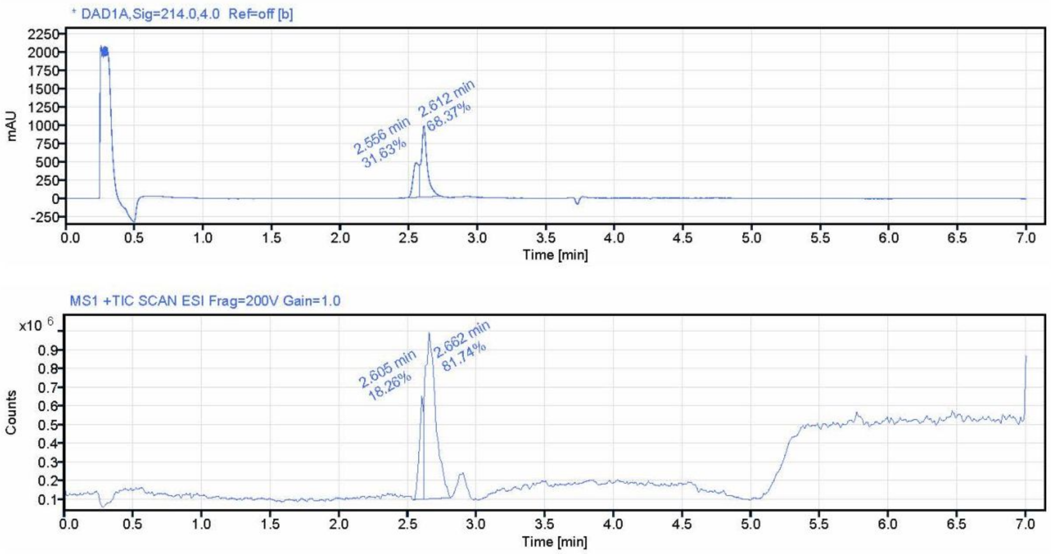

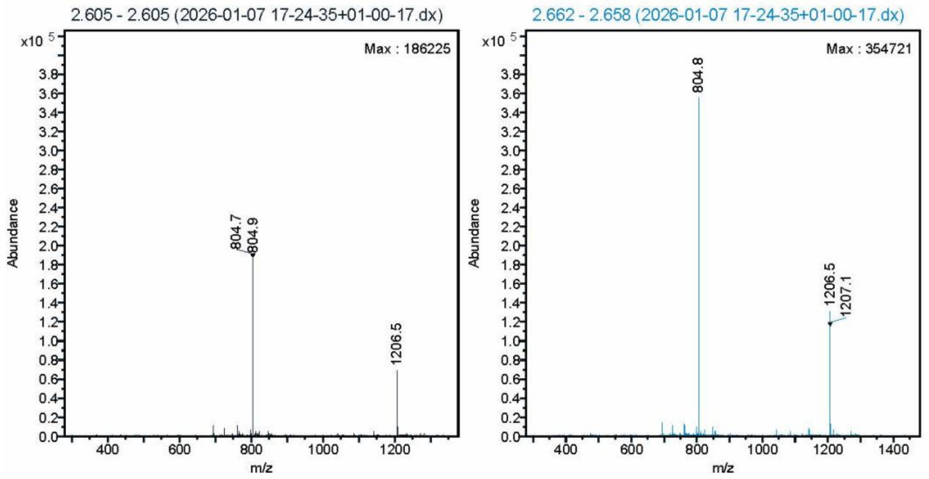

Expected MW = 2414.9 g/mol

Peptide 3/4l (> 99% purity)

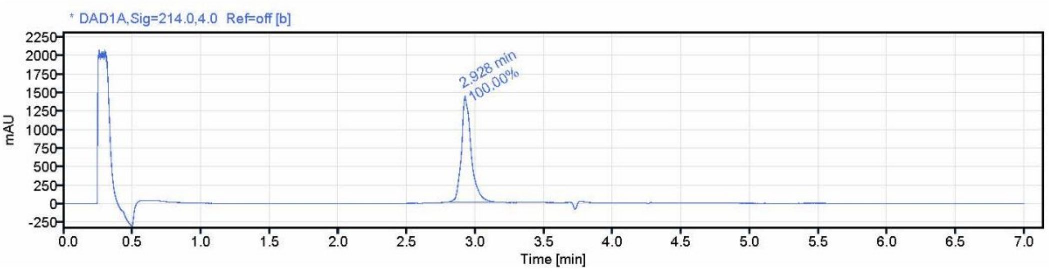

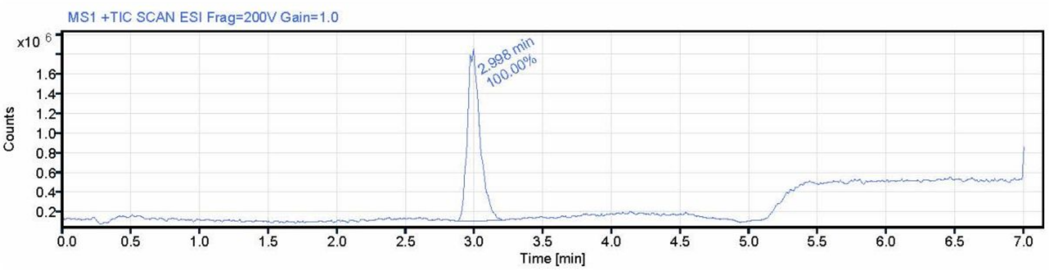

Peak Retention Time 2.998

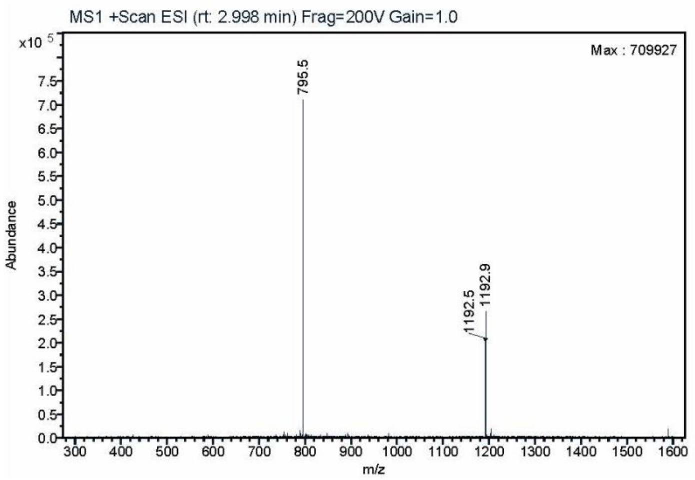

Expected MW = 2386.8 g/mol

Peptide 3/4n (> 95% purity)

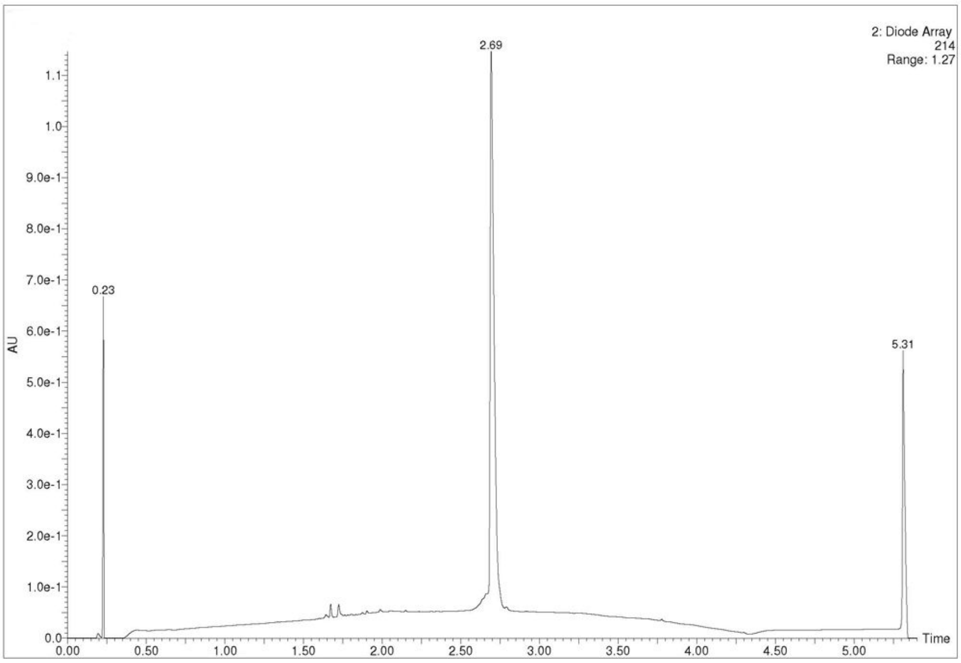

Expected MW = 2367.86 g/mol

Peptide 3/4o (> 99% purity)

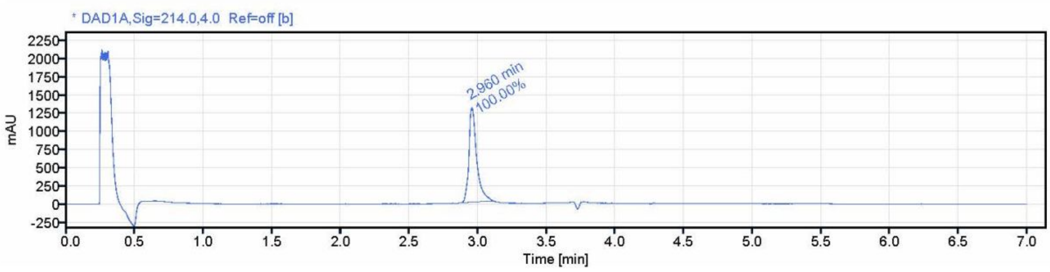

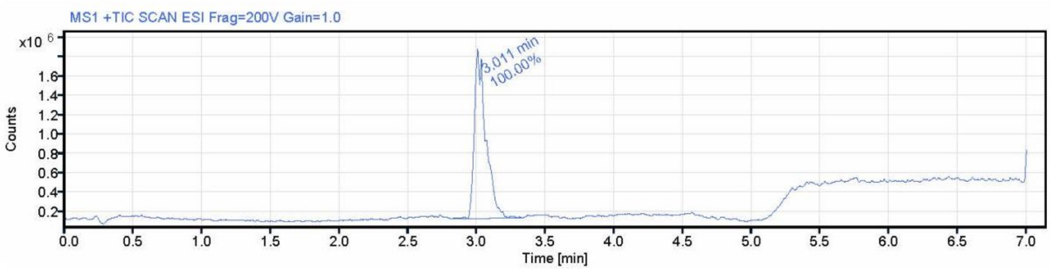

Peak Retention Time 3.011

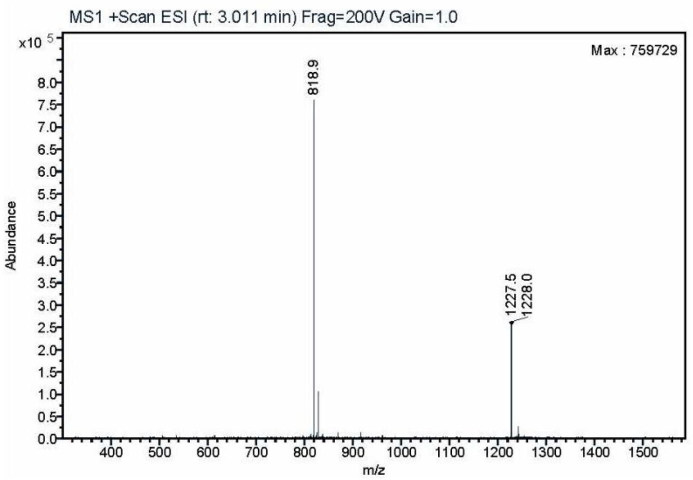

Expected MW = 2453.91 g/mol

Peptide 3/4p (> 99% purity)

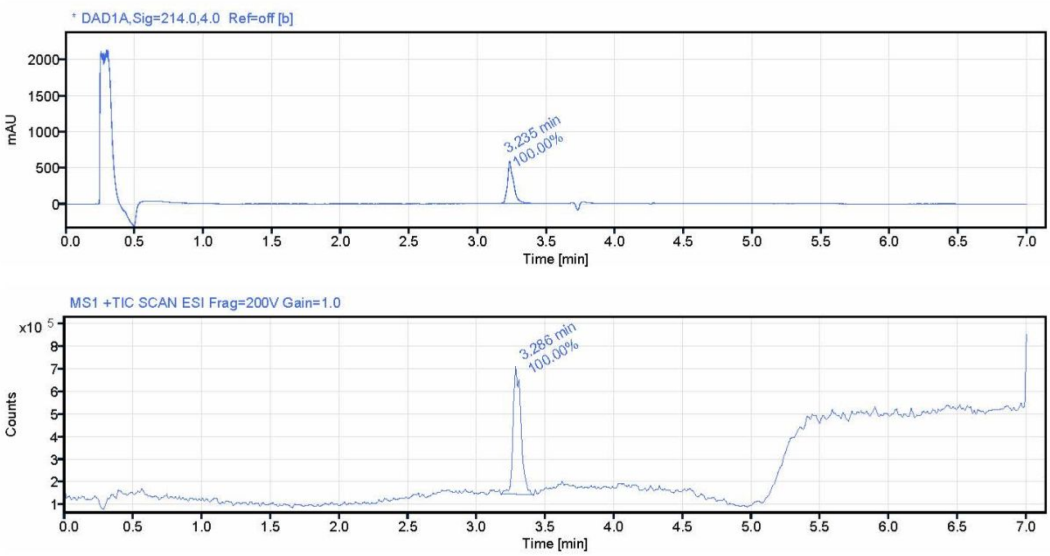

Peak Retention Time 3.286

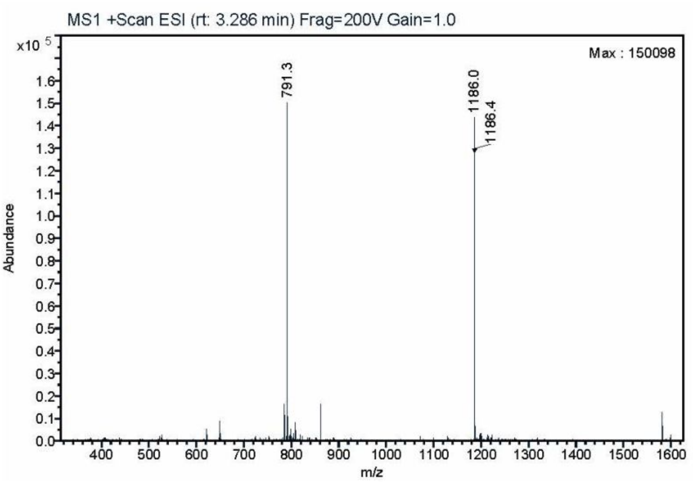

Expected MW = 2370.72 g/mol

Peptide 3/4q (> 99% purity)

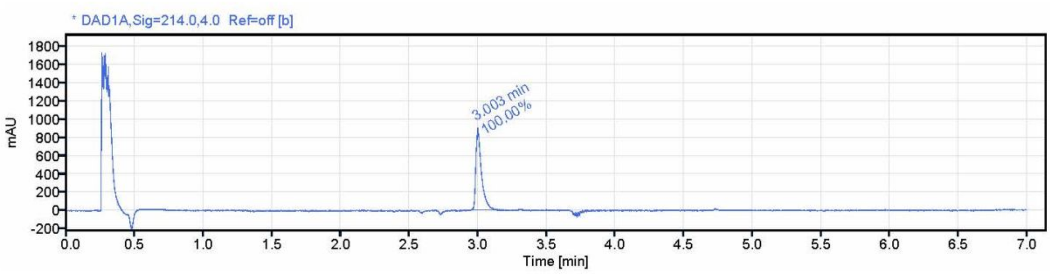

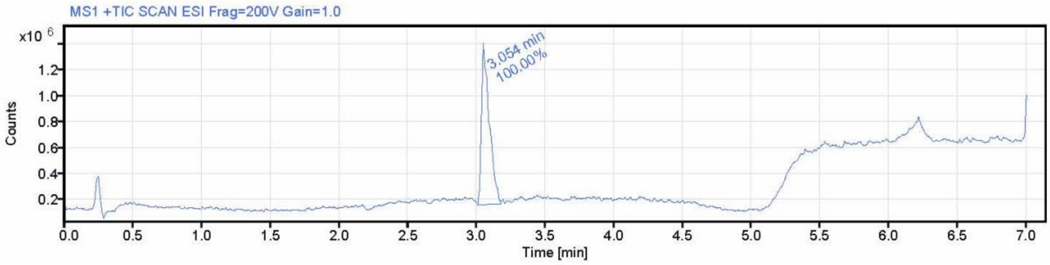

Peak Retention Time 3.054

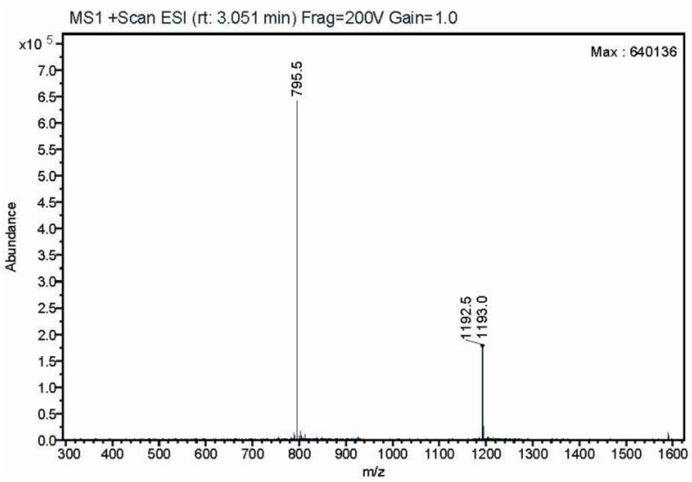

Expected MW = 2383.81 g/mol

Peptide 4/4a (97.71% purity)

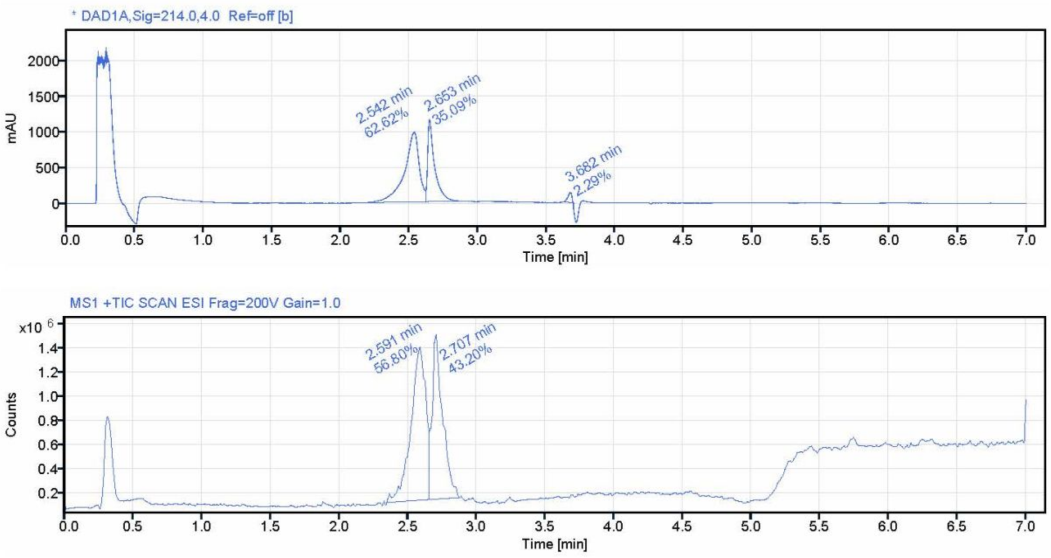

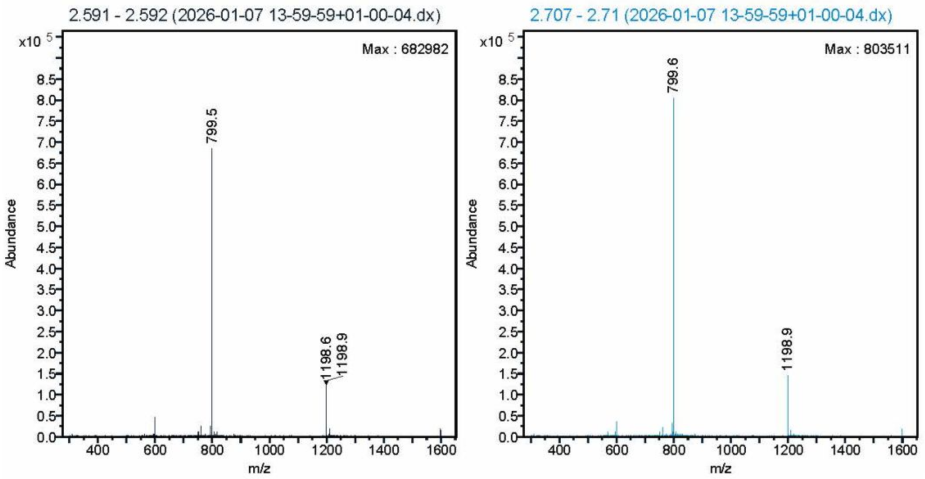

Expected MW = 2395.92 g/mol

Peptide 4/4b (> 99% purity)

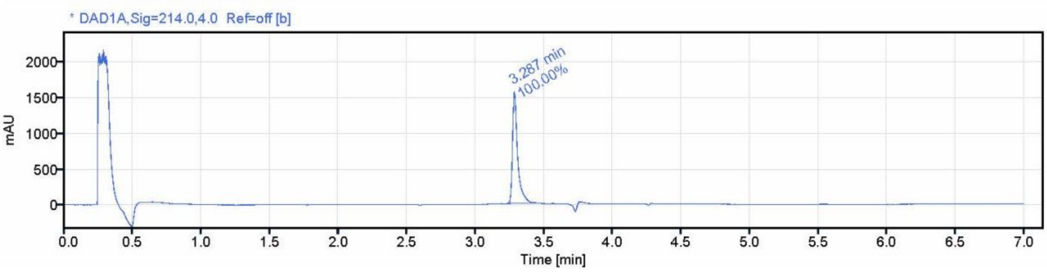

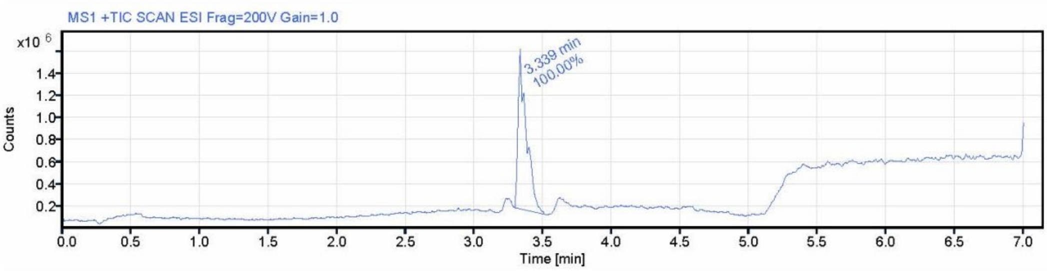

Peak Retention Time 3.339

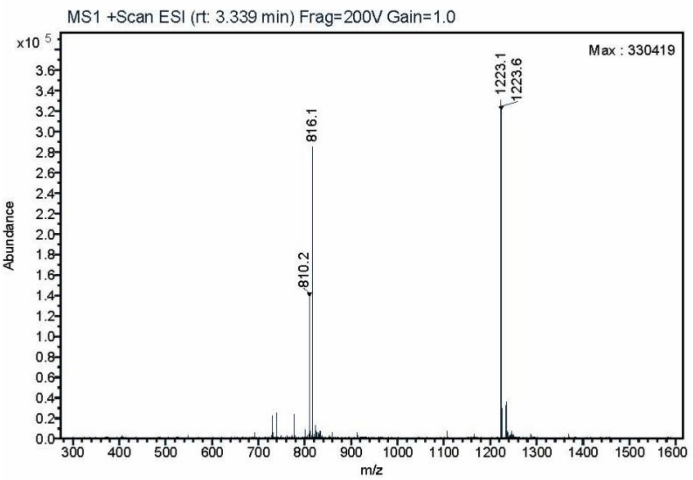

Expected MW = 2444.89 g/mol

Peptide 4/4c (> 99% purity)

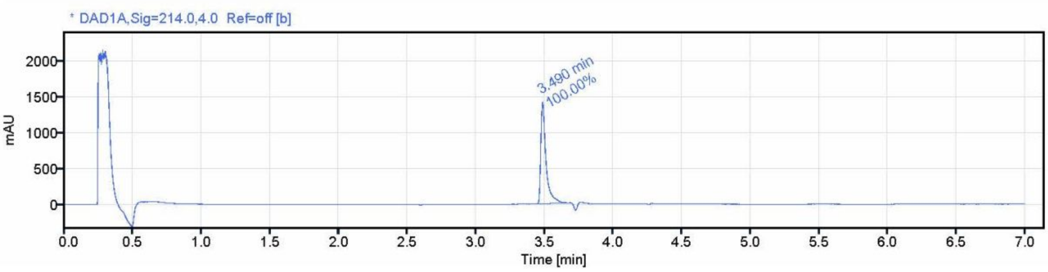

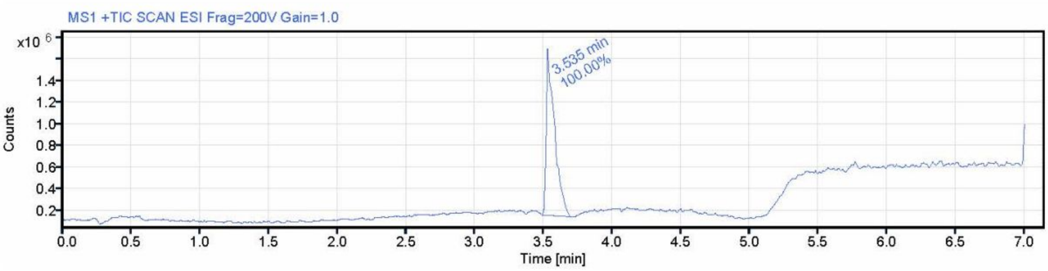

Peak Retention Time 3.535

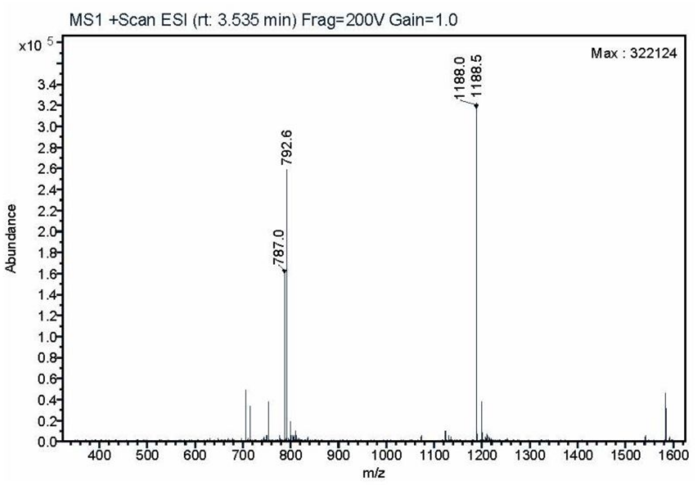

Expected MW = 2374.79 g/mol

Peptide 4/4d (> 99% purity)

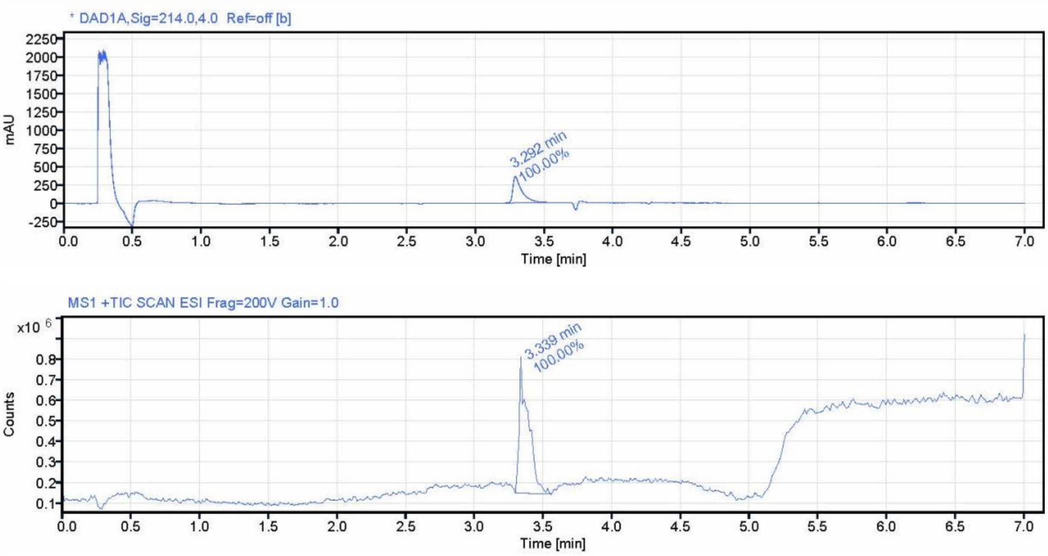

Peak Retention Time 3.339

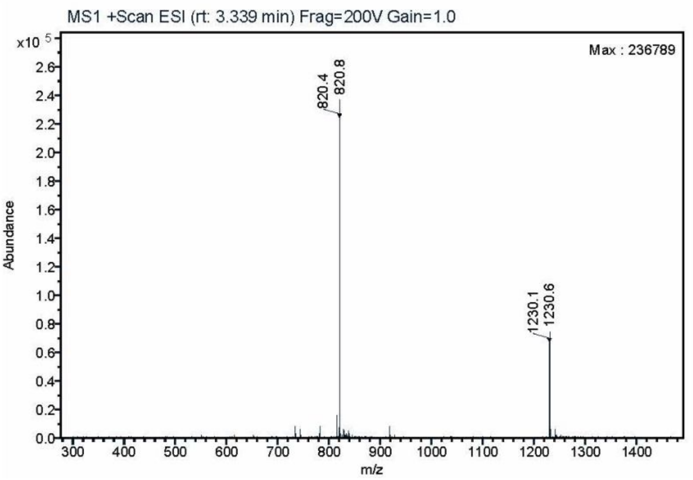

Expected MW = 2458.96 g/mol

Peptide 4/4e (> 99% purity)

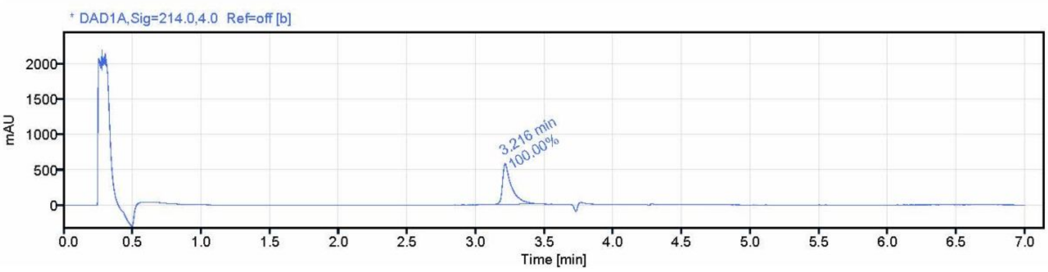

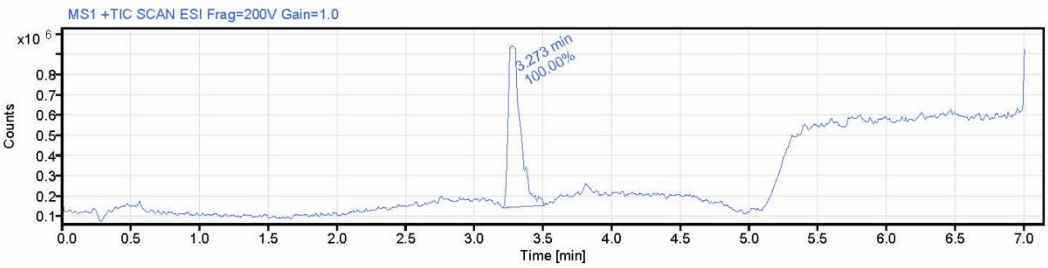

Peak Retention Time 3.273

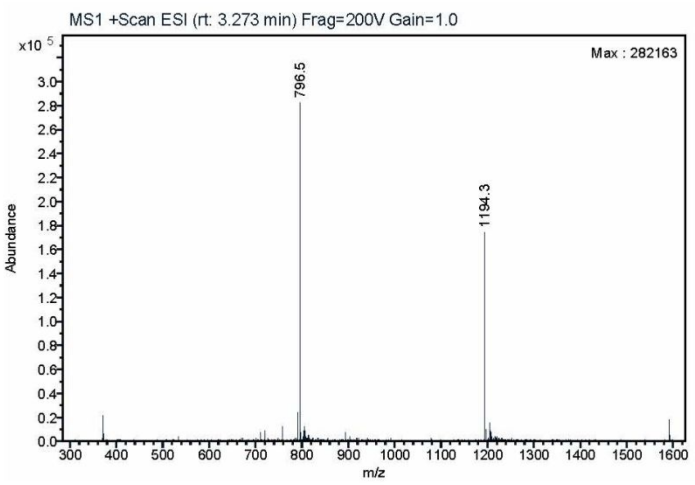

Expected MW = 2386.9 g/mol

Peptide 4/4f (> 99% purity)

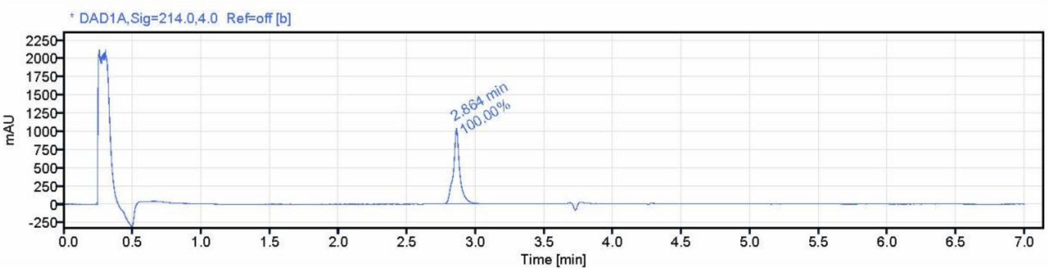

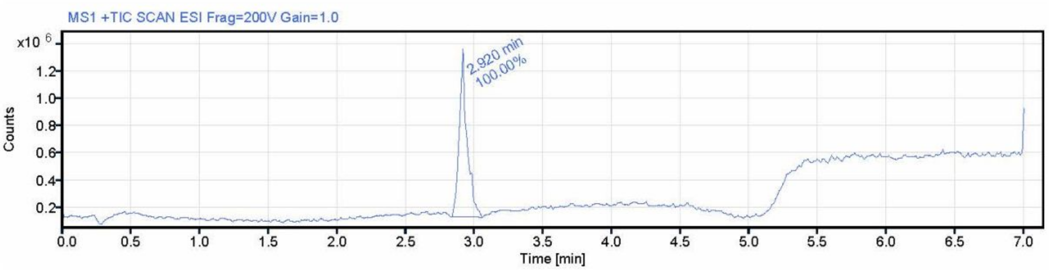

Peak Retention Time 2.920

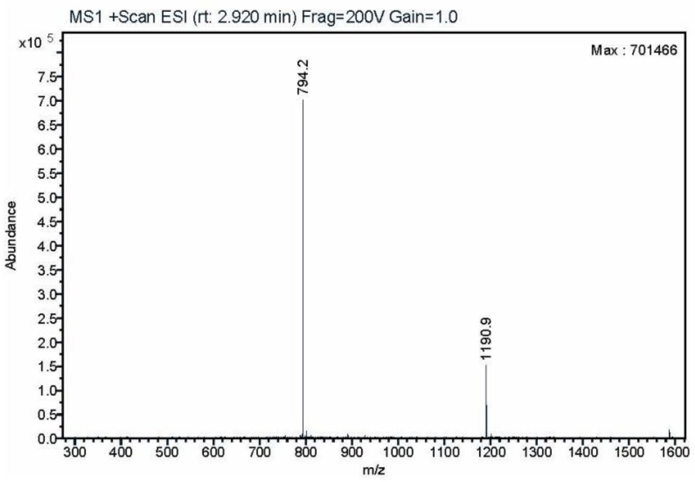

Expected MW = 2379.73 g/mol

Peptide 4/4h (> 99% purity)

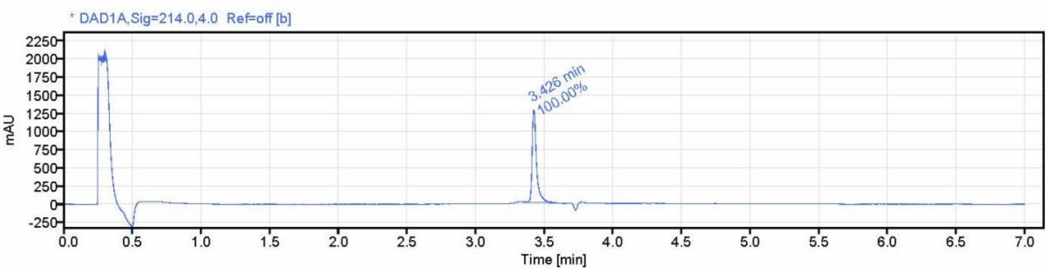

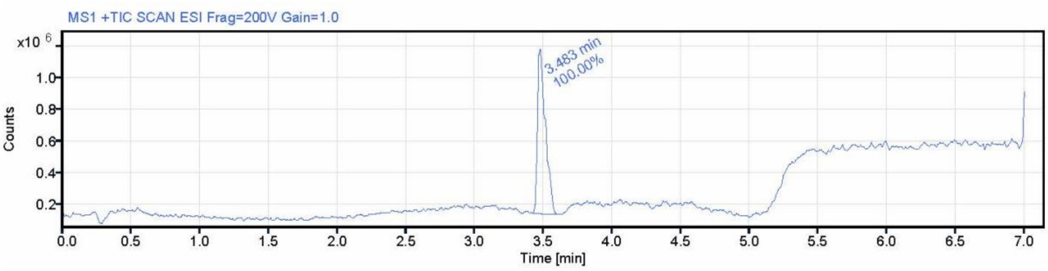

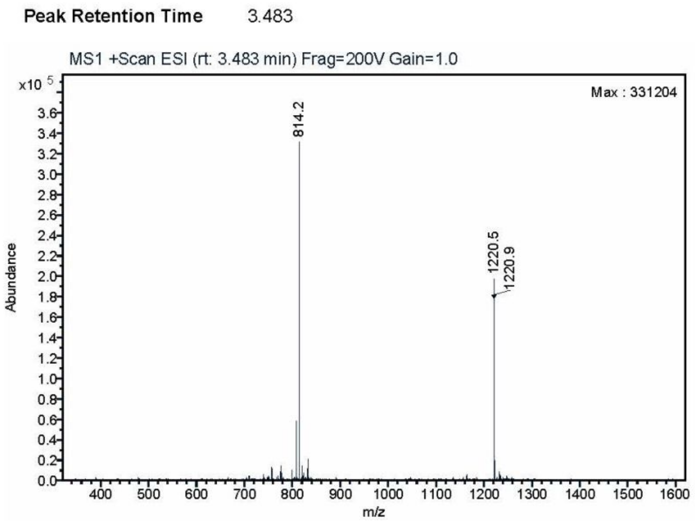

Expected MW = 2439.79 g/mol

Peptide 4/4i (> 95% purity)

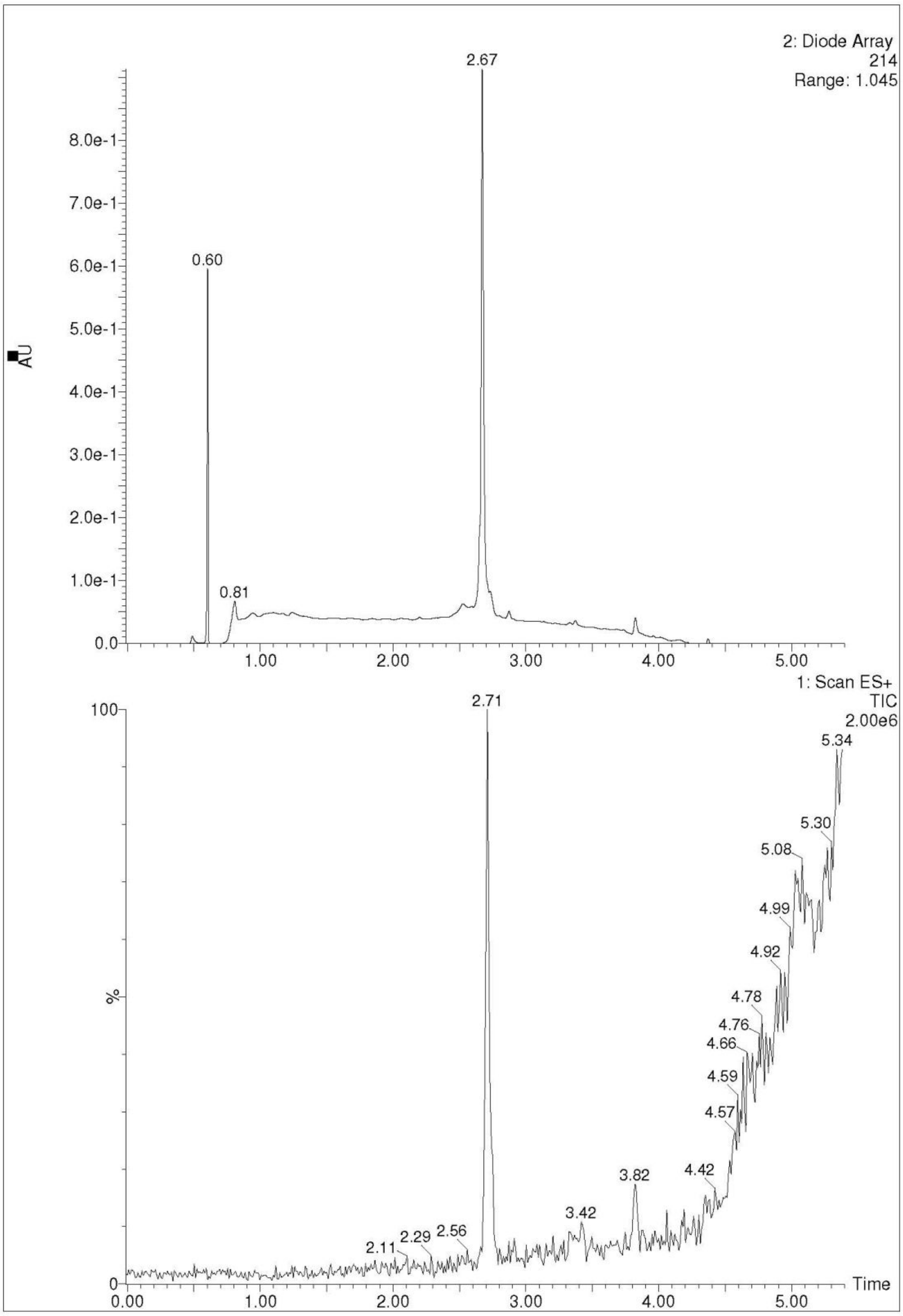

Expected MW = 2397.8 g/mol

Peptide 4/4j (86.98 % purity)

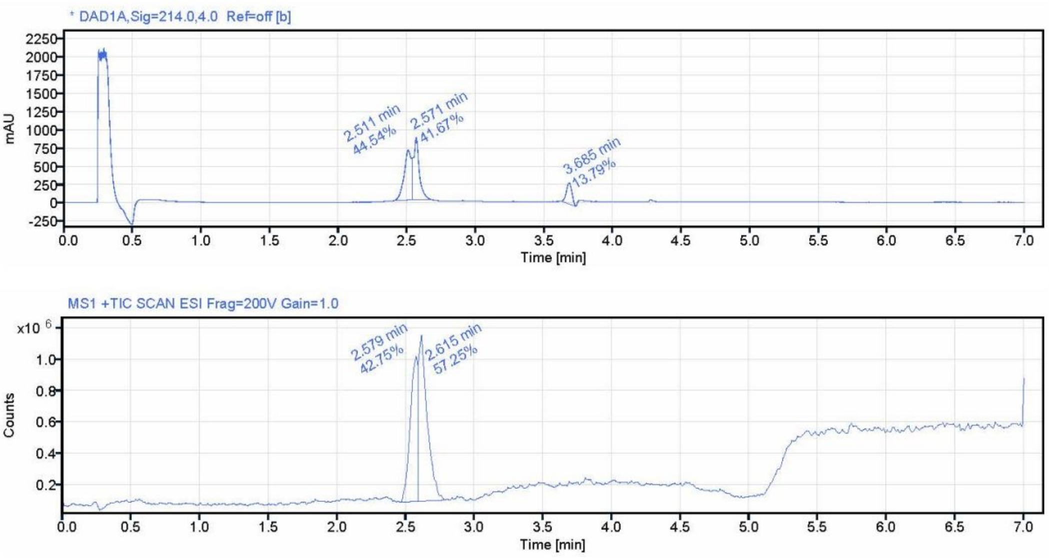

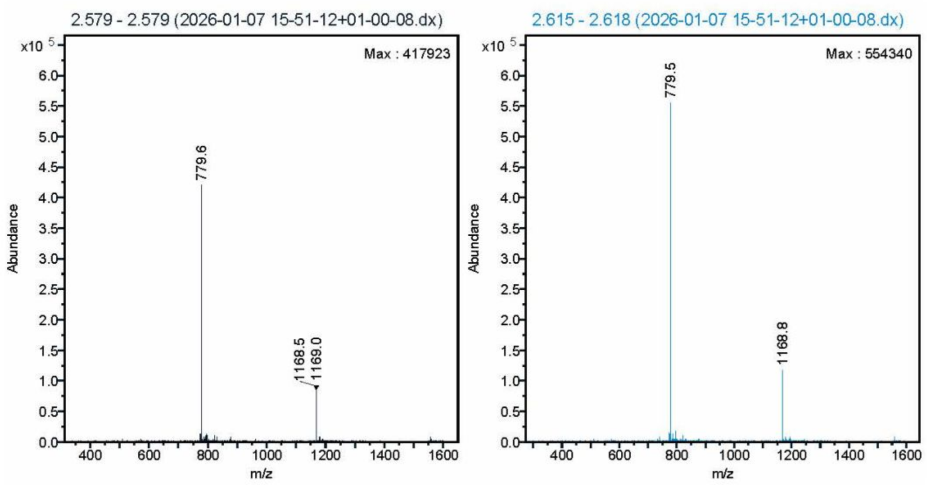

Expected MW = 2335.76 g/mol

Peptide 4/4k (> 99% purity)

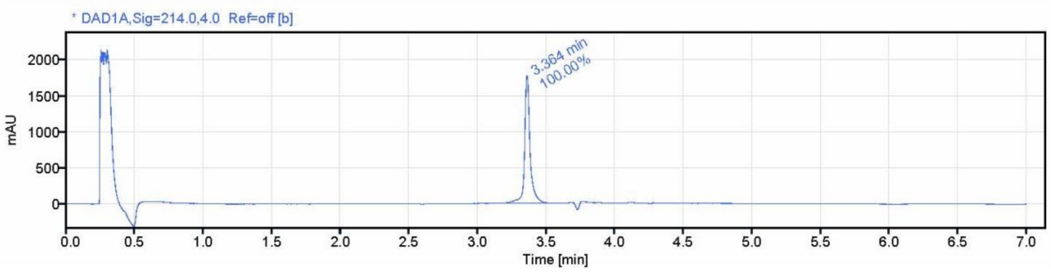

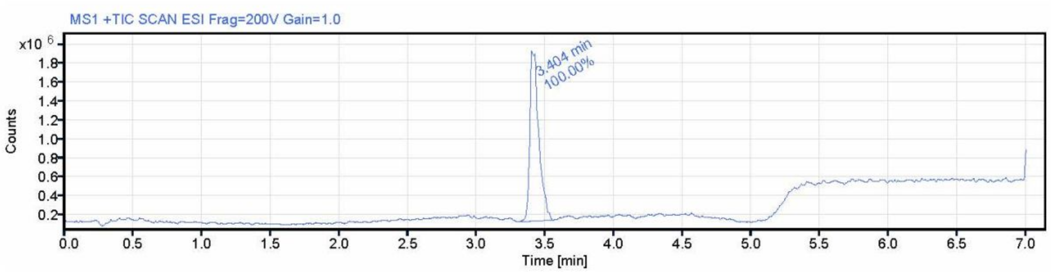

Peak Retention Time 3.404

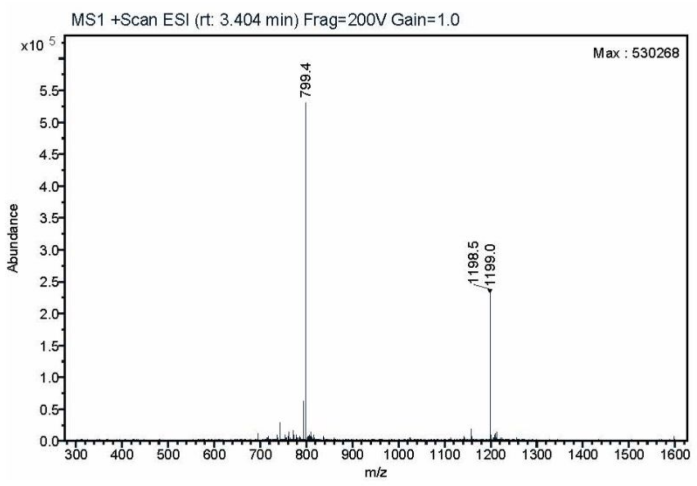

Expected MW = 2395.82 g/mol

Peptide 4/4l (> 99% purity)

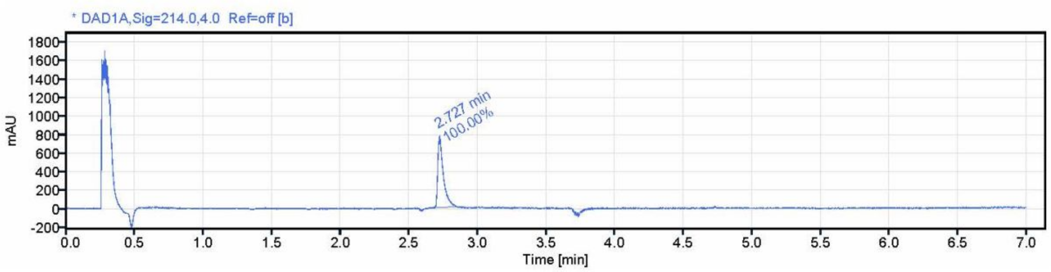

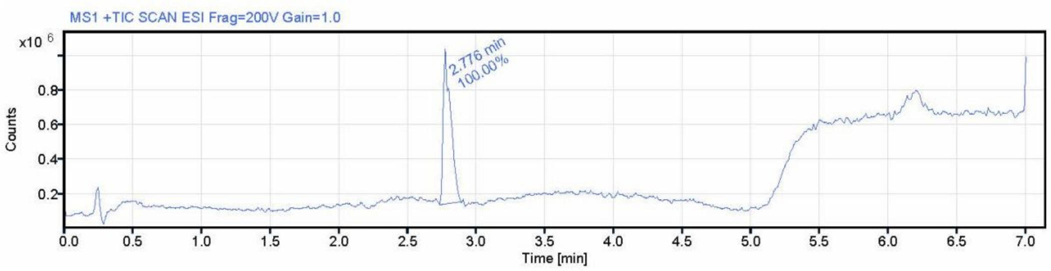

Peak Retention Time 2.776

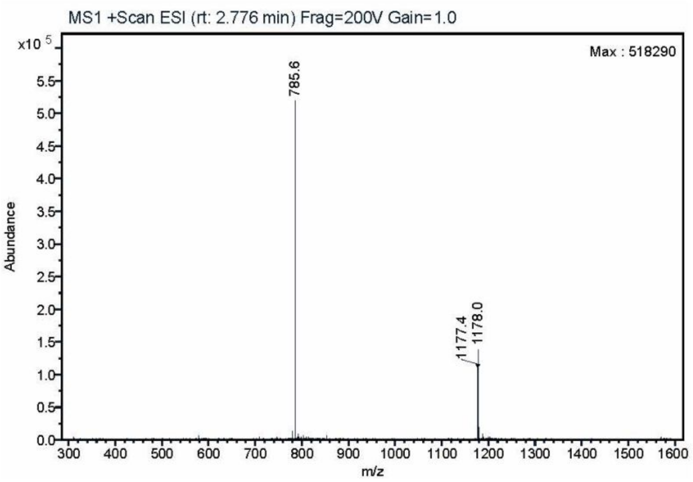

Expected MW = 2353.83 g/mol

Peptide 4/4m (> 99% purity)

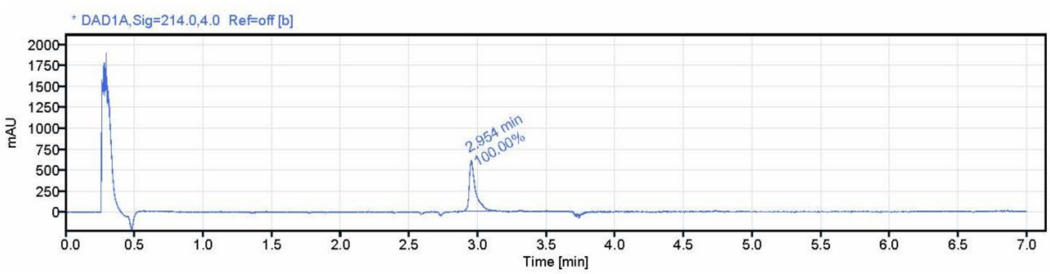

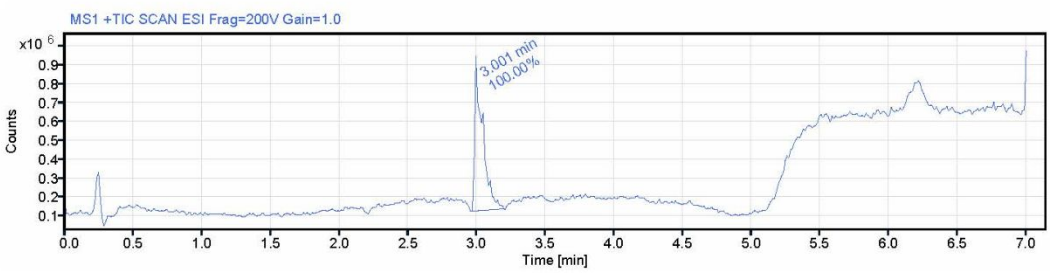

Peak Retention Time 3.001

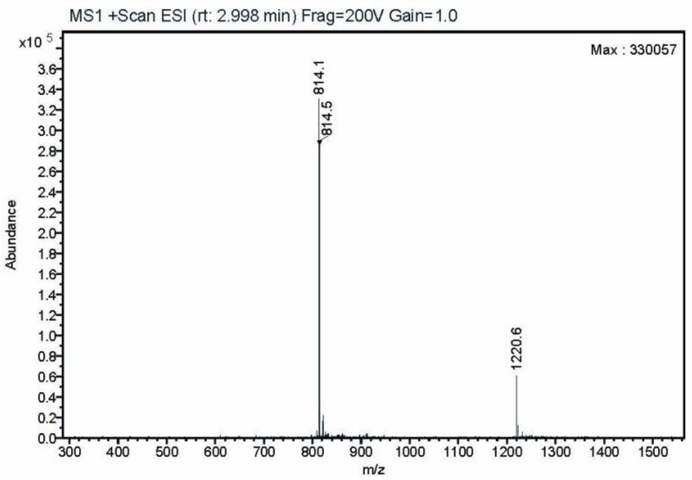

Expected MW = 2439.88 g/mol

Peptide 4/4n (> 99 % purity)

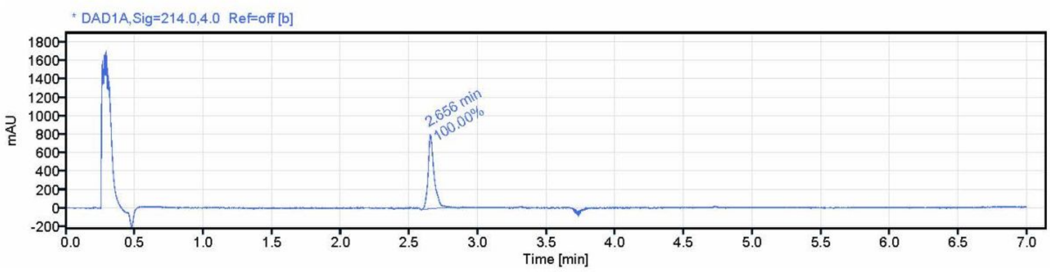

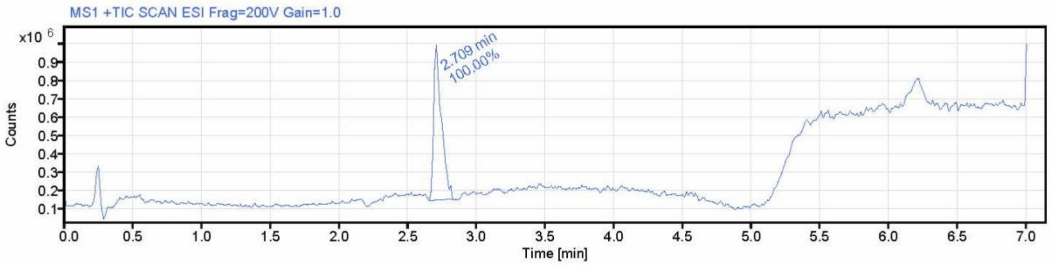

Peak Retention Time 2.709

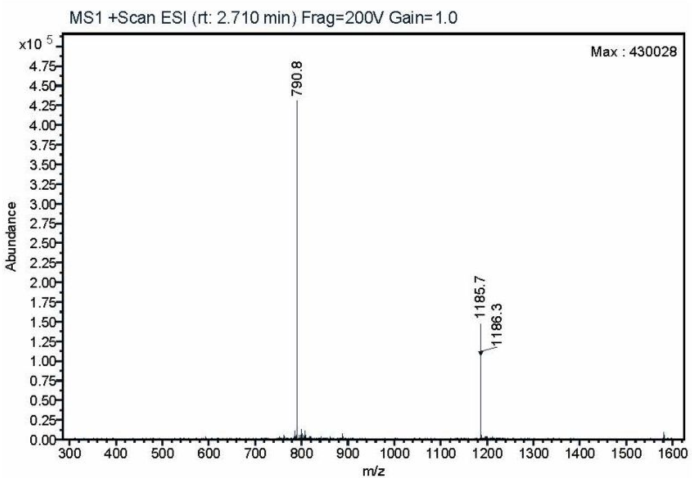

Expected MW = 2369.78 g/mol

REFERENCES

1. Meng, E. C.; Goddard, T. D.; Pettersen, E. F.; Couch, G. S.; Pearson, Z. J.; Morris, J. H.; Ferrin, T. E., UCSF ChimeraX: Tools for structure building and analysis. *Protein Sci* **2023,** *32* (11), e4792.
2. Mills, J. E.; Dean, P. M., Three-dimensional hydrogen-bond geometry and probability information from a crystal survey. *J Comput Aided Mol Des* **1996,** *10* (6), 607-22.
